# ACPYPE - AnteChamber PYthon Parser interfacE

**DOI:** 10.1186/1756-0500-5-367

**Published:** 2012-07-23

**Authors:** Alan W Sousa da Silva, Wim F Vranken

**Affiliations:** 1 Department of Biochemistry, University of Cambridge, 80 Tennis Court Road, Cambridge, CB2 1GA, UK; 2 Protein Data Bank in Europe (PDBe), EMBL-EBI, European Bioinformatics Institute, Wellcome Trust Genome Campus, Hinxton, Cambridge, CB10 1SD, UK; 3 Present address: Universal Protein Resource (UniProt), EMBL-EBI, European Bioinformatics Institute, Wellcome Trust Genome Campus, Hinxton, Cambridge, CB10 1SD, UK; 4 Present address: Department of Structural Biology, VIB and Structural Biology Brussels, Vrije Universiteit Brussel, Pleinlaan 2, 1050 Brussel, Belgium

**Keywords:** MD, GROMACS, AMBER, CNS, ANTECHAMBER, NMR, Ligand, Topology

## Abstract

**Background:**

ACPYPE (or AnteChamber PYthon Parser interfacE) is a wrapper script around the ANTECHAMBER software that simplifies the generation of small molecule topologies and parameters for a variety of molecular dynamics programmes like GROMACS, CHARMM and CNS. It is written in the Python programming language and was developed as a tool for interfacing with other Python based applications such as the CCPN software suite (for NMR data analysis) and ARIA (for structure calculations from NMR data). ACPYPE is open source code, under GNU GPL v3, and is available as a stand-alone application at http://www.ccpn.ac.uk/acpype and as a web portal application at http://webapps.ccpn.ac.uk/acpype.

**Findings:**

We verified the topologies generated by ACPYPE in three ways: by comparing with default AMBER topologies for standard amino acids; by generating and verifying topologies for a large set of ligands from the PDB; and by recalculating the structures for 5 protein–ligand complexes from the PDB.

**Conclusions:**

ACPYPE is a tool that simplifies the automatic generation of topology and parameters in *different formats* for *different* molecular mechanics programmes, including *calculation of partial charges*, while being *object oriented* for integration with other applications.

## Findings

**Figure 1:**
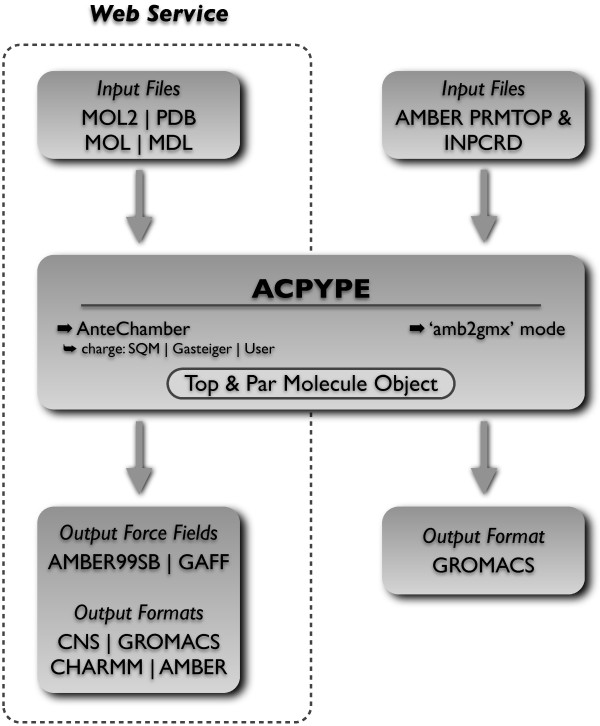
**Workflow diagram for ACPYPE.** Diagram depicting the general scheme of how ACPYPE works. Encompassed by the traced line is the ACPYPE functionality already implemented as a web service at http://webapps.ccpn.ac.uk/acpype.

Here we introduce ACPYPE, a tool based on ANTECHAMBER [1] for generating automatic topologies and parameters in different formats for different molecular mechanics programmes, including calculation of partial charges. In other to validate ACPYPE, we verified its topologies generated in three detailed ways: 1) by comparing with default AMBER [2] topologies for standard amino acids; 2) by generating and verifying topologies for a large set of ligands from the Protein Data Bank (PDB) [3]); and 3) by recalculating the structures for 5 protein–ligand complexes from the PDB. The Figure 1 summarises its resources and features, giving a general overview of how ACPYPE works.

## Background

Molecular Mechanics (MM) has evolved substantially over the last decades, not only because of major advances in computational power, but also due to more accurate and diverse force field descriptions. Molecular Dynamics (MD) and NMR Structure Calculation software (NMR-SC) have matured in line with these advances in MM to become more complex, faster and precise; MD and NMR-SC software packages can now perform calculations that were previously thought to be very difficult to handle [4].

Such calculations, however, always depend on a precise and complete description of the topology and physical parameters of the molecules they tackle. The methods to obtain these descriptions are well developed for common bio-molecular components like amino acids and nucleic acids, but reliable and automatic procedures to obtain this information for heterogeneous chemical compounds are scarce. Researchers trying to address, for example, protein–ligand complexes often have to manually create the topologies for their ligands, a procedure which creates additional overhead and which often results in errors in the final coordinate files (as evidenced by many ligand errors in entries in the PDB).

ACPYPE resulted from our need to find a solution to this problem for NMR-SC using the CNS software [5]: the simulated annealing (SA) and water refinement (WR) procedures for protein-ligand complexes require a full topological description of the ligand, including hydrogens to handle interatomic distance restraints from NMR. We first explored a host of existing solutions; unfortunately none of them generated the required topologies. CNS and XPLOR (including its variant XPLOR-NIH [6,7]) have a function called LEARn that only generates parameter information, no topology nor charges. XPLO2D [8], one of the first tools to address the problem of generating topological parameters for small molecules, also does not calculate charges. Both approaches are not amenable for the now almost mandatory final water refinement step in an NMR structure calculation protocol. A more recent and well-known application is PRODRG [9]. However, in order to speed up calculations PRODRG uses the concept of “united-atoms” where no explicit hydrogen atoms are present, and its topologies are unsuitable for all-atom force fields and water refinement. The GlyCaNS [10] tool generates the required topological parameters in CNS format but has limited scope as it only works for polysaccharides. The MKTOP program [11] can define atom types and hence topological parameters, but it cannot derive partial charges and only recently became able to generate topologies for AMBER03 [12] force field (besides the OPLS/AA [13]). Finally, the recently developed Automated Topology Builder (ATB) [14] is limited in scope because it only generates topologies compatible with the GROMOS 53A6 [15] force field.

The tool we identified as having the most relevant functionality was ANTECHAMBER [1]. It is the main tool for creating variants in AMBER force fields [16], has foundations in quantum mechanics rather than empirical data, and is iteratively improved based on experience from previous force fields implementations. It is already used to automatically generate topologies with the General Amber Force Field (GAFF [17]), and although AMBER force fields are ported to CNS/XPLOR [5,7], the ANTECHAMBER output has to be interpreted and converted before it becomes useful. A similar tool called CGenFF [18] generates CHARMM General Force Field topology for small molecules, but is more recent and does not have as wide a user base. We therefore chose ANTECHAMBER as the starting point for ACPYPE, with the aim to facilitate and automate its operation for non-AMBER users, as well as extending its use to other Python based applications.

ACPYPE is already successfully used in the scientific community; it is released under the open source GNU GPL version 3 license, is freely available, and offers a reliable solution for generating topologies and parameters for small chemical compounds in all-atom force fields in the following platforms: CNS/XPLOR, GROMACS and CHARMM [19]. It also automates several steps necessary to create a library for a small molecule for the AMBER package. The topologies generated by ACPYPE can be further used in AMBER force fields as ported to GROMACS (*viz.* ffAMBER [20]), CNS/XPLOR, NAMD [21] and CHARMM) without breaking the compatibility of the force field. ACPYPE is object oriented and uses an API library that can be easily extended, so new routines for as yet unsupported MD packages are easily added.

## Methods

### Implementation

ACPYPE collects information about the molecular system from the input molecular coordinate file and from the topology and parameters as generated by ANTECHAMBER and the tleap, sleap or xleap AMBER tools. It then creates a Python object where all this information is combined (see Figure 1 for a general overview). ACPYPE requires Python 2.6 (or higher) and ANTECHAMBER (version from AmberTools12 is recommended, although it should work with older versions). OpenBabel [22,23] installation is optional but required for reading molecule information from PDB-style files. Python, ANTECHAMBER/AmberTools and OpenBabel are freely available.

ACPYPE is executed by the command ‘acpype [options]’, where the main options are: 

· **-i <filename>:** An input coordinate file is required in one of the following formats: MOL2, PDB or MDL.

· **-n [int]:** This option defines the net charge of the molecule. If not given, ACPYPE will use the Gasteiger method [24] to guess the charge. This is not a dependable procedure, however, and might result in an incorrect overabundancell charge.

· **-a [gaff | amber]:** GAFF is used by default. Option ‘amber’ will use a set of parameters merged from the highly developed force fields AMBER99SB [2] for proteins and AMBER99bsc0 [25] for nucleic acids. In case a parameter is not found for AMBER99SB, ACPYPE will fall back to GAFF definitions.

· **-c [bcc | gas | user]:** The semi-empirical quantum chemistry programme SQM [26] is used by default (via ANTECHAMBER) to determine the atomic partial charges. Option ‘gas’ will use the faster but less precise Gasteiger method, option ‘user’ will take partial charges as defined in a MOL2 file, which can be calculated using more sophisticated methods like R.E.D. [27,28] or the YASARA AutoSMILES Server [29] (see Additional file 1).

After successful execution, ACPYPE creates a folder that contains several files in different formats for the chosen MD programmes (see Figure 1). It can also fully replace the topology file converter from AMBER to GROMACS (**amb2gmx**[30,31]) with some notable differences: 

· In GROMACS, torsionals (proper and improper) are treated as Ryckaert-Bellemans potentials [32] and **amb2gmx** combines multiple AMBER torsions per quartet of atoms. ACPYPE in contrast separates improper from proper dihedrals, and, similarly to the ffAMBER project approach, uses the correct AMBER analytical function to treat proper dihedrals in GROMACS;

· ACPYPE does not depend on the **ambpdb** tool, which requires the AMBER proprietary package;

· ACPYPE reads and converts octahedron (INPCRD box) parameters to the GROMACS file. If not available, new box parameters will be calculated. It also recognises TIP3P or SPC/E water types and applies the correct parameters. This feature requires only the Python interpreter (see Figure 1) through the command: ‘acpype -p _prmtop_ -x _inpcrd_’.

### Testing - ACPYPE topologies versus AMBER force field

Since ACPYPE relies on ANTECHAMBER for generating topological parameters, it was possible to use a previously published validation procedure [17]. We generated 22 PDB files with PyMOL [33], each containing a tripeptide consisting of the same single natural amino, including protonation variants for His (for more details, see Additional file 2). GROMACS 4.5, which includes now ffAMBER, was then used to generate topology files for these tripeptides with the AMBER99SB force field as reference. In all cases a single point GROMACS energy minimisation step was performed.

### Testing - Small molecules from the PDB

ACPYPE (revision 275 with AmberTools 1.3) was executed on 8950 chemical components (ligands, small molecules and monomers) available from the PDB [34]. Two sets of files, one with the coordinates from the original PDB deposition and one with the ‘ideal’ CORINA coordinates [35,36] were written out in the MOL2 format via the CcpNmr FormatConverter [37] from the PDBe database [38,39], totalling 17900 input files. Charges were calculated using SQM with AM1-BC. The 17900 ACPYPE jobs, required a total execution time of just over 16 days on a computer using 20 AMD Opteron 2.3 GHz cores. The cut off time of execution per job was 10 hours, any job taking longer than that was killed.

### Testing - NMR structure calculation

We recalculated 5 protein-ligand NMR structures using the RECOORD protocol [40]. A purpose-written Python script that integrates the ACPYPE API with the CCPN API was developed to run ACPYPE on the ligand only to generate its GAFF force field parameters. These were incorporated into the standard protein topology files to calculate 200 initial structures by simulated annealing (SA) with CNS (topology and parameters from Engh & Huber [41] ). The 50 best of these structures were water refined (WR) using the OPLSX force field, with ACPYPE again providing the GAFF parameters for the ligand only (see Discussion). The 50 final structures were sorted by overall energy and the best 25 structures were validated through the iCING [42] server, and then compared against the validation of the original NMR structures as provided by NRG-CING [43]. Double the number of default RECOORD timesteps were used during the SA and WR because of the size of the proteins and presence of ligand.

## Results

We employed three tests to verify the correctness and applicability of the topologies generated by ACPYPE; to test its accuracy in transferring core data ACPYPE was compared to ffAMBER, to test its robustness ACPYPE was executed on a large set of small molecules from the PDB, and to test its usability ACPYPE-generated ligand topologies were employed to recalculate protein–ligand structures from NMR data.

### ACPYPE topologies versus ffAMBER

All atom types and parameters from GROMACS’ AMBER99SB output were identical to ACPYPE with the AMBER99SB option, with the following minor differences: 

· For histidine (all variants), arginine and tryptophan, ACPYPE generated some inverted improper dihedrals;

· For tryptophan ACPYPE incorporated 3 additional unnecessary (but harmless) improper dihedrals in the aromatic rings due to atom sharing;

· For the tyrosine CZ atom ACPYPE obtained atom type CA instead of C in GROMACS. This also results in parameter differences for 6 bonds and 9 dihedrals.

· The partial atom charges parameters differ.

Despite these changes, the difference in total bonded potential energy (*i.e.* without the long distance terms that depends on charges) for the 22 systems is very small between the ffAMBER and ACPYPE sets; the highest difference occurs for the tyrosine tripeptide and is 1.9% (6.7 kJ/mol). This is because of the the aforementioned atom type change and its consequent parameter modifications for bonds and dihedrals. For all other tripeptides, the difference is never higher than 0.002%. To further confirm that ACPYPE gives consistent results, we used the validation methodology by Eric Sorin and collaborators for ffAMBER [44] and compared the results from the AMBER11 MD engine (programme **sander**) to the results from GROMACS with ACPYPE topologies. For all systems except tyrosine, using the same set of charges as defined in the AMBER99SB force field, the total potential energy differences were always inferior to 0.007%. Tyrosine again was the outlier, but with a total potential energy difference <3%.

### Small molecules from the PDB

This test on 8950 small molecules served to evaluate the robustness of ACPYPE and debug the code. The first step was to curate the initial set of 8950 small molecule entries; since the information from the PDB is not always correct and the data went through a conversion process to generate the input files, entries with issues varying from total absence of input files to wrong atom coordinates were removed. Entries were also removed from further analysis if they did not adhere to a set of simple atom distance criteria (a 0.5 Å cut-off for minimum and a 3.0 Å cut-off for maximum distance between covalently bound atoms). From 17900 possible jobs (2 jobs for each PDB, one with original PDB coordinates and other with CORINA recalculated coordinates), 318 (1.78%) did not have MOL2 input files and could not be calculated, while 557 (3.11%) had erroneous atom coordinates. In total 13045 jobs (72.88%) concluded without any remarkable problems with an average execution time of 14m35s. Excluding the jobs with incorrect data the ACPYPE efficiency was 76.62% (13045 of 17025 valid jobs). For a detailed report, please see Additional file 3.

**Figure 2:**
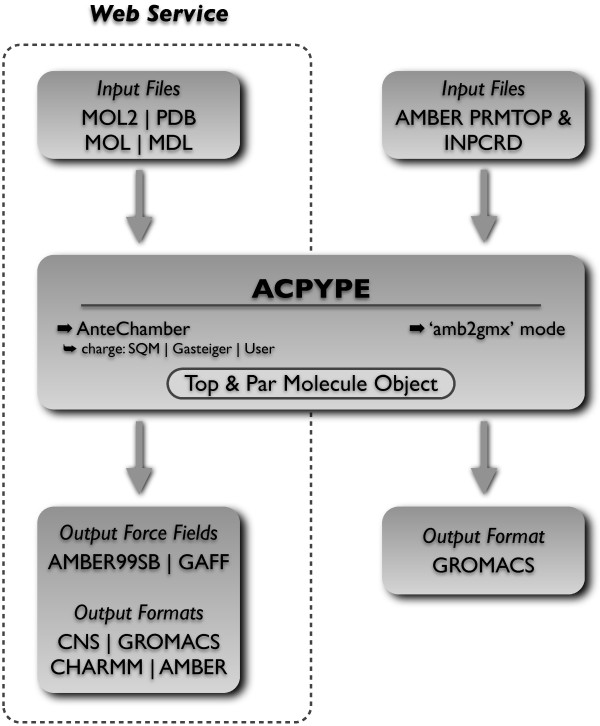
**RMSD distribution.** RMSD distribution for a total of 5126 entries (with two results each) after energy minimisation done with CNS programme. The average RMSD is shown by the bold vertical line.

To further explore whether the generated coordinates were correct or acceptable, we selected only entries with results from ACPYPE for both the PDB and CORINA coordinates. The resulting 5772 entries (11544 jobs) were subjected to 250 steps of energy minimisation via the conjugate gradient method using CNS (version 1.2). In total 1292 jobs failed the optimisation procedure because of mixed upper and lower case atom names, which CNS does not support. This occurred because ANTECHAMBER converts upper-case names to capitalised names (*e.g.*, bromine code ‘BR’ to ‘Br’); the issue was reported to ANTECHAMBER developers and is remedied in ACPYPE revision 285. For the remaining 10252 structures the all-atom RMSD between the initial and final structures was calculated (Figure 2) to illustrate the accuracy of the ACPYPE results.

### NMR structure calculation

**Figure 3:**
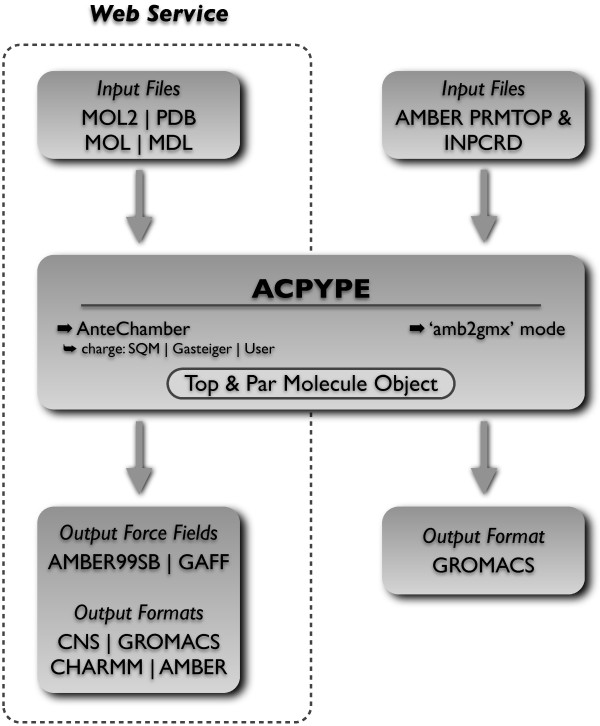
**Recalculated entry [PDB:1BVE] Entry [PDB:1BVE] from PDB recalculated using RECOORD protocol showing 25 models superimposed; picture created with VMD [**[45]**].**

To test how ACPYPE works in a real NMR structure calculation setting, we used the data for 5 protein–ligand complexes from the PDB (see Table 1) that have NMR constraint data in CCPN format from the NMR Restraints Grid [43]. The results of the structure calculation are similar; overall the RMSD tends to increase for the recalculated structures, but the NOE completeness and overall quality tends to increase (Table 1). These changes are expected due to differences in the structure calculation protocol, and are also observed in the RECOORD project [40]. More importantly, this test shows that ACPYPE allows the structure determination of protein–ligand complexes with autogenerated parameters and topologies (for illustrative purposes, the structures for [PDB:1BVE] are shown in Figure 3, the other structures are available in Additional file 4).

**Table 1:** Original NMR x ACPYPE

					
**Comparison data**	**[PDB:1BVE]**	**[PDB:1IKU]**	**[PDB:1JKN]**	**[PDB:2JN3]**	**[PDB:2K0G]**
**PDB ligand code**	DMP	MYR	ATP	JN3	CMP
**RMSD backbone (Å)**	1.16/1.16	1.52/2.53	0.97/1.56	0.47/1.55	2.24/2.30
**all atoms (Å)**	1.88/2.04	2.23/ 3.33	1.60/2.38	1.39/2.40	2.53/2.79
**Ramachandran core (%)**	71.9/88.5	79.9/79.4	82.4/84.8	84.9/72.9	90.6/87.1
**allowed (%)**	24.7/9.8	17.6/16.4	16.9/12.7	13.6/22.3	8.9/12.1
**generous (%)**	2.9/1.1	2.1/2.5	0.5/1.9	1.4/2.9	0.5/0.3
**disallowed (%)**	0.5/0.6	0.4/ 1.6	0.2/0.7	0.0/2.2	0.0/0.5
**NOE completeness (%)**	41.3/45.9	51.7/55.8	53.6/55.4	44.9/45.3	49.6/50.4
**CING ROG score green (%)**	25/15	78/133	56/83	74/75	52/70
**orange (%)**	29/25	51/43	53/49	28/36	47/48
**red (%)**	46/60	60/13	57/34	25/16	44/25

Structure quality indicator changes for the original NMR structures from the PDB (left of /) to the structures recalculated using the ACPYPE parameters for the ligand (right of /). RMSD values are the average pairwise between all structures in the ensemble.

## Discussion

The idea of adapting ANTECHAMBER or its routines to derive topologies and parameters for small molecules is not new. We know of at least two cases: YASARA AutoSMILES [29] is implemented for YASARA [46], but is restricted to this commercial software package; the programme **topolbuild** is developed by Bruce D. Ray (personal communication), and generates topologies and parameters from a MOL2 file (with known charges) by using AMBER, GROMOS [47] or OPLS/AA [13] force fields in GROMACS format. It is limited to GROMACS and not able to generate charges.

ACPYPE has the advantage that it avoids these limitations. Moreover, ACPYPE is written in Python and makes the (converted) information from ANTECHAMBER easily accessible for integration in other projects. In the NMR community, it is already availabel via CCPN [48], and it will be used in the upcoming rehash of the RECOORD structure recalculation project [40], where complexes will be included in addition to monomers. Pilot integration with ARIA2 [49], in order to make it work seamlessly via the CcpNmr Grid portal [50], was also tested. In the MD community, ACPYPE is used in the DrugDiscovery@Home project [51], and it is employed by others (for example, see [52]). We also intend to further verify ACPYPE based on virtualchemistry.org[53], a recent database of 145 organic molecules with some physical properties calculated and topologies for GAFF [17] and OPLS/AA validated by using the GROMACS software [54].

Since ACPYPE is based on ANTECHAMBER, it also inherits some of its core limitations: it is not possible to work with organic molecules with open valences; it cannot handle atoms besides C, N, O, S, P, H, F, Cl, Br and I; and there cannot be any covalent bonds to another (non-defined) molecule. Some of these restrictions can be circumvented: for example if one wants parameters for a modified amino acid residue, it is possible to neutralise the N- and C- termini and then fit the additional parameters manually to the modified residue.

The topological parameters generated by ACPYPE are based on GAFF or AMBER99SB and should be used only with compatible force fields such as AMBER and its variants; when employing ACPYPE to generate the ligand for a protein–ligand complex, the force field parameters for the protein should be from the AMBER family. However, it is possible to use CNS with topologies generated by ACPYPE, even if this means mixing two different force fields (Engh & Huber [41] and AMBER99SB/GAFF). This can be justified because during the SA steps of a structure calculation the values of all parameters are increased to much higher and fixed thresholds, and are so equalised for both protein and ligands. Essentially the topology information remains the same and all parameters are flattened (the GAFF or AMBER99SB parameters are overwritten by those from Engh & Huber), so the SA in CNS can be performed without problems, as illustrated by the protein-ligand case studies presented here. Likewise, during WR steps, the protein is described by OPLSX parameters (which are close to the original OPLS parameters and do not introduce new atom types), with identical topology description and very similar parameters to those used in the AMBER force field family.

Another point for consideration is the way improper dihedrals are defined in AMBER force fields. They are a set of “proper” dihedrals that act only in planes, which may result in chirality inversions or peptide bond flips during the high-temperature portions of SA runs. This problem is treated in AMBER MD applications by adding chirality constraints and *trans*-peptide *ω*constraints (where appropriate), but this solution is not easily extended to other MD programmes. However, since we use AMBER force fields only for small molecules, this is only a problem if the molecule has defined chiral centres. Where necessary it is possible to implement a routine to check the chiral centres every few steps using the CNS macro language, or to implement an extra step where the improper dihedrals are introduced in the ACPYPE generated topologies before the calculation. Although ACPYPE will work automatically in many cases, it is not recommended to use it as a “black box”, and one should always explore the molecule under investigation as well as the force field(s) used for parameterisation.

During the development of ACPYPE, some issues in AmberTools (with ANTECHAMBER in particular) were spotted, identified and reported back to their developers, sometimes with a proposed solution. This procedure only enriched the quality of both programmes, and emphasises the strength of working with open-source projects. Moreover, in relation to GROMACS, an open-source MD application, ACPYPE has great potential for usability and further development. ACPYPE is in constant development and has already a measurable community of users and contributors with ideas of extending it for other MD and NMR-SC programmes.

## Conclusions

ACPYPE is an ANTECHAMBER-based tool that fills the current gap in software to automatically incorporate small molecules in MD and NMR-SC. It calculates partial charges and generates topology and parameters in different formats for different MM programmes, while being object oriented for integration with other applications. It is a robust and flexible application, completely open source and freely available online for use by the scientific community.

## Availability and requirements

· **Project name:** ACPYPE - AnteChamber PYthon Parser interfacE

· **Home page:**http://www.ccpn.ac.uk/acpype

· **Operating Systems:** Platform independent

· **Programming language:** Python

· **Other requirements:** Python 2.6 or higher, including Python 3.x; Antechamber 1.27 or (preferably) AmberTools 1.0 or higher; (optional, but strongly recommended) Open Babel 2.2.0 or higher

· **License:** GNU GPL version 3

## Abbreviations

ACPYPE, AnteChamber PYthon Parser interfacE; GAFF, General Amber Force Field; MD, Molecular Mechanics; NMR-SC, Nuclear Magnetic Resonance Structure Calculation; SA, Simulated Annealing; WR, Water Refinement.

## Competing interests

Both authors declare that they do not have any competing interests.

## Authors’ contributions

AWSdS developed the software, carried out most of the testing and analysed the results. WFV provided input and test data, performed basic testing, and helped with the analysis of results. AWSdS wrote the manuscript with continuous support from WFV. Both authors read and approved the final manuscript version.

## Supplementary Material

Additional file 1 Other ways to generate charges for ACPYPE Link http://www.ccpn.ac.uk/software/ACPYPE-folder/user-charge-options. Click here for file

Additional file 2 A comparative test for ACPYPE Link http://www.ccpn.ac.uk/software/ACPYPE-folder/a-comparative-test-for-acpype. Click here for file

Additional file 3 Complete report for ACPYPE over 17900 ligands from PDB Link http://www.ccpn.ac.uk/software/ACPYPE-folder/results-for-ligands. Click here for file

Additional file 4 Figures for recalculated entries [PDB:1IKU], [PDB:1JKN], [PDB:2JN3] and [PDB:2K0G]. Click here for file
