# Supplementary material for: ACPYPE - AnteChamber PYthon Parser interfacE
Source: BMC Res Notes. 2012 Jul 23;5:367. doi: 10.1186/1756-0500-5-367 (PMC3461484; doi:10.1186/1756-0500-5-367)
Supplement: Additional file 1 — Other ways to generate charges for ACPYPE Link http://www.ccpn.ac.uk/software/ACPYPE-folder/user-charge-options. [file 1756-0500-5-367-S1.pdf]

User Charge Options

Other ways to generate charges for ACPYPE

If you are comfortable with AM1-BCC charges calculated via a semi-empirical method when using ACPYPE with default option, then you may consider one of the solutions below.

The best recommended method, despite being very time consuming, is R.E.D. (see also the service R.E.D. Server), which may use GAUSSIAN (commercial and quite expensive package) or GAMESS. This approach would closely reproduce the strict one used by AMBER force field developers.

An important note here. At first, it seems to be a weakness in ACPYPE/ANTECHAMBER approach, similar to the one referred to XPLO2D that a user need an initial set of coordinates for the molecule, but, regardless the conformation of the molecule, ACPYPE/ANTECHAMBER will get the topological parameters, however with a partial charge configuration depending on the input coordinates.

The rigorous recommended procedure above would be also able to address the problem of not having the initial coordinates of the small molecules by trying to optimise the molecule structure in a quantum mechanical level. Yet, this can be done with semi-empirical programmes like SQM itself or via conversion software like CORINA (commercial).

There is still a another way to obtain the partial charges and that would be to use YASARA AutoSMILES Server, where extra algorithms will try to refine the values calculated by a mopac-like programme. Save the file in PDB format and you find the charges stored in the occupancy field.

Once you have obtained the charges by one of the methods mentioned above, you can insert them in a MOL2 file to be submitted to ACPYPE using option `-c user`.
