# Supplementary material for: ACPYPE - AnteChamber PYthon Parser interfacE
Source: BMC Res Notes. 2012 Jul 23;5:367. doi: 10.1186/1756-0500-5-367 (PMC3461484; doi:10.1186/1756-0500-5-367)
Supplement: Additional file 2 — A comparative test for ACPYPE Link http://www.ccpn.ac.uk/software/ACPYPE-folder/a-comparative-test-for-acpype. [file 1756-0500-5-367-S2.pdf]

A Comparative Test for ACPYPE

Running a testing routine, I used `pymol` to generate 22 pdb files. They are tripeptides of every usual amino acid residue including 2 variants for HIS. All peptides have N- and T- termini.

I used GROMACS 4.5 with all these tripeptides to generate topology files with AMBER99SB forcefield as reference.

OBS: peptides JJJ.pdb (Hip-Hip-Hip) and RRR.pdb (Arg-Arg-Arg): their net charge should be +3, but *gasteiger* method (the way how ACPYPE try to guess the net charge of the molecule if not given) failed to get the right value, instead it says it is 'Zero', for which SQM programme will fail to calculate the atomic partial charges. So be aware that ACPYPE may guess a wrong net charge when running it on your own molecule.

Next, I used ACPYPE to generate the topologies and parameters for these 22 entries and compare theirs results with the ones from GROMACS reference.

By using ACPYPE with option '-a amber' (which means parm99.dat + gaff.dat + frcmod.ff99SB + frcmod.parmbsc0), it seems to give better results than using just GAFF (the default option in ACPYPE) when comparing with GROMACS outputs, i.e., the former basically got almost all atom types and parameters identical to the GROMACS with AMBER99SB reference, while the latter missed just a few more.

A more detailed comparison between ACPYPE with option '-a amber' against GROMACS' AMBER99SB (including a Single Point Energy minimisation with GROMACS) results and it can be seen that they **match** except by:

> *in terms of topology*

- entries HHH (Hie-Hie-Hie), JJJ (Hip-Hip-Hip), OOO (Hid-Hid-Hid), RRR (Arg-Arg-Arg) and WWW (Trp-Trp-Trp) have some improper dihedrals inverted;
- WWW which has 3 extra improper dihedrals related to atoms sharing in the 5-ring and 6-ring of the TRP. These improper dihedrals would be there in order to keep the planarity between 5-ring and 6-ring;
- YYY (Tyr-Tyr-Tyr) for which atom CZ (id 15, 36 and 57) got atom type CA instead of C in GROMACS.

> *in terms of parameters*

- charges (can be either *gasteiger* or *bcc* with ACPYPE);
- YYY: 6 bonds and 9 dihedrals, all involving atom CZ (id 15, 36 and 57), because of atom type changing mentioned above;

When we look the bonded potential energies for all entries, the difference is no bigger than 0.002% (even for the entries with inverted improper dihedrals) with one solely exception: YYY with 1.9%, because of the atom type change seen above.

The source code for these tests can be seen here.

See detailed output here

```
usePymol: True, ffType: amber, cType: gas

File AAA.pdb : residue ALA
Compare ACPYPE x GMX AMBER99SB topol & param
    ==> Comparing atomtypes AC x AMBER
        atomtypes OK
    ==> Comparing pairs
        pairs OK
    ==> Comparing bonds
        bonds OK
    ==> Comparing angles
        angles OK
    ==> Comparing dihedrals
        dihedrals OK
Compare ACPYPE x GMX AMBER99SB Pot. Energy (|ERROR|%)
          LJ-14   0.000
        LJ_(SR)   0.000
     Coulomb-14   76.981   215.415000 x 935.821000
   Coulomb_(SR)   62.786   -387.017000 x -1039.970000
 TotalNonBonded   42.492   -158.744190 x -91.291190
      Potential   63.728   38.393100 x 105.849000
          Angle   0.000
           Bond   0.000
    Proper_Dih.   0.000
  Improper_Dih.   0.000
   Dihedral P+I   0.000
   Total_Bonded   0.001

File CCC.pdb : residue CYS
Compare ACPYPE x GMX AMBER99SB topol & param
    ==> Comparing atomtypes AC x AMBER
        atomtypes OK
    ==> Comparing pairs
        pairs OK
    ==> Comparing bonds
        bonds OK
    ==> Comparing angles
        angles OK
    ==> Comparing dihedrals
        dihedrals OK
Compare ACPYPE x GMX AMBER99SB Pot. Energy (|ERROR|%)
          LJ-14   0.000
        LJ_(SR)   0.000
     Coulomb-14   76.949   188.291000 x 816.834000
   Coulomb_(SR)   59.574   -342.955000 x -848.355000
 TotalNonBonded   80.193   -153.559000 x -30.416000
      Potential   72.553   46.585300 x 169.729000
          Angle   0.000
           Bond   0.000
    Proper_Dih.   0.000
  Improper_Dih.   0.000
   Dihedral P+I   0.000
   Total_Bonded   0.000

File DDD.pdb : residue ASP
Compare ACPYPE x GMX AMBER99SB topol & param
    ==> Comparing atomtypes AC x AMBER
        atomtypes OK
    ==> Comparing pairs
```

```
        pairs OK
    ==> Comparing bonds
        bonds OK
    ==> Comparing angles
        angles OK
    ==> Comparing dihedrals
        dihedrals OK
Compare ACPYPE x GMX AMBER99SB Pot. Energy (|ERROR|%)
          LJ-14   0.000
        LJ_(SR)   0.000
     Coulomb-14   98.557   20.689000 x 1434.240000
   Coulomb_(SR)   152.312   413.830000 x -791.079000
 TotalNonBonded   32.568   432.001700 x 640.643700
      Potential   24.480   643.643000 x 852.282000
          Angle   0.000
           Bond   0.000
    Proper_Dih.   0.000
  Improper_Dih.   0.000
   Dihedral P+I   0.000
   Total_Bonded   0.001

File EEE.pdb : residue GLU
Compare ACPYPE x GMX AMBER99SB topol & param
    ==> Comparing atomtypes AC x AMBER
        atomtypes OK
    ==> Comparing pairs
        pairs OK
    ==> Comparing bonds
        bonds OK
    ==> Comparing angles
        angles OK
    ==> Comparing dihedrals
        dihedrals OK
Compare ACPYPE x GMX AMBER99SB Pot. Energy (|ERROR|%)
          LJ-14   0.000
        LJ_(SR)   0.000
     Coulomb-14   84.382   146.496000 x 937.969000
   Coulomb_(SR)   113.598   54.962600 x -404.192000
 TotalNonBonded   61.892   204.617700 x 536.936100
      Potential   42.278   453.717000 x 786.037000
          Angle   0.000
           Bond   0.000
    Proper_Dih.   0.000
  Improper_Dih.   0.000
   Dihedral P+I   0.000
   Total_Bonded   0.001

File FFF.pdb : residue PHE
Compare ACPYPE x GMX AMBER99SB topol & param
    ==> Comparing atomtypes AC x AMBER
        atomtypes OK
    ==> Comparing pairs
        pairs OK
    ==> Comparing bonds
        bonds OK
    ==> Comparing angles
        angles OK
    ==> Comparing dihedrals
        dihedrals OK
Compare ACPYPE x GMX AMBER99SB Pot. Energy (|ERROR|%)
```

```
          LJ-14   0.000
        LJ_(SR)   0.000
     Coulomb-14   78.357   182.820000 x 844.711000
   Coulomb_(SR)   63.253   -377.329000 x -1026.840000
 TotalNonBonded   7.267   -170.350100 x -157.970100
      Potential   16.913   60.805000 x 73.182000
          Angle   0.000
           Bond   0.000
    Proper_Dih.   0.000
  Improper_Dih.   0.000
   Dihedral P+I   0.000
   Total_Bonded   0.001

File GGG.pdb : residue GLY
Compare ACPYPE x GMX AMBER99SB topol & param
    ==> Comparing atomtypes AC x AMBER
        atomtypes OK
    ==> Comparing pairs
        pairs OK
    ==> Comparing bonds
        bonds OK
    ==> Comparing angles
        angles OK
    ==> Comparing dihedrals
        dihedrals OK
Compare ACPYPE x GMX AMBER99SB Pot. Energy (|ERROR|%)
          LJ-14   0.000
        LJ_(SR)   0.000
     Coulomb-14   73.451   173.346000 x 652.926000
   Coulomb_(SR)   61.248   -357.586000 x -922.746000
 TotalNonBonded   34.100   -165.389450 x -250.969450
      Potential   58.507   146.272000 x 60.692400
          Angle   0.000
           Bond   0.000
    Proper_Dih.   0.000
  Improper_Dih.   0.000
   Dihedral P+I   0.000
   Total_Bonded   0.000

File HHH.pdb : residue HIE
Compare ACPYPE x GMX AMBER99SB topol & param
    ==> Comparing atomtypes AC x AMBER
        atomtypes OK
    ==> Comparing pairs
        pairs OK
    ==> Comparing bonds
        bonds OK
    ==> Comparing angles
        angles OK
    ==> Comparing dihedrals
        dihedrals OK
Compare ACPYPE x GMX AMBER99SB Pot. Energy (|ERROR|%)
          LJ-14   0.000
        LJ_(SR)   0.000
     Coulomb-14   81.888   173.302000 x 956.861000
   Coulomb_(SR)   69.494   -348.907000 x -1143.720000
 TotalNonBonded   5.514   -192.836000 x -204.090000
      Potential   3.084   365.312000 x 354.047000
          Angle   0.000
           Bond   0.001
```

```
    Proper_Dih.   0.000
  Improper_Dih.   1.532   0.898273 x 0.884509
   Dihedral P+I   0.014
   Total_Bonded   0.002
('IDIH: Amb diff Acp', ['10 13 11 12', '23 26 32 27', '26 30 32 33', '27
30 28 29', '40 43 49 44', '43 47 49 50', '44 47 45 46', '6 9 15 10', '9
13 15 16'])
('IDIH: Acp diff Amb', ['12 13 11 10', '23 32 26 27', '26 33 32 30', '29
30 28 27', '40 49 43 44', '43 50 49 47', '46 47 45 44', '6 15 9 10', '9
16 15 13'])

File III.pdb : residue ILE
Compare ACPYPE x GMX AMBER99SB topol & param
    ==> Comparing atomtypes AC x AMBER
        atomtypes OK
    ==> Comparing pairs
        pairs OK
    ==> Comparing bonds
        bonds OK
    ==> Comparing angles
        angles OK
    ==> Comparing dihedrals
        dihedrals OK
Compare ACPYPE x GMX AMBER99SB Pot. Energy (|ERROR|%)
          LJ-14   0.000
        LJ_(SR)   0.000
     Coulomb-14   69.230   199.075000 x 646.988000
   Coulomb_(SR)   58.412   -354.812000 x -853.159000
 TotalNonBonded   24.390   -156.344000 x -206.778000
      Potential   35.203   143.271000 x 92.835900
          Angle   0.000
           Bond   0.000
    Proper_Dih.   0.000
  Improper_Dih.   0.000
   Dihedral P+I   0.000
   Total_Bonded   0.000

File JJJ.pdb : residue HIP
Compare ACPYPE x GMX AMBER99SB topol & param
    ==> Comparing atomtypes AC x AMBER
        atomtypes OK
    ==> Comparing pairs
        pairs OK
    ==> Comparing bonds
        bonds OK
    ==> Comparing angles
        angles OK
    ==> Comparing dihedrals
        dihedrals OK
Compare ACPYPE x GMX AMBER99SB Pot. Energy (|ERROR|%)
          LJ-14   0.000
        LJ_(SR)   0.000
     Coulomb-14   90.527   101.523000 x 1071.690000
   Coulomb_(SR)   69.386   -309.479000 x -94.742400
 TotalNonBonded   123.848   -228.163800 x 956.739800
      Potential   73.287   431.906000 x 1616.810000
          Angle   0.000
           Bond   0.000
    Proper_Dih.   0.000
  Improper_Dih.   0.461   1.165150 x 1.159780
```

```
   Dihedral P+I   0.006
   Total_Bonded   0.000
('IDIH: Amb diff Acp', ['10 14 12 13', '24 27 34 28', '27 32 34 35', '28
32 30 31', '42 45 52 46', '45 50 52 53', '46 50 48 49', '6 9 16 10', '9
14 16 17'])
('IDIH: Acp diff Amb', ['13 10 12 14', '24 34 27 28', '27 35 34 32', '31
28 30 32', '42 52 45 46', '45 53 52 50', '49 46 48 50', '6 16 9 10', '9
17 16 14'])

File KKK.pdb : residue LYS
Compare ACPYPE x GMX AMBER99SB topol & param
    ==> Comparing atomtypes AC x AMBER
        atomtypes OK
    ==> Comparing pairs
        pairs OK
    ==> Comparing bonds
        bonds OK
    ==> Comparing angles
        angles OK
    ==> Comparing dihedrals
        dihedrals OK
Compare ACPYPE x GMX AMBER99SB Pot. Energy (|ERROR|%)
          LJ-14   0.000
        LJ_(SR)   0.000
     Coulomb-14   74.108   339.525000 x 1311.310000
   Coulomb_(SR)   103.779   23.481500 x -621.413000
 TotalNonBonded   47.224   365.320900 x 692.211400
      Potential   34.840   611.384000 x 938.277000
          Angle   0.000
           Bond   0.000
    Proper_Dih.   0.000
  Improper_Dih.   0.000
   Dihedral P+I   0.000
   Total_Bonded   0.001

File LLL.pdb : residue LEU
Compare ACPYPE x GMX AMBER99SB topol & param
    ==> Comparing atomtypes AC x AMBER
        atomtypes OK
    ==> Comparing pairs
        pairs OK
    ==> Comparing bonds
        bonds OK
    ==> Comparing angles
        angles OK
    ==> Comparing dihedrals
        dihedrals OK
Compare ACPYPE x GMX AMBER99SB Pot. Energy (|ERROR|%)
          LJ-14   0.000
        LJ_(SR)   0.000
     Coulomb-14   74.848   186.743000 x 742.452000
   Coulomb_(SR)   69.131   -349.887000 x -1133.470000
 TotalNonBonded   58.805   -159.631700 x -387.505700
      Potential   173.384   96.444700 x -131.425000
          Angle   0.000
           Bond   0.000
    Proper_Dih.   0.000
  Improper_Dih.   0.000
   Dihedral P+I   0.000
   Total_Bonded   0.002
```

```
File MMM.pdb : residue MET
Compare ACPYPE x GMX AMBER99SB topol & param
    ==> Comparing atomtypes AC x AMBER
        atomtypes OK
    ==> Comparing pairs
        pairs OK
    ==> Comparing bonds
        bonds OK
    ==> Comparing angles
        angles OK
    ==> Comparing dihedrals
        dihedrals OK
Compare ACPYPE x GMX AMBER99SB Pot. Energy (|ERROR|%)
          LJ-14   0.000
        LJ_(SR)   0.000
     Coulomb-14   76.769   198.587000 x 854.851000
   Coulomb_(SR)   65.492   -351.183000 x -1017.690000
 TotalNonBonded   6.061   -158.745400 x -168.988400
      Potential   15.269   67.102100 x 56.856000
          Angle   0.000
           Bond   0.001
    Proper_Dih.   0.000
  Improper_Dih.   0.000
   Dihedral P+I   0.000
   Total_Bonded   0.001

File NNN.pdb : residue ASN
Compare ACPYPE x GMX AMBER99SB topol & param
    ==> Comparing atomtypes AC x AMBER
        atomtypes OK
    ==> Comparing pairs
        pairs OK
    ==> Comparing bonds
        bonds OK
    ==> Comparing angles
        angles OK
    ==> Comparing dihedrals
        dihedrals OK
Compare ACPYPE x GMX AMBER99SB Pot. Energy (|ERROR|%)
          LJ-14   0.000
        LJ_(SR)   0.000
     Coulomb-14   91.472   23.449900 x 274.966000
   Coulomb_(SR)   70.875   -405.220000 x -1391.320000
 TotalNonBonded   65.275   -390.779400 x -1125.363300
      Potential   93.657   -49.749900 x -784.336000
          Angle   0.001
           Bond   0.000
    Proper_Dih.   0.000
  Improper_Dih.   0.000
   Dihedral P+I   0.000
   Total_Bonded   0.001

File OOO.pdb : residue HID
Compare ACPYPE x GMX AMBER99SB topol & param
    ==> Comparing atomtypes AC x AMBER
        atomtypes OK
    ==> Comparing pairs
        pairs OK
    ==> Comparing bonds
```

```
        bonds OK
    ==> Comparing angles
        angles OK
    ==> Comparing dihedrals
        dihedrals OK
Compare ACPYPE x GMX AMBER99SB Pot. Energy (|ERROR|%)
          LJ-14   0.000
        LJ_(SR)   0.000
     Coulomb-14   79.204   141.251000 x 679.208000
   Coulomb_(SR)   67.748   -314.460000 x -975.015000
 TotalNonBonded   40.869   -177.377900 x -299.975900
      Potential   24.679   496.783000 x 374.182000
          Angle   0.000
           Bond   0.000
    Proper_Dih.   0.000
  Improper_Dih.   0.209   1.048090 x 1.045900
   Dihedral P+I   0.002
   Total_Bonded   0.000
('IDIH: Amb diff Acp', ['10 14 12 13', '23 26 32 27', '26 31 32 33', '27
31 29 30', '40 43 49 44', '43 48 49 50', '44 48 46 47', '6 9 15 10', '9
14 15 16'])
('IDIH: Acp diff Amb', ['13 10 12 14', '23 32 26 27', '26 33 32 31', '30
27 29 31', '40 49 43 44', '43 50 49 48', '47 44 46 48', '6 15 9 10', '9
16 15 14'])

File PPP.pdb : residue PRO
Compare ACPYPE x GMX AMBER99SB topol & param
    ==> Comparing atomtypes AC x AMBER
        atomtypes OK
    ==> Comparing pairs
        pairs OK
    ==> Comparing bonds
        bonds OK
    ==> Comparing angles
        angles OK
    ==> Comparing dihedrals
        dihedrals OK
Compare ACPYPE x GMX AMBER99SB Pot. Energy (|ERROR|%)
          LJ-14   0.000
        LJ_(SR)   0.000
     Coulomb-14   73.364   218.006000 x 818.454000
   Coulomb_(SR)   55.847   -313.270000 x -709.513000
 TotalNonBonded   172.715   -85.972800 x 118.232200
      Potential   30.996   454.618000 x 658.824000
          Angle   0.000
           Bond   0.000
    Proper_Dih.   0.000
  Improper_Dih.   0.000
   Dihedral P+I   0.000
   Total_Bonded   0.000

File QQQ.pdb : residue GLN
Compare ACPYPE x GMX AMBER99SB topol & param
    ==> Comparing atomtypes AC x AMBER
        atomtypes OK
    ==> Comparing pairs
        pairs OK
    ==> Comparing bonds
        bonds OK
    ==> Comparing angles
```

```
        angles OK
    ==> Comparing dihedrals
        dihedrals OK
Compare ACPYPE x GMX AMBER99SB Pot. Energy (|ERROR|%)
          LJ-14   0.000
        LJ_(SR)   0.000
     Coulomb-14   184.660   104.048000 x -88.087400
   Coulomb_(SR)   53.699   -396.819000 x -857.046000
 TotalNonBonded   69.174   -290.715400 x -943.077800
      Potential   123.577   124.461000 x -527.902000
          Angle   0.000
           Bond   0.001
    Proper_Dih.   0.000
  Improper_Dih.   0.000
   Dihedral P+I   0.000
   Total_Bonded   0.000

File RRR.pdb : residue ARG
Compare ACPYPE x GMX AMBER99SB topol & param
    ==> Comparing atomtypes AC x AMBER
        atomtypes OK
    ==> Comparing pairs
        pairs OK
    ==> Comparing bonds
        bonds OK
    ==> Comparing angles
        angles OK
    ==> Comparing dihedrals
        dihedrals OK
Compare ACPYPE x GMX AMBER99SB Pot. Energy (|ERROR|%)
          LJ-14   0.000
        LJ_(SR)   0.000
     Coulomb-14   82.729   -504.705000 x -2922.250000
   Coulomb_(SR)   44.608   772.922000 x 1395.370000
 TotalNonBonded   117.425   266.374400 x -1528.722600
      Potential   145.008   557.173000 x -1237.930000
          Angle   0.000
           Bond   0.001
    Proper_Dih.   0.000
  Improper_Dih.   1.900   0.359230 x 0.352406
   Dihedral P+I   0.012
   Total_Bonded   0.002
('IDIH: Amb diff Acp', ['12 17 15 16', '36 41 39 40', '60 65 63 64'])
('IDIH: Acp diff Amb', ['17 12 15 16', '41 36 39 40', '65 60 63 64'])

File SSS.pdb : residue SER
Compare ACPYPE x GMX AMBER99SB topol & param
    ==> Comparing atomtypes AC x AMBER
        atomtypes OK
    ==> Comparing pairs
        pairs OK
    ==> Comparing bonds
        bonds OK
    ==> Comparing angles
        angles OK
    ==> Comparing dihedrals
        dihedrals OK
Compare ACPYPE x GMX AMBER99SB Pot. Energy (|ERROR|%)
          LJ-14   0.000
        LJ_(SR)   0.000
```

```
     Coulomb-14   49.075   193.833000 x 380.621000
   Coulomb_(SR)   51.509   -278.751000 x -574.856000
 TotalNonBonded   58.465   -77.661300 x -186.978300
      Potential   88.782   123.129000 x 13.812000
          Angle   0.000
           Bond   0.001
    Proper_Dih.   0.000
  Improper_Dih.   0.000
   Dihedral P+I   0.000
   Total_Bonded   0.000

File TTT.pdb : residue THR
Compare ACPYPE x GMX AMBER99SB topol & param
    ==> Comparing atomtypes AC x AMBER
        atomtypes OK
    ==> Comparing pairs
        pairs OK
    ==> Comparing bonds
        bonds OK
    ==> Comparing angles
        angles OK
    ==> Comparing dihedrals
        dihedrals OK
Compare ACPYPE x GMX AMBER99SB Pot. Energy (|ERROR|%)
          LJ-14   0.000
        LJ_(SR)   0.000
     Coulomb-14   166.024   135.605000 x -205.387000
   Coulomb_(SR)   29.208   -339.284000 x -479.267000
 TotalNonBonded   70.431   -201.923900 x -682.898900
      Potential   105.054   23.138000 x -457.837000
          Angle   0.000
           Bond   0.001
    Proper_Dih.   0.000
  Improper_Dih.   0.000
   Dihedral P+I   0.000
   Total_Bonded   0.000

File VVV.pdb : residue VAL
Compare ACPYPE x GMX AMBER99SB topol & param
    ==> Comparing atomtypes AC x AMBER
        atomtypes OK
    ==> Comparing pairs
        pairs OK
    ==> Comparing bonds
        bonds OK
    ==> Comparing angles
        angles OK
    ==> Comparing dihedrals
        dihedrals OK
Compare ACPYPE x GMX AMBER99SB Pot. Energy (|ERROR|%)
          LJ-14   0.000
        LJ_(SR)   0.000
     Coulomb-14   6.013   195.043000 x 207.522000
   Coulomb_(SR)   46.468   -353.881000 x -661.068000
 TotalNonBonded   65.080   -158.129200 x -452.837200
      Potential   142.174   87.421000 x -207.287000
          Angle   0.000
           Bond   0.000
    Proper_Dih.   0.000
  Improper_Dih.   0.000
```

```
   Dihedral P+I   0.000
   Total_Bonded   0.000

File WWW.pdb : residue TRP
Compare ACPYPE x GMX AMBER99SB topol & param
    ==> Comparing atomtypes AC x AMBER
        atomtypes OK
    ==> Comparing pairs
        pairs OK
    ==> Comparing bonds
        bonds OK
    ==> Comparing angles
        angles OK
    ==> Comparing dihedrals
('    ac: ', [[16, 24, 15, 13, 4, 180.0, 4.602, 2.0], [40, 48, 39, 37, 4,
180.0, 4.602, 2.0], [64, 72, 63, 61, 4, 180.0, 4.602, 2.0]])
Compare ACPYPE x GMX AMBER99SB Pot. Energy (|ERROR|%)
          LJ-14   0.000
        LJ_(SR)   0.000
     Coulomb-14   77.500   227.454000 x 1010.900000
   Coulomb_(SR)   60.345   -430.220000 x -1084.920000
 TotalNonBonded   61.207   -210.345000 x -81.599000
      Potential   52.058   118.566000 x 247.309000
          Angle   0.000
           Bond   0.000
    Proper_Dih.   0.000
  Improper_Dih.   0.598   0.190516 x 0.189376
   Dihedral P+I   0.002
   Total_Bonded   0.001
('IDIH: Amb diff Acp', ['10 14 12 13', '14 17 15 16', '33 36 34 35', '34
38 36 37', '38 41 39 40', '57 60 58 59', '58 62 60 61', '62 65 63 64', '9
12 10 11'])
('IDIH: Acp diff Amb', ['14 10 12 13', '15 23 14 12', '17 14 15 16', '33
35 34 36', '38 34 36 37', '39 47 38 36', '41 38 39 40', '57 59 58 60',
'62 58 60 61', '63 71 62 60', '65 62 63 64', '9 11 10 12'])

File YYY.pdb : residue TYR
Compare ACPYPE x GMX AMBER99SB topol & param
    ==> Comparing atomtypes AC x AMBER
    15 CZ AC: CA   x   AMB: C
    36 CZ AC: CA   x   AMB: C
    57 CZ AC: CA   x   AMB: C
    ==> Comparing pairs
        pairs OK
    ==> Comparing bonds
('    ac: ', [[13, 15, 1, 0.14, 392460.0], [13, 15, 1, 0.141, 392460.0],
[15, 18, 1, 0.14, 392460.0], [15, 18, 1, 0.141, 392460.0], [34, 36, 1,
0.14, 392460.0], [34, 36, 1, 0.141, 392460.0], [36, 39, 1, 0.14,
392460.0], [36, 39, 1, 0.141, 392460.0], [55, 57, 1, 0.14, 392460.0],
[55, 57, 1, 0.141, 392460.0], [57, 60, 1, 0.14, 392460.0], [57, 60, 1,
0.141, 392460.0]])
    ==> Comparing angles
        angles OK
    ==> Comparing dihedrals
('    ac: ', [[13, 15, 16, 17, 9, 180.0, 9.623, 2.0], [17, 16, 15, 18, 9,
180.0, 3.766, 2.0], [18, 15, 16, 17, 9, 180.0, 9.623, 2.0], [34, 36, 37,
38, 9, 180.0, 9.623, 2.0], [38, 37, 36, 39, 9, 180.0, 3.766, 2.0], [39,
36, 37, 38, 9, 180.0, 9.623, 2.0], [55, 57, 58, 59, 9, 180.0, 9.623,
2.0], [59, 58, 57, 60, 9, 180.0, 3.766, 2.0], [60, 57, 58, 59, 9, 180.0,
9.623, 2.0]])
```

```
Compare ACPYPE x GMX AMBER99SB Pot. Energy (|ERROR|%)
          LJ-14   0.000
        LJ_(SR)   0.000
     Coulomb-14   78.850   146.778000 x 693.988000
   Coulomb_(SR)   66.753   -367.937000 x -1106.690000
 TotalNonBonded   49.296   -197.017700 x -388.560700
      Potential   121.680   151.870000 x -32.925800
          Angle   0.000
           Bond   0.967   215.247000 x 217.349000
    Proper_Dih.   7.765   55.205700 x 59.853100
  Improper_Dih.   0.000
   Dihedral P+I   7.749   55.323112 x 59.970512
   Total_Bonded   1.897   348.887700 x 355.634900
```
