# Supplementary material for: ACPYPE - AnteChamber PYthon Parser interfacE
Source: BMC Res Notes. 2012 Jul 23;5:367. doi: 10.1186/1756-0500-5-367 (PMC3461484; doi:10.1186/1756-0500-5-367)
Supplement: Additional file 3 — Complete report for ACPYPE over 17900 ligands from PDB Link http://www.ccpn.ac.uk/software/ACPYPE-folder/results-for-ligands. [file 1756-0500-5-367-S3.pdf]

Results for Ligands

Complete detailed summarised results for ACPYPE over 17900 ligands from PDB

The complete file with "Detailed atom report per entry" is here

```
& Symbol '&' (ampersand) means lines inserted manually in the
automatic generated report

& It was 8950 entries (ccpCodes), each possibly containing 2 mol2
entries, one from the PDB and another a IDEAL structure generated by
Corina. The max total of jobs would be 17900 and they took a combined
total time of execution of 2w 2d 7h 25m 14s in a computer
(mammoth.bio.cam.ac.uk) with 32 cores, but using only 20 cores at a
time.

& The cut off time of execution per job was 10h, i.e., any job lasting
more than 10h would be killed.

& Cut-off for minimum distance between atoms: 0.5 Ang.
& Cut-off for max distance between atoms: 3.0 Ang.

& It was used ACPYPE rev. 275 with AmberTools 1.3 using SQM instead of
MOPAC for am1 bcc charges calculation. SQM here was modified to
include 6 decimals of precision instead of the default 3.

& From a total of 17900 jobs possible 13045 (72.88%) concluded without
any remarkable note. Nevertheless it doesn't mean they are corrected
of even acceptable.

& Total of missing input data and wrong coordinates: 318 + 107 + 96 +
68 + 9 + 7 + 4 + 28 + 219 + 16 + 3 = 875 jobs

& ACPYPE efficiency considering only valid jobs (17900 - 875 = 17025
valid jobs): 76.62% (13045 of 17025 jobs)

& >>> Time Job Execution Summary <<<

& Number of clean jobs: 13045
& Longest job: Mol='Rad_pdb', time= 9h 59m 46s
& Fatest job: Mol='Mmc_ideal', time= 0s
& Average time of execution per clean job: 14m 35s

& Total number of jobs: 17582 (318 simply didn't have the *.mol2 input
file, see below)
& Global average time of execution per job: 26m 17s

&&&&& body of results with comments &&&&&

& Results are grouped in 31 types, each type containing 3 divisions
(BOTH, PDB, IDEAL)

& This error below is indeed known and it's a problem with SQM in
Linux. This entry IUM has a Uranium atom and SQM should abort but
instead it proceeded.
```

```
UNKNOWN ERROR: /home/awd28/other/Ium/Ium.none_neutral.pdb.out ERROR:
The unperturbed charge of the unit: 10.815713 is not integral.

*2 E, 3 W, ET _0_10, WT _0_3_4*          0 1 0
----------------------------------------------------------------------
----------

*** For results [1], [2], [3], dirs totally empty or at least missing
one *.mol2 input files for either PDB or IDEAL):

[1] Dirs missing *.mol2 input files for both PDB and Ideal:
22     ['0e3', '0e4', '0ez', '10f', '16u', '23u', '2he', '3dr',
'A01_dna', 'A01_rna', 'C01_dna', 'C01_rna', 'C43', 'G01_dna',
'G01_rna', 'H', 'I01_dna', 'I01_rna', 'T01_dna', 'T01_rna', 'U01_dna',
'U01_rna']

[2] Dirs missing *.mol2 input files with PDB ONLY, besides [1]:
24     ['031', '084', '098', '099', '102', '12h', '1bu', '20a', '2mb',
'Afg', 'Chm', 'Cig', 'Dop', 'Eom', 'Fou', 'Glq', 'Hts', 'Ilt', 'Meo',
'Nin', 'Pe1', 'Tat', 'Tnp', 'Toe']

[3] Dirs missing *.mol2 input files with IDEAL ONLY, besides [1]:
250     ['006', '0a5', '118', '182', '1fh', '1pt', '23d', '23s',
'2db', '2kl', '2pt', '2st', '2xy', '32s', '3ft', '3on', '543', '6he',
'749', '7he', '803', 'A71', 'A72', 'Ac9', 'Ad6', 'Aio', 'Alf', 'Alk',
'Art', 'Ast', 'Asx', 'Atc', 'Ats', 'Avc', 'Axt', 'Aze', 'B13', 'Baz',
'Bbr', 'Bcb', 'Bcr', 'Bef', 'Bf4', 'Blt', 'Bpb', 'Bpp', 'Bpt', 'Bva',
'C7p', 'Cac', 'Cbe', 'Cch', 'Cd1', 'Cd3', 'Cd5', 'Ceh', 'Ces', 'Cfc',
'Cfm', 'Chl', 'Cl1', 'Cla', 'Clf', 'Cln', 'Coh', 'Cpo', 'Cpt', 'Csl',
'Ctc', 'Cub', 'Cum', 'Cun', 'Cup', 'Cus', 'Cuz', 'Dae', 'Daq', 'Ddh',
'Def', 'Dhe', 'Dhq', 'Dlq', 'Dos', 'Dvt', 'Eaa', 'Eb1', 'Emc', 'Etr',
'Eys', 'Fc6', 'Fci', 'Fdc', 'Fdd', 'Fde', 'Fec', 'Fem', 'Fen', 'Fll',
'Fne', 'Fs1', 'Fso', 'Fsx', 'Fxm', 'Ger', 'Glx', 'Gms', 'Has', 'Hb1',
'Hc0', 'Hc1', 'Hcn', 'He5', 'He6', 'Hea', 'Heb', 'Hec', 'Heg', 'Hem',
'Hen', 'Heo', 'Heq', 'Hes', 'Hev', 'Hey', 'Hf5', 'Hfm', 'Hgt', 'Hif',
'Hkl', 'Hme', 'Hni', 'I42', 'Ica', 'Imf', 'Inr', 'Iri', 'Jm1', 'Keg',
'Kir', 'L0c', 'L0e', 'Los', 'Lpt', 'Lqq', 'Lut', 'Lyc', 'Mae', 'Mau',
'Mbv', 'Mc9', 'Mci', 'Md1', 'Me3', 'Mez', 'Mh3', 'Miy', 'Mm5', 'Mm6',
'Mn5', 'Mn6', 'Mnh', 'Mnq', 'Mnr', 'Mo7', 'Mom', 'Moo', 'Mp1', 'Msf',
'Mss', 'Mtc', 'Mtd', 'Mvd', 'Mw2', 'Mw3', 'Myq', 'Na2', 'Na5', 'Na6',
'Nao', 'Naw', 'Nd4', 'Nfs', 'Ngv', 'Nod', 'O4m', 'Oc2', 'Oc3', 'Oc4',
'Oc5', 'Oc6', 'Oc7', 'Oc8', 'Oec', 'Omo', 'Onp', 'Oxr', 'Pbm', 'Pc3',
'Pc4', 'Pcd', 'Pfc', 'Ph7', 'Phf', 'Pid', 'Pnq', 'Poh', 'Pp9', 'Pqq',
'Ptn', 'Px1', 'R1c', 'Rea', 'Req', 'Ret', 'Rfp', 'Rhx', 'Rpt', 'Rta',
'Rtb', 'Rtl', 'Ru7', 'Se4', 'Sm4', 'Sng', 'Spw', 'Srm', 'Sse', 'Swi',
'T1a', 'T4s', 'Ta3', 'Tac', 'Til', 'Tnc', 'Tne', 'Tpt', 'Tsd', 'Tta',
'Tth', 'Ttx', 'Ums', 'Unx', 'Va1', 'Ver', 'Wo2', 'Xug', 'Ybt', 'Zem',
'Zex', 'Znh']
PDB Total: 46 of 17900 (0.26%)
IDEAL Total: 272 of 17900 (1.52%)
Total: 318 of 17900 (1.78%)
----------------------------------------------------------------------
----------
----------------------------------------------------------------------
----------

*** For results [4], [5], [6], mols clean, no erros or warnings:

[4] Mols clean for both PDB and Ideal:
```

5772 ['002', '003', '007', '008', '017', '01g', '01k', '024',
'028', '033', '039', '041', '042', '047', '055', '057', '059', '062',
'064', '065', '072', '073', '074', '075', '088', '093', '094', '097',
'0a3', '0a4', '0a6', '0a7', '0ai', '0aj', '0al', '0am', '0ao', '0g6',
'0ma', '0mo', '0pa', '0pn', '0z6', '0z9', '100', '101', '103', '104',
'106', '107', '108', '109', '10a', '10m', '110', '111', '112', '113',
'114', '115', '116', '117', '11b', '11m', '11n', '11o', '11p', '11s',
'11u', '11x', '125', '126', '129', '12a', '12b', '12m', '12p', '12q',
'133', '134', '135', '136', '137', '138', '13d', '13p', '13r', '13s',
'13u', '140', '141', '142', '144', '145', '146', '147', '149', '14a',
'14c', '14o', '150', '151', '155', '156', '157', '158', '159', '15a',
'15b', '15m', '160', '161', '162', '164', '165', '167', '168', '169',
'16a', '16c', '16d', '16g', '16p', '170', '171', '172', '174', '176',
'177', '178', '179', '17a', '17b', '17h', '17m', '181', '183', '184',
'185', '186', '187', '189', '18c', '190', '192', '196', '197', '198',
'199', '19a', '19b', '1ab', '1ad', '1ae', '1af', '1al', '1an', '1ap',
'1au', '1aw', '1ba', '1bd', '1bh', '1bm', '1bn', '1bo', '1bp', '1c5',
'1ca', '1cb', '1cd', '1cm', '1cn', '1cs', '1da', '1db', '1dg', '1dm',
'1do', '1em', '1fa', '1fn', '1fr', '1gl', '1gn', '1gp', '1hp', '1ig',
'1in', '1ip', '1iq', '1lg', '1lp', '1lu', '1mc', '1mm', '1mr', '1ms',
'1n5', '1n8', '1n9', '1nb', '1np', '1oh', '1p1', '1p3', '1pe', '1pg',
'1pi', '1pl', '1pm', '1ps', '1pu', '1rb', '1sc', '1sm', '1sq', '1st',
'1tb', '1un', '1zn', '1zx', '201', '203', '204', '207', '208', '209',
'210', '213', '214', '215', '216', '217', '21u', '223', '225', '226',
'227', '228', '22f', '22m', '22u', '230', '233', '234', '235', '237',
'238', '239', '23a', '23c', '23i', '23m', '23n', '23t', '240', '242',
'243', '244', '245', '246', '247', '24a', '24b', '24f', '24i', '24u',
'24x', '251', '252', '253', '254', '256', '258', '259', '25d', '260',
'261', '262', '263', '264', '265', '266', '267', '268', '269', '26a',
'26c', '26d', '26p', '26u', '271', '272', '273', '274', '276', '277',
'27a', '27u', '280', '281', '282', '283', '284', '287', '289', '291',
'293', '294', '295', '296', '298', '299', '29u', '2a6', '2ab', '2ac',
'2ad', '2af', '2ah', '2al', '2am', '2an', '2ao', '2aq', '2ar', '2as',
'2ba', '2bc', '2bf', '2bm', '2bn', '2bp', '2br', '2c2', '2c7', '2ca',
'2cc', '2ce', '2cg', '2ch', '2cl', '2cm', '2cp', '2cs', '2d9', '2da',
'2dg', '2di', '2dt', '2e1', '2e3', '2ep', '2ez', '2fa', '2fd', '2ff',
'2fl', '2fm', '2fr', '2fu', '2g0', '2gg', '2gj', '2gs', '2hc', '2hi',
'2ht', '2ib', '2ic', '2ig', '2ih', '2im', '2in', '2kt', '2lg', '2lp',
'2md', '2me', '2mg', '2mh', '2mi', '2mm', '2mn', '2mp', '2na', '2nc',
'2nd', '2ni', '2np', '2oa', '2ob', '2oh', '2op', '2os', '2pa', '2pe',
'2pg', '2pm', '2pn', '2po', '2pp', '2pu', '2rd', '2s2', '2s8', '2sc',
'2sk', '2sp', '2t1', '2t3', '2tb', '2un', '301', '302', '303', '304',
'306', '307', '308', '311', '313', '314', '316', '318', '319', '320',
'322', '324', '325', '328', '329', '32p', '330', '331', '332', '334',
'335', '338', '33z', '340', '341', '343', '344', '347', '348', '34a',
'34c', '34d', '34o', '34q', '34t', '34z', '352', '356', '358', '35a',
'35b', '35f', '369', '371', '373', '37a', '37t', '37u', '380', '382',
'383', '385', '388', '391', '393', '394', '396', '397', '3a3', '3ac',
'3ad', '3al', '3as', '3at', '3ay', '3b4', '3b6', '3b8', '3b9', '3ba',
'3bb', '3bd', '3bm', '3bp', '3br', '3bz', '3c4', '3cb', '3cc', '3ch',
'3cl', '3cn', '3cp', '3cs', '3cu', '3cz', '3d1', '3d3', '3db', '3dd',
'3dg', '3dh', '3ea', '3ep', '3fa', '3fm', '3fp', '3fr', '3ga', '3gc',
'3gp', '3gr', '3ha', '3hb', '3hc', '3hd', '3hg', '3hi', '3hl', '3hp',
'3ht', '3hx', '3ib', '3id', '3ig', '3ih', '3il', '3in', '3io', '3ip',
'3l1', '3lp', '3lr', '3mb', '3me', '3mf', '3mg', '3mo', '3mr', '3na',
'3np', '3nt', '3oc', '3oh', '3ol', '3pb', '3pg', '3pl', '3po', '3pp',
'3py', '3qc', '3sa', '3sc', '3sl', '3tc', '3tl', '3tn', '3tr', '3un',
'3xh', '3yp', '406', '411', '414', '417', '41a', '421', '426', '429',
'434', '43a', '43b', '43m', '440', '444', '446', '447', '44b', '452',
'458', '45p', '45u', '460', '464', '46c', '46m', '46u', '471', '474',

```
'478', '47d', '485', '493', '497', '49a', '49u', '4a3', '4aa', '4ab',
'4ad', '4af', '4ah', '4am', '4an', '4at', '4ax', '4ba', '4bm', '4bo',
'4bq', '4bs', '4bt', '4bu', '4bz', '4ca', '4cp', '4cs', '4de', '4di',
'4ea', '4eb', '4fa', '4fb', '4fc', '4fe', '4fp', '4gc', '4gp', '4ha',
'4hc', '4hf', '4hl', '4hp', '4hx', '4hy', '4ip', '4lg', '4ma', '4mb',
'4mc', '4mm', '4mr', '4mu', '4mv', '4na', '4nb', '4nc', '4nh', '4ni',
'4nl', '4np', '4ox', '4p5', '4pa', '4pb', '4pg', '4ph', '4pi', '4pp',
'4pr', '4qc', '4rb', '4sp', '4sr', '4st', '4su', '4tb', '4tn', '4tp',
'4tr', '4tz', '4un', '501', '505', '509', '50u', '512', '515', '51u',
'521', '528', '52a', '52h', '537', '53h', '53u', '541', '544', '545',
'547', '54d', '54h', '553', '555', '55e', '561', '564', '565', '566',
'567', '568', '572', '575', '578', '580', '582', '587', '588', '59a',
'5ac', '5an', '5ap', '5at', '5ax', '5b1', '5b2', '5b3', '5bm', '5br',
'5ca', '5cd', '5ch', '5cl', '5cm', '5cn', '5dp', '5ea', '5ee', '5f1',
'5fa', '5fe', '5fh', '5fp', '5h', '5hd', '5hg', '5ht', '5i5', '5id',
'5ig', '5in', '5ip', '5iq', '5iu', '5mb', '5mc', '5md', '5mp', '5mr',
'5ms', '5mu', '5nh', '5ni', '5ns', '5ph', '5pi', '5pp', '5pv', '5qc',
'5rm', '5rp', '5sd', '5tn', '5ud', '5x', '5yl', '600', '605', '606',
'608', '612', '616', '617', '61e', '624', '626', '629', '62p', '632',
'64p', '653', '655', '65b', '665', '666', '667', '669', '672', '675',
'678', '679', '682', '685', '687', '689', '693', '694', '697', '6ap',
'6c3', '6cm', '6cp', '6cs', '6de', '6ea', '6fa', '6gp', '6ha', '6hc',
'6hg', '6hi', '6ht', '6ig', '6in', '6ip', '6mp', '6mr', '6mz', '6na',
'6ni', '6np', '6ob', '6pc', '6pg', '6ph', '6pr', '6sa', '6ul', '700',
'701', '709', '70u', '710', '712', '716', '736', '738', '739', '73q',
'73v', '740', '74m', '750', '757', '75v', '761', '76v', '770', '771',
'772', '773', '778', '77a', '77b', '784', '785', '787', '789', '78a',
'78p', '790', '791', '794', '797', '7a8', '7ac', '7ap', '7ck', '7cp',
'7cs', '7gp', '7hi', '7hp', '7i2', '7ig', '7in', '7ip', '7mr', '7ni',
'7pa', '7pc', '7pe', '7pg', '7ph', '7py', '7ra', '7rp', '7x1', '7x2',
'7x4', '7x5', '7x6', '7x7', '7x8', '7xy', '809', '815', '817', '818',
'822', '824', '825', '82a', '839', '83h', '843', '852', '853', '857',
'858', '859', '85a', '866', '869', '86a', '870', '876', '877', '878',
'882', '885', '886', '892', '893', '897', '89i', '8an', '8ap', '8br',
'8c5', '8ca', '8cm', '8da', '8gt', '8hg', '8ig', '8in', '8ip', '8mo',
'8nh', '8og', '8pe', '8pg', '8pp', '8ps', '900', '901', '903', '905',
'910', '915', '922', '928', '941', '959', '95a', '961', '964', '968',
'977', '982', '983', '985', '989', '993', '994', '997', '998', '9ac',
'9ad', '9am', '9ap', '9ar', '9ca', '9cs', '9da', '9ho', '9hp', '9mg',
'9mr', '9nh', '9od', '9oh', '9pe', '9pp', 'A00', 'A05', 'A0a', 'A11',
'A12', 'A13', 'A15', 'A1a', 'A1e', 'A1r', 'A21', 'A22', 'A23', 'A24',
'A25', 'A2d', 'A2e', 'A2p', 'A2r', 'A2t', 'A32', 'A33', 'A34', 'A35',
'A37', 'A3b', 'A3m', 'A3p', 'A3s', 'A40', 'A41', 'A42', 'A44', 'A45',
'A46', 'A47', 'A51', 'A55', 'A56', 'A58', 'A5a', 'A5p', 'A6p', 'A74',
'A75', 'A76', 'A77', 'A79', 'A80', 'A83', 'A84', 'A88', 'A8m', 'A91',
'A94', 'A96', 'Aa1', 'Aa2', 'Aa6', 'Aa7', 'Aaa', 'Aab', 'Aac', 'Aae',
'Aaf', 'Aah', 'Aam', 'Aan', 'Aao', 'Aap', 'Aat', 'Aau', 'Aay', 'Ab0',
'Ab1', 'Ab3', 'Ab4', 'Ab6', 'Ab8', 'Ab9', 'Abc', 'Abe', 'Abf', 'Abg',
'Abj', 'Abm', 'Abn', 'Abo', 'Abp', 'Abr', 'Abs', 'Abu', 'Abw', 'Abx',
'Aby', 'Abz', 'Ac0', 'Ac1', 'Ac5', 'Ac6', 'Aca', 'Acb', 'Acc', 'Acg',
'Ach', 'Aci', 'Acj', 'Ack', 'Acl', 'Acm', 'Acn', 'Aco', 'Acp', 'Acq',
'Acr', 'Acs', 'Act', 'Acv', 'Acx', 'Acy', 'Ad0', 'Ad1', 'Ad3', 'Ad4',
'Ad5', 'Ad7', 'Ad8', 'Ada', 'Adb', 'Ade', 'Adf', 'Adh', 'Adi', 'Adl',
'Adm', 'Adn', 'Ado', 'Adp', 'Adq', 'Adr', 'Adt', 'Adv', 'Adx', 'Ady',
'Adz', 'Ae1', 'Ae2', 'Ae3', 'Ae4', 'Aea', 'Aed', 'Aee', 'Aef', 'Aeg',
'Aeh', 'Aej', 'Aem', 'Aen', 'Aes', 'Aet', 'Afb', 'Aff', 'Afh', 'Afi',
'Afj', 'Afn', 'Afp', 'Ag3', 'Ag7', 'Agb', 'Age', 'Agf', 'Agi', 'Agl',
'Agn', 'Agp', 'Ags', 'Agu', 'Ah0', 'Ahc', 'Ahd', 'Ahe', 'Ahf', 'Ahg',
'Ahh', 'Ahi', 'Ahl', 'Ahm', 'Ahn', 'Aho', 'Ahr', 'Ahs', 'Ahu', 'Ahx',
'Ahy', 'Ahz', 'Ai1', 'Ai7', 'Aia', 'Aic', 'Aif', 'Aig', 'Aih', 'Aij',
```

```
'Aik', 'Ain', 'Aiq', 'Ait', 'Aiu', 'Aiz', 'Aj3', 'Aja', 'Ajb', 'Ak1',
'Ak8', 'Akb', 'Akg', 'Akn', 'Akr', 'Akt', 'Al0', 'Al1', 'Al4', 'Al6',
'Al8', 'Ald', 'Ale', 'Ali', 'Alj', 'All', 'Alm', 'Alp', 'Alq', 'Alr',
'Als', 'Alu', 'Alw', 'Alz', 'Am0', 'Am2', 'Am3', 'Am4', 'Am5', 'Am6',
'Am8', 'Am9', 'Amc', 'Amd', 'Ame', 'Amf', 'Amg', 'Amh', 'Ami', 'Amk',
'Aml', 'Amn', 'Amo', 'Amp', 'Amr', 'Ams', 'Amu', 'Amv', 'Amx', 'An0',
'An2', 'An3', 'Ana', 'Anb', 'Anc', 'And', 'Anf', 'Anh', 'Ani', 'Anl',
'Anm', 'Ann', 'Ano', 'Anp', 'Anq', 'Ans', 'Ant', 'Anu', 'Anx', 'Ao2',
'Ao3', 'Ao5', 'Aoa', 'Aoe', 'Aog', 'Aol', 'Aom', 'Aon', 'Aop', 'Aor',
'Aos', 'Ap', 'Ap1', 'Ap3', 'Ap4', 'Ap5', 'Ap6', 'Ap9', 'Apa', 'Apc',
'Apd', 'Ape', 'Apf', 'Apg', 'Api', 'Apl', 'Apo', 'App', 'Apq', 'Apr',
'Aps', 'Apu', 'Apv', 'Apy', 'Apz', 'Aqo', 'Aqp', 'Aqs', 'Aqz', 'Ar1',
'Ar2', 'Ar3', 'Ar9', 'Ara', 'Arb', 'Are', 'Arf', 'Ari', 'Arj', 'Arl',
'Arm', 'Arn', 'Arp', 'Arq', 'Arw', 'As3', 'As5', 'Asa', 'Asc', 'Asd',
'Ase', 'Asg', 'Asi', 'Ask', 'Aso', 'Asu', 'Asv', 'Asy', 'At1', 'At2',
'At3', 'At4', 'At5', 'At6', 'Ata', 'Atd', 'Ate', 'Atf', 'Atg', 'Ath',
'Ati', 'Atj', 'Atl', 'Atm', 'Atn', 'Ato', 'Atq', 'Atr', 'Atu', 'Atx',
'Aty', 'Atz', 'Aua', 'Aup', 'Aur', 'Avd', 'Ave', 'Avf', 'Avg', 'Ax2',
'Ax4', 'Ax5', 'Ax6', 'Axb', 'Axd', 'Axf', 'Axl', 'Axp', 'Axq', 'Ayb',
'Ayd', 'Aye', 'Aym', 'Az1', 'Az2', 'Azc', 'Azf', 'Azg', 'Azk', 'Azl',
'Azn', 'Azo', 'Azp', 'Azr', 'Azz', 'B11', 'B14', 'B18', 'B19', 'B1l',
'B1p', 'B1v', 'B2', 'B21', 'B23', 'B28', 'B29', 'B2g', 'B2y', 'B33',
'B3a', 'B3d', 'B3e', 'B3h', 'B3i', 'B3k', 'B3s', 'B3x', 'B3y', 'B4c',
'B4g', 'B5r', 'B65', 'B67', 'B68', 'B6d', 'B75', 'B76', 'B7g', 'B7n',
'B8l', 'Ba3', 'Baa', 'Bac', 'Bad', 'Baf', 'Bai', 'Bak', 'Bal', 'Bas',
'Bau', 'Bav', 'Bax', 'Bb1', 'Bb2', 'Bb3', 'Bba', 'Bbc', 'Bbf', 'Bbh',
'Bbl', 'Bbm', 'Bbs', 'Bbt', 'Bbu', 'Bbx', 'Bc2', 'Bc6', 'Bcc', 'Bcd',
'Bcf', 'Bcg', 'Bch', 'Bcm', 'Bcn', 'Bco', 'Bcp', 'Bct', 'Bcy', 'Bd1',
'Bd4', 'Bdd', 'Bde', 'Bdf', 'Bdg', 'Bdh', 'Bdk', 'Bdp', 'Bdr', 'Bds',
'Bdt', 'Be2', 'Be3', 'Be4', 'Be5', 'Be6', 'Beb', 'Bec', 'Bed', 'Bee',
'Beg', 'Beh', 'Bei', 'Bej', 'Bek', 'Bel', 'Bem', 'Beo', 'Bes', 'Bet',
'Beu', 'Bey', 'Bez', 'Bfb', 'Bfi', 'Bfl', 'Bfn', 'Bfq', 'Bfs', 'Bg6',
'Bgc', 'Bgd', 'Bgg', 'Bgl', 'Bgn', 'Bgp', 'Bgs', 'Bgt', 'Bgu', 'Bh0',
'Bh7', 'Bha', 'Bhb', 'Bhc', 'Bhe', 'Bhf', 'Bhg', 'Bhh', 'Bhl', 'Bhm',
'Bho', 'Bhp', 'Bhq', 'Bhx', 'Bi1', 'Bi5', 'Bi7', 'Bi9', 'Bia', 'Bib',
'Bic', 'Bid', 'Bie', 'Big', 'Bih', 'Bim', 'Bio', 'Bip', 'Biq', 'Bir',
'Bis', 'Bit', 'Bix', 'Bl0', 'Bl4', 'Bl5', 'Bl6', 'Bl7', 'Blg', 'Bli',
'Bll', 'Bln', 'Blo', 'Blp', 'Bls', 'Blv', 'Bm1', 'Bm2', 'Bm3', 'Bm5',
'Bm6', 'Bma', 'Bmd', 'Bme', 'Bml', 'Bmm', 'Bmp', 'Bmq', 'Bn2', 'Bn3',
'Bn4', 'Bn5', 'Bna', 'Bnd', 'Bne', 'Bnf', 'Bng', 'Bnp', 'Bns', 'Bnt',
'Bnz', 'Bo1', 'Boa', 'Bog', 'Bom', 'Bop', 'Bos', 'Bp1', 'Bp3', 'Bp4',
'Bp5', 'Bp6', 'Bp7', 'Bpe', 'Bpf', 'Bpi', 'Bpl', 'Bpm', 'Bpn', 'Bpq',
'Bps', 'Bpy', 'Bqm', 'Bqn', 'Br4', 'Bra', 'Brb', 'Brc', 'Bre', 'Brf',
'Brj', 'Brk', 'Brl', 'Brn', 'Brp', 'Brr', 'Brs', 'Brt', 'Bru', 'Brw',
'Bry', 'Bsa', 'Bsb', 'Bsd', 'Bsi', 'Bsj', 'Bsm', 'Bso', 'Bst', 'Bsu',
'Bt2', 'Bt3', 'Bti', 'Btl', 'Btm', 'Btn', 'Btq', 'Bts', 'Btt', 'Btx',
'Bty', 'Bu1', 'Bu2', 'Bu3', 'Bui', 'Bul', 'Bum', 'Buq', 'Bvc', 'Bvd',
'Bvf', 'Bvl', 'Bvp', 'Bwd', 'Bwp', 'Bxp', 'Bxz', 'Bym', 'Byp', 'Bys',
'Bz1', 'Bz9', 'Bzc', 'Bzd', 'Bzf', 'Bzg', 'Bzi', 'Bzn', 'Bzp', 'Bzq',
'Bzr', 'Bzs', 'Bzt', 'Bzz', 'C00', 'C02', 'C0m', 'C0r', 'C10', 'C11',
'C14', 'C16', 'C17', 'C18', 'C19', 'C1f', 'C1m', 'C1p', 'C1r', 'C20',
'C26', 'C27', 'C2a', 'C2b', 'C2e', 'C2g', 'C2m', 'C2n', 'C2p', 'C2r',
'C2u', 'C33', 'C37', 'C38', 'C39', 'C3a', 'C3b', 'C3d', 'C3m', 'C3p',
'C3s', 'C3y', 'C41', 'C42', 'C45', 'C46', 'C47', 'C48', 'C4a', 'C4b',
'C4c', 'C4e', 'C4f', 'C4h', 'C4p', 'C52', 'C53', 'C5a', 'C5b', 'C5g',
'C6l', 'C6p', 'C71', 'C75', 'C78', 'C7m', 'C8c', 'C8e', 'C8m', 'C90',
'C92', 'C95', 'C96', 'C99', 'Ca1', 'Ca2', 'Caa', 'Cab', 'Cae', 'Cah',
'Cak', 'Cam', 'Can', 'Cao', 'Cap', 'Caq', 'Car', 'Cat', 'Cau', 'Cav',
'Caz', 'Cb3', 'Cbc', 'Cbd', 'Cbf', 'Cbg', 'Cbh', 'Cbi', 'Cbl', 'Cbo',
'Cbp', 'Cbq', 'Cbs', 'Cbt', 'Cbu', 'Cbv', 'Cbw', 'Cc1', 'Cc5', 'Cca',
```

```
'Ccc', 'Ccd', 'Cce', 'Cck', 'Ccm', 'Ccn', 'Cco', 'Ccp', 'Ccr', 'Cct',
'Ccv', 'Cde', 'Cdf', 'Cdg', 'Cdi', 'Cdm', 'Cdo', 'Cdp', 'Cdr', 'Cds',
'Cdt', 'Cdu', 'Cdv', 'Cdx', 'Cdy', 'Cdz', 'Ce1', 'Ce3', 'Ce5', 'Ce6',
'Ceb', 'Ceg', 'Cei', 'Cej', 'Cel', 'Cen', 'Ceo', 'Cer', 'Cet', 'Cf3',
'Cf4', 'Cfa', 'Cfb', 'Cff', 'Cfl', 'Cfp', 'Cfs', 'Cft', 'Cgb', 'Cgn',
'Cgr', 'Cgs', 'Cgt', 'Ch1', 'Ch4', 'Ch5', 'Cha', 'Chb', 'Chc', 'Chd',
'Chf', 'Chg', 'Chh', 'Chi', 'Chn', 'Chr', 'Chs', 'Cht', 'Chx', 'Chy',
'Ci2', 'Cia', 'Cib', 'Cic', 'Cie', 'Cii', 'Cin', 'Cip', 'Cir', 'Cis',
'Cit', 'Cjb', 'Ck1', 'Ck2', 'Ck4', 'Ck5', 'Ck6', 'Ck7', 'Ck8', 'Ck9',
'Ckb', 'Cke', 'Ckr', 'Cl4', 'Cl5', 'Cl8', 'Cl9', 'Clc', 'Clk', 'Cll',
'Clm', 'Clq', 'Clr', 'Clt', 'Clw', 'Clx', 'Cly', 'Cm0', 'Cm3', 'Cm4',
'Cm5', 'Cm6', 'Cm7', 'Cm9', 'Cmc', 'Cmd', 'Cmf', 'Cmg', 'Cmi', 'Cmk',
'Cmp', 'Cmq', 'Cms', 'Cmu', 'Cmw', 'Cmx', 'Cmz', 'Cn3', 'Cn5', 'Cn6',
'Cne', 'Cnh', 'Cni', 'Cnl', 'Cnp', 'Cnq', 'Cnr', 'Cns', 'Cny', 'Co1',
'Co2', 'Co7', 'Co8', 'Co9', 'Coc', 'Cod', 'Cof', 'Coi', 'Col', 'Com',
'Cop', 'Cor', 'Cos', 'Cot', 'Cou', 'Cov', 'Cox', 'Coz', 'Cp', 'Cp1',
'Cp2', 'Cp3', 'Cp4', 'Cp5', 'Cp6', 'Cp7', 'Cp8', 'Cp9', 'Cpa', 'Cpb',
'Cpd', 'Cph', 'Cpi', 'Cpj', 'Cpk', 'Cpq', 'Cpu', 'Cpw', 'Cpy', 'Cqp',
'Cqu', 'Cqw', 'Cr0', 'Cr1', 'Cr3', 'Cr4', 'Cr6', 'Cra', 'Crb', 'Crd',
'Crf', 'Crh', 'Cri', 'Crm', 'Crp', 'Crq', 'Crr', 'Crs', 'Crz', 'Cs2',
'Cs7', 'Cs8', 'Cs9', 'Csf', 'Csn', 'Cst', 'Ct0', 'Ct1', 'Ct2', 'Ct3',
'Ct4', 'Ct5', 'Cta', 'Ctd', 'Cte', 'Ctf', 'Ctl', 'Ctn', 'Cto', 'Ctp',
'Cts', 'Ctt', 'Ctx', 'Ctz', 'Cwr', 'Cx6', 'Cxa', 'Cxf', 'Cxl', 'Cxn',
'Cxo', 'Cxp', 'Cxr', 'Cxs', 'Cxt', 'Cxx', 'Cxy', 'Cxz', 'Cy0', 'Cy9',
'Cyh', 'Cyi', 'Cyk', 'Cyt', 'Cyv', 'Cyx', 'Cyy', 'Cyz', 'Czh', 'Czm',
'D01', 'D12', 'D15', 'D1d', 'D1h', 'D1l', 'D1n', 'D1r', 'D1t', 'D23',
'D26', 'D27', 'D28', 'D2a', 'D2g', 'D2p', 'D2v', 'D31', 'D3t', 'D4d',
'D4g', 'D4m', 'D4t', 'D55', 'D56', 'D6g', 'D6p', 'D76', 'D7p', 'D8w',
'D92', 'D93', 'D9g', 'D9z', 'D_Iyt', 'Da1', 'Da3', 'Da7', 'Da8',
'Dac', 'Dad', 'Dai', 'Dak', 'Dan', 'Dao', 'Dat', 'Dav', 'Db0', 'Db9',
'Dba', 'Dbd', 'Dbe', 'Dbf', 'Dbh', 'Dbi', 'Dbl', 'Dbm', 'Dbn', 'Dbo',
'Dbp', 'Dbr', 'Dc8', 'Dca', 'Dcb', 'Dcc', 'Dcd', 'Dce', 'Dcf', 'Dci',
'Dcl', 'Dcm', 'Dcn', 'Dco', 'Dcp', 'Dcr', 'Dcs', 'Dct', 'Dcx', 'Dcz',
'Dd1', 'Dd2', 'Dd3', 'Dda', 'Ddc', 'Ddf', 'Ddi', 'Ddl', 'Ddo', 'Ddp',
'Ddr', 'Ddt', 'Ddu', 'Ddy', 'De1', 'Dea', 'Deb', 'Dec', 'Dee', 'Deg',
'Del', 'Dem', 'Den', 'Deo', 'Dep', 'Der', 'Des', 'Dex', 'Dez', 'Df1',
'Df2', 'Dfa', 'Dfb', 'Dfc', 'Dfd', 'Dfe', 'Dfi', 'Dfj', 'Dfl', 'Dfm',
'Dfn', 'Dfo', 'Dfp', 'Dfr', 'Dft', 'Dfu', 'Dfv', 'Dfw', 'Dfx', 'Dfy',
'Dfz', 'Dg0', 'Dg1', 'Dg3', 'Dg6', 'Dgb', 'Dgc', 'Dgt', 'Dgy', 'Dh1',
'Dh2', 'Dh9', 'Dhb', 'Dhd', 'Dhf', 'Dhh', 'Dhk', 'Dhl', 'Dhp', 'Dhr',
'Dhs', 'Dht', 'Dhy', 'Dhz', 'Di6', 'Dia', 'Dib', 'Dif', 'Dig', 'Dii',
'Din', 'Dio', 'Dip', 'Diq', 'Diu', 'Dix', 'Diy', 'Diz', 'Djk', 'Dk1',
'Dk2', 'Dka', 'Dl6', 'Dl8', 'Dlf', 'Dlg', 'Dli', 'Dm1', 'Dm2', 'Dm3',
'Dm4', 'Dm5', 'Dm6', 'Dm7', 'Dm8', 'Dm9', 'Dma', 'Dmb', 'Dmd', 'Dme',
'Dmf', 'Dmg', 'Dmj', 'Dmm', 'Dmn', 'Dmq', 'Dmr', 'Dms', 'Dmv', 'Dmw',
'Dmz', 'Dn2', 'Dnc', 'Dnf', 'Dnh', 'Dnl', 'Dnn', 'Dno', 'Dnq', 'Do2',
'Do3', 'Do4', 'Doa', 'Dob', 'Doc', 'Dod', 'Doe', 'Dof', 'Dog', 'Dom',
'Doq', 'Dor', 'Dp1', 'Dp2', 'Dp3', 'Dp6', 'Dp9', 'Dpa', 'Dpc', 'Dpe',
'Dpg', 'Dph', 'Dpj', 'Dpk', 'Dpo', 'Dps', 'Dpt', 'Dpu', 'Dpz', 'Dqa',
'Dqb', 'Dqh', 'Dqn', 'Dqo', 'Dqq', 'Dqr', 'Dqu', 'Dr0', 'Dr1', 'Dr3',
'Dr5', 'Dr9', 'Dra', 'Drc', 'Drd', 'Drf', 'Drg', 'Drh', 'Dri', 'Drj',
'Drk', 'Drm', 'Drn', 'Dro', 'Drq', 'Drs', 'Drt', 'Drx', 'Dry', 'Dsa',
'Dsd', 'Dsm', 'Dso', 'Dsr', 'Dss', 'Dst', 'Dsu', 'Dsv', 'Dsy', 'Dt',
'Dt1', 'Dt6', 'Dt8', 'Dta', 'Dtb', 'Dtd', 'Dte', 'Dtf', 'Dtl', 'Dtm',
'Dtn', 'Dto', 'Dtp', 'Dtq', 'Dtt', 'Dtu', 'Dtv', 'Dtx', 'Duc', 'Dud',
'Dun', 'Dup', 'Dur', 'Dut', 'Dux', 'Dvr', 'Dvv', 'Dx1', 'Dx2', 'Dx3',
'Dx5', 'Dx6', 'Dx7', 'Dx9', 'Dxc', 'Dxe', 'Dxg', 'Dxp', 'Dxx', 'Dy6',
'Dyg', 'Dym', 'Dyp', 'Dz8', 'Dzd', 'Dzf', 'Dzn', 'Dzp', 'Dzz', 'E04',
'E10', 'E12', 'E1h', 'E1p', 'E20', 'E2m', 'E2p', 'E3g', 'E3o', 'E4d',
'E4h', 'E4p', 'E7b', 'E89', 'E96', 'E97', 'Ea1', 'Ea2', 'Eah', 'Eal',
```

```
'Eap', 'Ebp', 'Ebs', 'Ebw', 'Eca', 'Ecg', 'Eco', 'Ecq', 'Ecs', 'Ect',
'Ed5', 'Ed7', 'Edo', 'Edr', 'Edt', 'Eed', 'Eee', 'Ees', 'Efs', 'Eg2',
'Eg3', 'Egd', 'Egt', 'Eh9', 'Eha', 'Ehd', 'Ehm', 'Ehn', 'Ei1', 'Eic',
'Eip', 'Eke', 'Ela', 'Eld', 'Elp', 'Ema', 'Emb', 'Emd', 'Emm', 'Emo',
'Emr', 'Emu', 'End', 'Ene', 'Enh', 'Enm', 'Eno', 'Ens', 'Enx', 'Eoa',
'Eoh', 'Eop', 'Eot', 'Ep', 'Ep0', 'Ep1', 'Ep2', 'Epc', 'Epe', 'Eph',
'Epi', 'Epj', 'Epl', 'Epm', 'Epn', 'Epq', 'Eps', 'Ept', 'Epu', 'Epx',
'Eqi', 'Eqp', 'Equ', 'Erd', 'Erg', 'Eri', 'Ert', 'Esa', 'Esh', 'Esl',
'Esm', 'Eso', 'Esr', 'Est', 'Et0', 'Et1', 'Eta', 'Etc', 'Etd', 'Ete',
'Etf', 'Etm', 'Etn', 'Etv', 'Etx', 'Ety', 'Eug', 'Exo', 'Eyk', 'Ezl',
'Ezt', 'Ezv', 'F09', 'F11', 'F12', 'F15', 'F16', 'F17', 'F1h', 'F1i',
'F1j', 'F1k', 'F1l', 'F1m', 'F1n', 'F1p', 'F20', 'F21', 'F23', 'F29',
'F2a', 'F2b', 'F2i', 'F2o', 'F32', 'F3b', 'F3g', 'F50', 'F55', 'F59',
'F5b', 'F68', 'F6b', 'F6f', 'F6p', 'F6r', 'F77', 'F79', 'F83', 'F89',
'F8a', 'F9f', 'Fa1', 'Fa3', 'Fa4', 'Fa5', 'Fa6', 'Fac', 'Fad', 'Faf',
'Fai', 'Fal', 'Fam', 'Fan', 'Fao', 'Fap', 'Fas', 'Fb', 'Fba', 'Fbc',
'Fbd', 'Fbe', 'Fbi', 'Fbl', 'Fbp', 'Fbq', 'Fbr', 'Fbs', 'Fbt', 'Fbu',
'Fc1', 'Fc2', 'Fca', 'Fcb', 'Fcn', 'Fcp', 'Fcr', 'Fct', 'Fcx', 'Fd2',
'Fd3', 'Fd4', 'Fda', 'Fdi', 'Fdm', 'Fdn', 'Fdp', 'Fdq', 'Fds', 'Fdt',
'Fe1', 'Fe3', 'Feb', 'Fef', 'Feo', 'Fep', 'Fer', 'Ff3', 'Ffa', 'Ffb',
'Ffc', 'Fff', 'Ffo', 'Ffp', 'Fga', 'Fgr', 'Fhb', 'Fhc', 'Fhi', 'Fhm',
'Fhp', 'Fhu', 'Fib', 'Fic', 'Fid', 'Fii', 'Fil', 'Fin', 'Fip', 'Fir',
'Fis', 'Fka', 'Fkd', 'Fki', 'Fl2', 'Fl8', 'Fl9', 'Flc', 'Flf', 'Flm',
'Fln', 'Flp', 'Flv', 'Flx', 'Fm1', 'Fma', 'Fmm', 'Fmn', 'Fmp', 'Fmr',
'Fms', 'Fmt', 'Fmx', 'Fng', 'Fnh', 'Fnp', 'Fnr', 'Fnz', 'Fo1', 'Foc',
'Foh', 'Fol', 'Fom', 'Fon', 'Fop', 'For', 'Fos', 'Fot', 'Fp2', 'Fpa',
'Fpc', 'Fpd', 'Fpe', 'Fph', 'Fpi', 'Fpm', 'Fpn', 'Fpo', 'Fpp', 'Fpr',
'Fps', 'Fpt', 'Fpy', 'Fqp', 'Fqx', 'Fr0', 'Fr1', 'Fr2', 'Fr4', 'Fr5',
'Fr7', 'Fr9', 'Fra', 'Frc', 'Frd', 'Frf', 'Fri', 'Frm', 'Fro', 'Frq',
'Frr', 'Frt', 'Fru', 'Frz', 'Fsa', 'Fsb', 'Fsc', 'Fsg', 'Fsh', 'Fsi',
'Fsm', 'Fsp', 'Ftc', 'Fth', 'Ftl', 'Fts', 'Ftt', 'Fu4', 'Fuc', 'Fud',
'Fuf', 'Ful', 'Fum', 'Fun', 'Fup', 'Fur', 'Fvf', 'Fwd', 'Fx3', 'Fxg',
'Fxn', 'Fxp', 'Fxy', 'Fya', 'Fyn', 'Fyx', 'G0g', 'G12', 'G19', 'G1l',
'G1p', 'G23', 'G24', 'G26', 'G27', 'G2p', 'G2r', 'G36', 'G37', 'G38',
'G39', 'G3a', 'G3d', 'G3g', 'G3h', 'G3p', 'G44', 'G4d', 'G4g', 'G4s',
'G50', 'G55', 'G6d', 'G6p', 'G6q', 'G6s', 'G88', 'G93', 'G95', 'G96',
'Ga3', 'Ga4', 'Ga9', 'Gaa', 'Gab', 'Gad', 'Gaf', 'Gag', 'Gah', 'Gai',
'Gaj', 'Gal', 'Gam', 'Gaq', 'Gar', 'Gas', 'Gat', 'Gau', 'Gax', 'Gb1',
'Gb2', 'Gb3', 'Gb4', 'Gb5', 'Gb6', 'Gb7', 'Gbd', 'Gbi', 'Gbl', 'Gbn',
'Gbs', 'Gbx', 'Gc1', 'Gca', 'Gcg', 'Gch', 'Gcn', 'Gco', 'Gcp', 'Gcq',
'Gcs', 'Gct', 'Gcu', 'Gcv', 'Gcw', 'Gd7', 'Gd9', 'Gdl', 'Gdr', 'Gdt',
'Gdu', 'Ge1', 'Ge3', 'Gea', 'Geg', 'Gem', 'Gen', 'Geo', 'Gep', 'Geq',
'Gfa', 'Gfl', 'Gfp', 'Gg1', 'Gg2', 'Gg3', 'Gg5', 'Gg6', 'Gg7', 'Gg9',
'Gga', 'Ggc', 'Ggh', 'Ggo', 'Ggz', 'Gig', 'Gin', 'Gio', 'Giq', 'Gir',
'Gis', 'Gk1', 'Gke', 'Gkr', 'Gl1', 'Gl2', 'Gl4', 'Gl5', 'Gl6', 'Gl7',
'Gl9', 'Gla', 'Glc', 'Gld', 'Glf', 'Glg', 'Gll', 'Glm', 'Glo', 'Glp',
'Glr', 'Gls', 'Glt', 'Glv', 'Glz', 'Gm6', 'Gmc', 'Gmh', 'Gmp', 'Gmy',
'Gn1', 'Gn7', 'Gn8', 'Gnf', 'Gng', 'Gnh', 'Gni', 'Gnq', 'Gnr', 'Gns',
'Gnt', 'Goa', 'Gol', 'Gox', 'Gp1', 'Gp2', 'Gp3', 'Gp4', 'Gp8', 'Gpb',
'Gpd', 'Gpe', 'Gpf', 'Gph', 'Gpi', 'Gpj', 'Gpo', 'Gpp', 'Gpr', 'Gps',
'Gpx', 'Gr1', 'Gr3', 'Gr4', 'Grd', 'Gre', 'Grf', 'Grg', 'Grl', 'Gro',
'Grr', 'Gs1', 'Gs5', 'Gs6', 'Gs7', 'Gsb', 'Gsc', 'Gse', 'Gsf', 'Gsh',
'Gsi', 'Gsj', 'Gsk', 'Gsl', 'Gsm', 'Gsn', 'Gsq', 'Gss', 'Gsv', 'Gtl',
'Gtm', 'Gtp', 'Gtr', 'Gts', 'Gtt', 'Gtx', 'Gty', 'Gu3', 'Gu4', 'Gu7',
'Gua', 'Gud', 'Gum', 'Gup', 'Gva', 'Gvb', 'Gve', 'Gvh', 'Gvi', 'Gvj',
'Gvk', 'Gvl', 'Gvm', 'Gvn', 'Gvo', 'Gvp', 'Gvq', 'Gvr', 'Gvt', 'Gw3',
'Gw6', 'Gw7', 'Gw9', 'Gwe', 'Gwi', 'Gwj', 'Gxl', 'Gyc', 'Gyp', 'Gyt',
'Gyu', 'Gzz', 'H11', 'H12', 'H16', 'H18', 'H1d', 'H1l', 'H1m', 'H20',
'H23', 'H24', 'H2b', 'H2p', 'H2s', 'H2u', 'H35', 'H3s', 'H4b', 'H4p',
'H52', 'H53', 'H5p', 'H64', 'H71', 'H7j', 'Ha2', 'Ha8', 'Hab', 'Hae',
```

```
'Hag', 'Hai', 'Hal', 'Han', 'Hav', 'Hax', 'Hba', 'Hbb', 'Hbd', 'Hbh',
'Hbi', 'Hbm', 'Hbo', 'Hbq', 'Hbr', 'Hbs', 'Hbu', 'Hby', 'Hbz', 'Hc2',
'Hc3', 'Hc4', 'Hc5', 'Hc6', 'Hca', 'Hcc', 'Hci', 'Hcp', 'Hct', 'Hcy',
'Hda', 'Hdc', 'Hdf', 'Hdi', 'Hdn', 'Hds', 'Hdu', 'Hdy', 'Hdz', 'He1',
'He2', 'He3', 'He4', 'He7', 'Hed', 'Hee', 'Hef', 'Hel', 'Hep', 'Het',
'Hex', 'Hez', 'Hf1', 'Hfa', 'Hfb', 'Hfl', 'Hfs', 'Hft', 'Hga', 'Hh1',
'Hh2', 'Hhg', 'Hhh', 'Hho', 'Hhr', 'Hi1', 'Hi5', 'Hib', 'Hii', 'Hio',
'Hiu', 'Hj3', 'Hle', 'Hlt', 'Hm2', 'Hm4', 'Hma', 'Hmc', 'Hmg', 'Hmh',
'Hmi', 'Hmk', 'Hmm', 'Hmn', 'Hmo', 'Hmr', 'Hms', 'Hmu', 'Hna', 'Hnb',
'Hne', 'Hnt', 'Hoa', 'Hoc', 'Hoe', 'Hoh', 'Hom', 'Hop', 'Hos', 'Hoz',
'Hp2', 'Hp6', 'Hpd', 'Hpf', 'Hpi', 'Hpk', 'Hpl', 'Hpm', 'Hpn', 'Hpo',
'Hpp', 'Hpr', 'Hps', 'Hpt', 'Hpv', 'Hpy', 'Hqc', 'Hqo', 'Hqq', 'Hqu',
'Hs1', 'Hs2', 'Hs3', 'Hs4', 'Hs5', 'Hs6', 'Hs7', 'Hse', 'Hsf', 'Hsg',
'Hsh', 'Hsi', 'Hsp', 'Hsq', 'Hsr', 'Hss', 'Hsu', 'Hsw', 'Hsx', 'Hsy',
'Hsz', 'Ht', 'Hta', 'Htf', 'Htg', 'Htm', 'Hto', 'Htq', 'Hu1', 'Hu2',
'Hu4', 'Hu5', 'Hux', 'Hv1', 'Hv7', 'Hv8', 'Hwd', 'Hwg', 'Hxd', 'Hy1',
'Hy3', 'Hya', 'Hyb', 'Hyc', 'Hyi', 'Hym', 'Hyn', 'Hyq', 'Hyz', 'Hz3',
'I01', 'I03', 'I04', 'I05', 'I06', 'I08', 'I0g', 'I10', 'I12', 'I17',
'I1h', 'I1n', 'I1p', 'I21', 'I24', 'I25', 'I26', 'I2c', 'I2p', 'I38',
'I3a', 'I3c', 'I3n', 'I3p', 'I3s', 'I40', 'I4a', 'I4b', 'I4p', 'I50',
'I59', 'I5p', 'I5s', 'I7a', 'I7b', 'I7c', 'I84', 'Ia', 'Iab', 'Iac',
'Iag', 'Ib', 'Ib2', 'Iba', 'Ibc', 'Ibf', 'Ibg', 'Ibm', 'Ibn', 'Ibo',
'Ibp', 'Ibr', 'Ibu', 'Ibz', 'Ic', 'Ic1', 'Icc', 'Icf', 'Icl', 'Icn',
'Ico', 'Icp', 'Icr', 'Ict', 'Icx', 'Id2', 'Id5', 'Ida', 'Idb', 'Idd',
'Idg', 'Idh', 'Idi', 'Idm', 'Idn', 'Idr', 'Ids', 'Idu', 'Idx', 'Idz',
'Iem', 'Ifa', 'Ifb', 'Ifc', 'Ifg', 'Ifl', 'Ig', 'Igp', 'Ih3', 'Ih5',
'Ihb', 'Ihd', 'Ihe', 'Ihg', 'Ihj', 'Ihn', 'Ihp', 'Ihu', 'Ihx', 'Ihz',
'Iib', 'Iid', 'Iie', 'Iin', 'Iip', 'Ik8', 'Ikt', 'Ila', 'Ilb', 'Ilc',
'Ili', 'Ilo', 'Ilp', 'Im1', 'Im2', 'Im3', 'Im4', 'Im8', 'Im9', 'Ima',
'Imc', 'Img', 'Imh', 'Imi', 'Imn', 'Imo', 'Imr', 'Ims', 'In0', 'In1',
'In2', 'In3', 'In7', 'In9', 'Ina', 'Inb', 'Inc', 'Ind', 'Ine', 'Inf',
'Ing', 'Inh', 'Ini', 'Inj', 'Ink', 'Inl', 'Ino', 'Inp', 'Ins', 'Int',
'Inv', 'Inw', 'Inx', 'Inz', 'Ioa', 'Iob', 'Ioc', 'Ioe', 'Iof', 'Iog',
'Iok', 'Iol', 'Ion', 'Iop', 'Iot', 'Ip1', 'Ip2', 'Ip3', 'Ip4', 'Ip5',
'Ip6', 'Ip7', 'Ipa', 'Ipb', 'Ipc', 'Ipd', 'Ipe', 'Ipf', 'Iph', 'Ipl',
'Ipm', 'Ipo', 'Ipp', 'Ipr', 'Ipt', 'Ipy', 'Ipz', 'Iqb', 'Iqp', 'Iqs',
'Iqu', 'Iqz', 'Ire', 'Is2', 'Isa', 'Isc', 'Isd', 'Isn', 'Iso', 'Isp',
'Isq', 'Isu', 'Isx', 'Isz', 'Ita', 'Itb', 'Itp', 'Itr', 'Itt', 'Itu',
'Iu5', 'Iur', 'Iva', 'Ivs', 'Iwd', 'Ix1', 'Ixh', 'Ixm', 'Ixs', 'Ixx',
'Iyg', 'Iz1', 'Iz2', 'Iz3', 'Iz4', 'Iz5', 'Iza', 'Izb', 'Izc', 'Izd',
'Ize', 'Izf', 'Izz', 'J01', 'J07', 'J54', 'J60', 'J72', 'J77', 'J78',
'J80', 'J88', 'Jan', 'Jas', 'Jb1', 'Jc1', 'Jcb', 'Jcl', 'Je2', 'Jen',
'Ji1', 'Ji2', 'Ji3', 'Ji7', 'Jin', 'Jj3', 'Jk1', 'Jk2', 'Jn3', 'Jn5',
'Jnh', 'Jnj', 'Jnk', 'Jos', 'Jp1', 'Jpa', 'Jpc', 'Jpj', 'Jpl', 'Jpm',
'Jpn', 'Jt5', 'Jt6', 'Jtp', 'Ju2', 'Jug', 'Jy', 'K02', 'K05', 'K10',
'K17', 'K21', 'K22', 'K25', 'K2c', 'K2z', 'K30', 'K32', 'K37', 'K44',
'K51', 'K57', 'K7i', 'K7j', 'Kaf', 'Kah', 'Kai', 'Kan', 'Kap', 'Kaw',
'Kbg', 'Kcp', 'Kcq', 'Kda', 'Kdb', 'Kdd', 'Kde', 'Kdf', 'Kdg', 'Kdo',
'Kdp', 'Kdr', 'Kel', 'Kgc', 'Kgy', 'Kha', 'Kho', 'Khp', 'Kif', 'Kim',
'Kin', 'Kiv', 'Kmb', 'Kmt', 'Kn1', 'Kn2', 'Knc', 'Kni', 'Ko1', 'Ko2',
'Koj', 'Kom', 'Kos', 'Kpa', 'Kpc', 'Kph', 'Kpl', 'Kpv', 'Kr2', 'Kra',
'Krb', 'Kri', 'Ks1', 'Ks5', 'Ksa', 'Ksf', 'Ksg', 'Ksm', 'Kta', 'Kth',
'Ku8', 'Ky1', 'L01', 'L02', 'L03', 'L04', 'L05', 'L06', 'L08', 'L09',
'L0f', 'L0h', 'L12', 'L13', 'L17', 'L1a', 'L1g', 'L1o', 'L1r', 'L20',
'L24', 'L27', 'L2c', 'L35', 'L41', 'L4c', 'L4g', 'L75', 'L86', 'L92',
'L98', 'La1', 'Lab', 'Lac', 'Lad', 'Lae', 'Laf', 'Lag', 'Lak', 'Laq',
'Lar', 'Lat', 'Laz', 'Lbt', 'Lbv', 'Lca', 'Lcc', 'Lcg', 'Lcx', 'Ldc',
'Ldo', 'Ldt', 'Ldy', 'Lea', 'Leg', 'Leh', 'Len', 'Lep', 'Lfa', 'Lfc',
'Lfn', 'Lfr', 'Lg3', 'Lg4', 'Lg5', 'Lg6', 'Lg7', 'Lga', 'Lgc', 'Lgd',
'Lgg', 'Lgp', 'Lgs', 'Lgt', 'Lgu', 'Lgz', 'Lha', 'Lhg', 'Lhy', 'Li2',
```

```
'Li3', 'Li4', 'Li5', 'Li6', 'Li7', 'Li8', 'Li9', 'Lia', 'Lib', 'Lic',
'Lid', 'Lie', 'Lif', 'Lig', 'Lih', 'Lii', 'Lij', 'Lik', 'Lil', 'Lim',
'Lio', 'Lip', 'Liq', 'Lir', 'Lis', 'Liu', 'Liv', 'Liw', 'Lix', 'Liy',
'Lj1', 'Lj2', 'Lj3', 'Lj4', 'Lj5', 'Ljg', 'Ljh', 'Lk1', 'Lk2', 'Lk4',
'Lka', 'Lkc', 'Lkm', 'Lks', 'Llb', 'Llc', 'Llg', 'Llh', 'Lll', 'Lm2',
'Lmg', 'Lml', 'Lmr', 'Lms', 'Lmt', 'Lmu', 'Lmz', 'Lnq', 'Lnr', 'Lnt',
'Lob', 'Loc', 'Lop', 'Lor', 'Lov', 'Lox', 'Lp1', 'Lp3', 'Lp4', 'Lp5',
'Lpa', 'Lpl', 'Lpm', 'Lpp', 'Lrg', 'Lrh', 'Ls1', 'Ls2', 'Ls3', 'Ls4',
'Ls5', 'Lsa', 'Lsp', 'Ltl', 'Ltn', 'Ltt', 'Lum', 'Luz', 'Lva', 'Lx1',
'Lx2', 'Lxb', 'Lxc', 'Lxp', 'Lxz', 'Ly1', 'Ly2', 'Ly3', 'Ly5', 'Lyd',
'Lyl', 'Lym', 'Lyp', 'Lyt', 'Lyw', 'Lz1', 'Lz2', 'Lz3', 'Lz4', 'Lz5',
'Lz6', 'Lz7', 'Lz8', 'Lza', 'Lzb', 'Lze', 'Lzj', 'Lzk', 'Lzl', 'Lzm',
'M01', 'M02', 'M03', 'M04', 'M05', 'M07', 'M08', 'M11', 'M13', 'M14',
'M18', 'M1b', 'M1l', 'M1p', 'M1s', 'M22', 'M25', 'M28', 'M29', 'M2c',
'M2f', 'M2m', 'M38', 'M5p', 'M6d', 'M6p', 'M6r', 'M77', 'M7p', 'M8c',
'M8e', 'M8m', 'Ma1', 'Ma2', 'Ma3', 'Ma4', 'Ma5', 'Ma6', 'Ma9', 'Mab',
'Mac', 'Mad', 'Mag', 'Mah', 'Mai', 'Maj', 'Mak', 'Mal', 'Man', 'Mao',
'Maq', 'Mar', 'Mas', 'Maw', 'Max', 'Maz', 'Mb0', 'Mb1', 'Mb9', 'Mbb',
'Mbc', 'Mbd', 'Mbf', 'Mbg', 'Mbh', 'Mbn', 'Mbo', 'Mbp', 'Mbr', 'Mbs',
'Mbz', 'Mc2', 'Mca', 'Mcb', 'Mcd', 'Mcm', 'Mcn', 'Mco', 'Mcp', 'Mcs',
'Mct', 'Mcy', 'Md2', 'Mda', 'Mdd', 'Mdl', 'Mdm', 'Mdn', 'Mdr', 'Mdw',
'Mdx', 'Mdz', 'Me1', 'Me2', 'Me4', 'Me5', 'Mea', 'Mec', 'Mee', 'Mef',
'Mer', 'Mev', 'Mf2', 'Mfg', 'Mfm', 'Mfp', 'Mfq', 'Mfr', 'Mfu', 'Mgb',
'Mgc', 'Mgd', 'Mge', 'Mgi', 'Mgl', 'Mgs', 'Mgt', 'Mgx', 'Mha', 'Mhb',
'Mhc', 'Mhf', 'Mhn', 'Mhr', 'Mhz', 'Mi1', 'Mi4', 'Mic', 'Mid', 'Mik',
'Mil', 'Min', 'Miu', 'Mix', 'Mk1', 'Mkc', 'Mke', 'Ml1', 'Ml2', 'Mla',
'Mld', 'Mlg', 'Mli', 'Mlk', 'Mlm', 'Mlr', 'Mlt', 'Mm0', 'Mma', 'Mmb',
'Mmi', 'Mmm', 'Mmq', 'Mmr', 'Mmz', 'Mn1', 'Mn2', 'Mn7', 'Mn8', 'Mna',
'Mnb', 'Mnc', 'Mng', 'Mni', 'Mnm', 'Mnn', 'Mno', 'Mnp', 'Mns', 'Mnt',
'Mnx', 'Mny', 'Mo9', 'Mob', 'Moe', 'Moh', 'Moi', 'Mop', 'Mor', 'Mou',
'Mox', 'Moy', 'Mp5', 'Mpb', 'Mpd', 'Mpe', 'Mph', 'Mpj', 'Mpm', 'Mpn',
'Mpo', 'Mpp', 'Mpr', 'Mpx', 'Mpz', 'Mq1', 'Mq7', 'Mqi', 'Mqq', 'Mqu',
'Mr0', 'Mr1', 'Mr2', 'Mr3', 'Mr4', 'Mr5', 'Mr6', 'Mr9', 'Mra', 'Mrc',
'Mrd', 'Mre', 'Mrk', 'Mrm', 'Mrp', 'Mru', 'Mrx', 'Mry', 'Ms1', 'Ms2',
'Ms3', 'Msa', 'Msc', 'Msg', 'Msi', 'Msm', 'Msn', 'Msq', 'Mst', 'Msu',
'Mt3', 'Mt4', 'Mta', 'Mtb', 'Mte', 'Mtf', 'Mtg', 'Mth', 'Mtj', 'Mtl',
'Mtn', 'Mtp', 'Mts', 'Mtt', 'Mtw', 'Mtx', 'Mtz', 'Mu0', 'Mu1', 'Mu2',
'Mua', 'Muc', 'Mud', 'Mug', 'Muh', 'Mup', 'Mur', 'Mus', 'Muw', 'Mx3',
'Mx4', 'Mx5', 'Mxa', 'Mxe', 'Mxg', 'Mxp', 'Mxt', 'Mxy', 'Mxz', 'My2',
'My3', 'My4', 'My5', 'My9', 'Myd', 'Myf', 'Myg', 'Myp', 'Myr', 'Mys',
'Myt', 'Myu', 'Myx', 'Myy', 'Mz1', 'Mz2', 'Mz3', 'Mz4', 'Mz5', 'Mz6',
'Mz7', 'Mz8', 'Mz9', 'Mzp', 'N', 'N12', 'N20', 'N22', 'N2m', 'N2o',
'N30', 'N31', 'N33', 'N3b', 'N3c', 'N3e', 'N3h', 'N3p', 'N41', 'N4b',
'N4d', 'N4e', 'N5a', 'N5c', 'N5f', 'N5o', 'N5p', 'N69', 'N76', 'N7p',
'N8e', 'N9h', 'Na4', 'Na7', 'Naa', 'Nab', 'Nad', 'Nae', 'Naf', 'Nag',
'Nai', 'Naj', 'Nal', 'Nar', 'Nas', 'Nat', 'Nav', 'Nax', 'Nay', 'Naz',
'Nb1', 'Nbb', 'Nbc', 'Nbd', 'Nbe', 'Nbg', 'Nbl', 'Nbn', 'Nbp', 'Nbs',
'Nbt', 'Nbv', 'Nbx', 'Nby', 'Nbz', 'Nc3', 'Nc4', 'Nc5', 'Nc6', 'Nc7',
'Nca', 'Ncc', 'Ncd', 'Nce', 'Nci', 'Ncj', 'Nck', 'Ncl', 'Ncm', 'Ncq',
'Ncr', 'Ncs', 'Nct', 'Ncx', 'Ncz', 'Nda', 'Ndd', 'Ndg', 'Ndh', 'Ndl',
'Ndn', 'Ndo', 'Ndp', 'Ndr', 'Ne1', 'Ne2', 'Ne8', 'Nea', 'Nec', 'Neg',
'Neh', 'Nei', 'Nen', 'Neo', 'Neq', 'Nes', 'Net', 'Neu', 'Nex', 'Nez',
'Nfg', 'Nfl', 'Nfn', 'Nft', 'Nfz', 'Ng1', 'Ng6', 'Nga', 'Ngc', 'Ngh',
'Ngk', 'Ngm', 'Ngr', 'Ngs', 'Ngt', 'Ngu', 'Ngy', 'Ngz', 'Nh1', 'Nh3',
'Nh4', 'Nh7', 'Nh8', 'Nhb', 'Nhd', 'Nhe', 'Nhp', 'Nhr', 'Nht', 'Nhy',
'Nia', 'Nic', 'Nid', 'Nie', 'Nig', 'Nii', 'Nil', 'Nim', 'Nio', 'Nis',
'Nit', 'Nk', 'Nkk', 'Nla', 'Nlc', 'Nlp', 'Nlt', 'Nm1', 'Nmb', 'Nmd',
'Nme', 'Nmg', 'Nmh', 'Nml', 'Nmp', 'Nmu', 'Nmy', 'Nn1', 'Nnb', 'Nnc',
'Nnd', 'Nng', 'Nni', 'Nnn', 'Nno', 'Noa', 'Noe', 'Nog', 'Noj', 'Nok',
'Nol', 'Non', 'Noq', 'Nor', 'Nos', 'Noz', 'Np1', 'Np2', 'Np4', 'Np5',
```

```
'Npa', 'Npd', 'Npf', 'Npg', 'Npi', 'Npj', 'Npm', 'Npn', 'Npo', 'Nps',
'Npt', 'Npv', 'Npy', 'Npz', 'Nq', 'Nqg', 'Nqi', 'Nr2', 'Nrb', 'Nri',
'Nro', 'Nrq', 'Nrt', 'Nsa', 'Nsb', 'Nsc', 'Nsi', 'Nsk', 'Nsm', 'Nsp',
'Nss', 'Nst', 'Nt', 'Nta', 'Ntb', 'Ntc', 'Ntd', 'Ntf', 'Nth', 'Ntj',
'Ntm', 'Ntz', 'Nu1', 'Nu5', 'Nup', 'Nvc', 'Nvg', 'Nw1', 'Nwa', 'Nxa',
'Nxn', 'Nya', 'Nyh', 'Nyl', 'Nym', 'Nza', 'Nzq', 'O1c', 'O33', 'O8m',
'Oaa', 'Oaf', 'Oai', 'Oal', 'Oan', 'Oap', 'Oba', 'Obe', 'Obn', 'Obp',
'Oc9', 'Ocb', 'Och', 'Ocp', 'Ocq', 'Oct', 'Ocv', 'Ocx', 'Odd', 'Odi',
'Ods', 'Odt', 'Oef', 'Oeg', 'Oes', 'Oev', 'Off', 'Ofl', 'Oga', 'Ogg',
'Oha', 'Ohh', 'Ohm', 'Ohn', 'Oho', 'Ohp', 'Oht', 'Ois', 'Oka', 'Ola',
'Olc', 'Oln', 'Olo', 'Olu', 'Omc', 'Omd', 'Omn', 'Omp', 'Omu', 'One',
'Onl', 'Ooa', 'Opb', 'Ope', 'Opg', 'Oph', 'Opm', 'Opn', 'Opp', 'Opq',
'Opr', 'Oqb', 'Oro', 'Orp', 'Orx', 'Osb', 'Osm', 'Osp', 'Oss', 'Ost',
'Osu', 'Ota', 'Otd', 'Otg', 'Otr', 'Ots', 'Out', 'Ox1', 'Ox2', 'Ox3',
'Ox4', 'Ox6', 'Ox7', 'Oxd', 'Oxe', 'Oxg', 'Oxk', 'Oxl', 'Oxm', 'Oxp',
'Oxq', 'Oxs', 'Oxy', 'Oxz', 'Oya', 'P01', 'P10', 'P12', 'P14', 'P15',
'P16', 'P19', 'P1a', 'P1r', 'P1s', 'P1t', 'P1z', 'P20', 'P21', 'P22',
'P23', 'P24', 'P25', 'P27', 'P28', 'P2a', 'P2g', 'P2k', 'P2n', 'P2o',
'P2p', 'P2s', 'P2u', 'P2y', 'P32', 'P33', 'P39', 'P3d', 'P3m', 'P3s',
'P3t', 'P3y', 'P41', 'P44', 'P45', 'P4a', 'P4c', 'P4g', 'P4t', 'P5a',
'P5b', 'P5c', 'P5p', 'P63', 'P6g', 'P83', 'P90', 'P91', 'Pa1', 'Pa2',
'Pa3', 'Pa5', 'Pa6', 'Pa7', 'Pab', 'Pac', 'Paf', 'Pah', 'Paj', 'Pak',
'Pal', 'Pam', 'Pan', 'Pao', 'Pap', 'Par', 'Pau', 'Pav', 'Paw', 'Pay',
'Pb3', 'Pb4', 'Pb5', 'Pb9', 'Pbe', 'Pbn', 'Pbo', 'Pbp', 'Pbr', 'Pbs',
'Pbu', 'Pc', 'Pcb', 'Pce', 'Pch', 'Pci', 'Pcm', 'Pcn', 'Pco', 'Pcp',
'Pcq', 'Pcr', 'Pcs', 'Pct', 'Pcv', 'Pcx', 'Pcy', 'Pd1', 'Pd2', 'Pd3',
'Pd5', 'Pd7', 'Pd8', 'Pdc', 'Pde', 'Pdg', 'Pdh', 'Pdj', 'Pdn', 'Pdo',
'Pdp', 'Pds', 'Pdt', 'Pdz', 'Pe0', 'Pe2', 'Pe3', 'Pe4', 'Pe5', 'Pe6',
'Pe7', 'Pe8', 'Pe9', 'Pea', 'Pef', 'Peg', 'Pel', 'Pem', 'Pep', 'Peq',
'Per', 'Pey', 'Pf1', 'Pf7', 'Pfa', 'Pfb', 'Pfd', 'Pfe', 'Pfh', 'Pfi',
'Pfj', 'Pfl', 'Pfp', 'Pfq', 'Pg0', 'Pg2', 'Pg3', 'Pg4', 'Pg5', 'Pg6',
'Pga', 'Pge', 'Pgf', 'Pgg', 'Pgh', 'Pgj', 'Pgl', 'Pgo', 'Pgr', 'Pgt',
'Pgu', 'Pgv', 'Pgx', 'Ph0', 'Ph2', 'Ph4', 'Ph5', 'Phb', 'Phc', 'Phg',
'Phh', 'Phk', 'Phm', 'Phn', 'Phq', 'Phr', 'Phs', 'Pht', 'Phy', 'Phz',
'Pi0', 'Pi1', 'Pi2', 'Pi3', 'Pi4', 'Pi5', 'Pi6', 'Pi7', 'Pi8', 'Pi9',
'Pib', 'Pic', 'Pih', 'Pil', 'Pip', 'Pir', 'Pit', 'Piv', 'Pkf', 'Pl0',
'Pl2', 'Pl3', 'Pl4', 'Pl5', 'Pl6', 'Pl7', 'Plb', 'Plh', 'Plm', 'Plo',
'Plp', 'Plq', 'Plr', 'Pls', 'Plt', 'Plu', 'Plv', 'Plz', 'Pm1', 'Pm4',
'Pm5', 'Pm6', 'Pm9', 'Pma', 'Pmb', 'Pmc', 'Pme', 'Pmh', 'Pmi', 'Pml',
'Pmm', 'Pmo', 'Pmp', 'Pms', 'Pmv', 'Pn2', 'Pn5', 'Pna', 'Pnb', 'Pnc',
'Pnd', 'Pnf', 'Png', 'Pnh', 'Pnm', 'Pnn', 'Pno', 'Pnp', 'Pns', 'Pnu',
'Pnv', 'Pnx', 'Pnz', 'Po0', 'Po1', 'Po4', 'Poa', 'Pob', 'Pof', 'Poi',
'Pol', 'Pon', 'Poo', 'Pop', 'Pos', 'Pox', 'Pp1', 'Ppc', 'Ppf', 'Ppi',
'Ppk', 'Ppl', 'Ppo', 'Ppp', 'Ppq', 'Ppr', 'Ppt', 'Ppv', 'Ppy', 'Ppz',
'Pq0', 'Pq9', 'Pqa', 'Pqb', 'Pqn', 'Pr0', 'Pr1', 'Pr2', 'Pra', 'Prc',
'Prd', 'Prf', 'Pri', 'Prl', 'Prn', 'Prp', 'Prq', 'Prx', 'Pry', 'Ps0',
'Psa', 'Psd', 'Pse', 'Psi', 'Psk', 'Psl', 'Psm', 'Pso', 'Psq', 'Pst',
'Psu', 'Psx', 'Psy', 'Psz', 'Pt1', 'Pt3', 'Pta', 'Ptd', 'Ptf', 'Pti',
'Ptl', 'Pto', 'Ptu', 'Pu0', 'Pu1', 'Pu2', 'Pu3', 'Pu4', 'Pu6', 'Pu7',
'Pu8', 'Pu9', 'Pua', 'Pug', 'Pul', 'Put', 'Pux', 'Puy', 'Puz', 'Pva',
'Pvs', 'Pxa', 'Pxf', 'Pxg', 'Pxi', 'Pxl', 'Pxm', 'Pxp', 'Pxy', 'Pxz',
'Py0', 'Py1', 'Py2', 'Py7', 'Py9', 'Pyc', 'Pyd', 'Pye', 'Pyf', 'Pyg',
'Pyh', 'Pyl', 'Pym', 'Pyn', 'Pyo', 'Pyq', 'Pyr', 'Pys', 'Pyt', 'Pyu',
'Pyy', 'Pyz', 'Pz2', 'Pza', 'Pzd', 'Pzf', 'Pzl', 'Pzm', 'Pzo', 'Pzq',
'Q22', 'Q4a', 'Q50', 'Qga', 'Qgg', 'Qms', 'Qn1', 'Qn2', 'Qnc', 'Qnd',
'Qno', 'Qpp', 'Qps', 'Qq2', 'Qsi', 'Qso', 'Qui', 'Qun', 'Qus', 'Qv4',
'Qyt', 'R', 'R01', 'R03', 'R04', 'R10', 'R11', 'R13', 'R15', 'R16',
'R17', 'R18', 'R19', 'R1p', 'R20', 'R22', 'R23', 'R2c', 'R36', 'R37',
'R46', 'R51', 'R52', 'R55', 'R56', 'R64', 'R68', 'R69', 'R6c', 'R71',
'R79', 'R86', 'R8d', 'R8e', 'R94', 'R96', 'R99', 'Ra2', 'Ra4', 'Ra8',
```

```
'Rab', 'Rac', 'Rad', 'Rae', 'Rai', 'Raj', 'Ral', 'Ram', 'Rao', 'Rap',
'Ras', 'Raz', 'Rb1', 'Rbc', 'Rbf', 'Rbp', 'Rbs', 'Rbz', 'Rc1', 'Rca',
'Rcl', 'Rco', 'Rcp', 'Rda', 'Rdd', 'Rde', 'Rdf', 'Rdl', 'Rdr', 'Re9',
'Rec', 'Red', 'Res', 'Rew', 'Rex', 'Rey', 'Rez', 'Rf5', 'Rfl', 'Rfz',
'Rgi', 'Rgp', 'Rgt', 'Rha', 'Rhc', 'Rhp', 'Rhs', 'Ria', 'Rib', 'Rid',
'Rie', 'Rim', 'Rin', 'Rio', 'Rip', 'Rit', 'Rj1', 'Rka', 'Rl2', 'Rm1',
'Rm2', 'Rm4', 'Rma', 'Rmb', 'Rmn', 'Rna', 'Rnp', 'Rns', 'Rnt', 'Ro1',
'Ro2', 'Ro4', 'Rob', 'Roc', 'Rok', 'Rol', 'Rop', 'Rp1', 'Rp4', 'Rp5',
'Rpd', 'Rpf', 'Rph', 'Rpl', 'Rpn', 'Rpr', 'Rq3', 'Rr6', 'Rrc', 'Rrp',
'Rrs', 'Rrt', 'Rs1', 'Rs2', 'Rs7', 'Rsa', 'Rsh', 'Rss', 'Rsx', 'Rtr',
'Rub', 'Rut', 'Rvp', 'Rw1', 'Rwf', 'Rwj', 'Rxa', 'Rxb', 'Rxd', 'Rxp',
'S01', 'S03', 'S0h', 'S10', 'S11', 'S17', 'S1a', 'S1t', 'S24', 'S27',
'S2a', 'S2d', 'S3p', 'S48', 'S4m', 'S57', 'S58', 'S5h', 'S60', 'S6p',
'S70', 'S79', 'S80', 'S98', 'S9l', 'Sa1', 'Sa2', 'Sa8', 'Sab', 'Saf',
'Sag', 'Sah', 'Sai', 'Sak', 'Sal', 'San', 'Sap', 'Sas', 'Sat', 'Sav',
'Sb1', 'Sb4', 'Sb7', 'Sb8', 'Sb9', 'Sba', 'Sbb', 'Sbc', 'Sbg', 'Sbi',
'Sbn', 'Sbr', 'Sbs', 'Sbt', 'Sc', 'Sc0', 'Sc1', 'Sc2', 'Sc4', 'Sc6',
'Sc7', 'Sc8', 'Sc9', 'Scc', 'Scd', 'Scg', 'Sci', 'Scj', 'Sck', 'Scl',
'Scm', 'Scq', 'Scr', 'Scv', 'Scw', 'Scx', 'Sd2', 'Sda', 'Sdc', 'Sdk',
'Sdn', 'Sds', 'Sdx', 'Sdz', 'Seh', 'Sei', 'Sel', 'Sem', 'Ses', 'Sf1',
'Sf2', 'Sfg', 'Sfr', 'Sg1', 'Sg2', 'Sg3', 'Sga', 'Sgb', 'Sgc', 'Sgi',
'Sgl', 'Sgm', 'Sgn', 'Sgp', 'Sgs', 'Sh1', 'Sha', 'Shb', 'Shf', 'Shg',
'Shh', 'Shi', 'Shm', 'Sho', 'Shr', 'Sht', 'Shu', 'Shv', 'Shy', 'Si1',
'Sia', 'Sig', 'Sih', 'Sim', 'Sin', 'Sj1', 'Sk1', 'Sk3', 'Ska', 'Skd',
'Skf', 'Skm', 'Skp', 'Sl1', 'Sl2', 'Sl3', 'Sla', 'Slb', 'Sle', 'Slr',
'Slt', 'Slu', 'Sm1', 'Sm5', 'Smb', 'Smg', 'Smk', 'Sml', 'Smm', 'Smn',
'Smz', 'Sn0', 'Sn1', 'Sn2', 'Snl', 'Snn', 'Snp', 'Snr', 'Snx', 'So2',
'So3', 'So4', 'Soa', 'Sod', 'Sog', 'Son', 'Sop', 'Sor', 'Sot', 'Sox',
'Sp1', 'Sp5', 'Sp6', 'Sp7', 'Sp8', 'Sp9', 'Spb', 'Spd', 'Spe', 'Sph',
'Spi', 'Spj', 'Spm', 'Spp', 'Spq', 'Sps', 'Spv', 'Spy', 'Spz', 'Sqa',
'Squ', 'Sr1', 'Sr2', 'Sra', 'Srg', 'Sri', 'Srn', 'Sro', 'Srp', 'Srt',
'Ss1', 'Ss2', 'Ss3', 'Ss4', 'Ss5', 'Ssa', 'Ssb', 'Ssc', 'Ssd', 'Ssg',
'Ssh', 'Ssm', 'St1', 'St3', 'St4', 'St5', 'St8', 'Sta', 'Stb', 'Stc',
'Stf', 'Stg', 'Sti', 'Stl', 'Stn', 'Str', 'Stu', 'Stz', 'Suc', 'Sud',
'Suf', 'Sug', 'Sui', 'Sun', 'Suo', 'Sup', 'Sur', 'Svc', 'Svx', 'Svy',
'Svz', 'Swa', 'Swf', 'Sx', 'Sx1', 'Sx2', 'Sx3', 'Sx4', 'Sx5', 'Sx6',
'Sx7', 'Sxe', 'Sxx', 'Sy1', 'Syb', 'Sym', 'Syr', 'T10', 'T12', 'T1d',
'T1p', 'T23', 'T24', 'T25', 'T27', 'T2d', 'T2m', 'T3', 'T32', 'T33',
'T38', 'T39', 'T3a', 'T3f', 'T3o', 'T3p', 'T3q', 'T3s', 'T41', 'T44',
'T45', 'T48', 'T49', 'T4a', 'T4b', 'T4k', 'T4p', 'T50', 'T5a', 'T5p',
'T6a', 'T74', 'T76', 'T80', 'T8n', 'T95', 'Ta2', 'Ta5', 'Ta6', 'Tab',
'Taf', 'Tag', 'Tal', 'Tap', 'Taq', 'Tar', 'Tau', 'Tav', 'Tb0', 'Tbe',
'Tbf', 'Tbi', 'Tbn', 'Tbp', 'Tbs', 'Tbz', 'Tc4', 'Tc7', 'Tca', 'Tcb',
'Tcc', 'Tcd', 'Tce', 'Tck', 'Tcl', 'Tcm', 'Tcn', 'Tco', 'Tct', 'Tcz',
'Tda', 'Tdc', 'Tde', 'Tdg', 'Tdi', 'Tdm', 'Tdo', 'Tdr', 'Tds', 'Tdx',
'Tdz', 'Teb', 'Tei', 'Ten', 'Tep', 'Tes', 'Tet', 'Tf1', 'Tf2', 'Tf3',
'Tf4', 'Tf5', 'Tfa', 'Tfb', 'Tfc', 'Tfg', 'Tfi', 'Tfm', 'Tfp', 'Tfq',
'Tfs', 'Tgg', 'Tgn', 'Tgt', 'Tgu', 'Th0', 'Th2', 'Th4', 'Th5', 'Th8',
'Tha', 'Thd', 'The', 'Thf', 'Thh', 'Thi', 'Thj', 'Thk', 'Thl', 'Thm',
'Tho', 'Thp', 'Ths', 'Tht', 'Thv', 'Thx', 'Thz', 'Ti1', 'Ti2', 'Ti3',
'Tim', 'Tin', 'Tio', 'Tit', 'Tiz', 'Tk4', 'Tl3', 'Tl5', 'Tl6', 'Tla',
'Tlc', 'Tld', 'Tlm', 'Tln', 'Tlx', 'Tm2', 'Tma', 'Tme', 'Tmf', 'Tmg',
'Tmj', 'Tmm', 'Tmp', 'Tmr', 'Tms', 'Tmu', 'Tn1', 'Tn2', 'Tn3', 'Tn5',
'Tnd', 'Tnf', 'Tnk', 'Tnl', 'Tns', 'Tnt', 'Toa', 'Tob', 'Toc', 'Toh',
'Tol', 'Tom', 'Tos', 'Tou', 'Tp1', 'Tp2', 'Tp3', 'Tp4', 'Tp5', 'Tp6',
'Tp9', 'Tpb', 'Tpe', 'Tpi', 'Tpr', 'Tpv', 'Tpw', 'Tpx', 'Tpy', 'Tq3',
'Tq4', 'Tq5', 'Tq6', 'Tqp', 'Tr1', 'Tra', 'Trc', 'Trd', 'Tre', 'Trh',
'Tri', 'Trj', 'Trl', 'Trm', 'Trs', 'Tru', 'Trw', 'Trz', 'Ts0', 'Ts1',
'Ts5', 'Tsa', 'Tsb', 'Tsc', 'Tsf', 'Tsh', 'Tsi', 'Tsl', 'Tsm', 'Tsp',
'Tsr', 'Tss', 'Tst', 'Tsu', 'Tsz', 'Tt', 'Tt1', 'Tt2', 'Ttb', 'Tte',
```

```
'Ttf', 'Ttl', 'Ttn', 'Ttp', 'Ttt', 'Ttz', 'Tud', 'Tui', 'Tuo', 'Tux',
'Twt', 'Txf', 'Txs', 'Tyb', 'Tyd', 'Tyl', 'Tym', 'Typ', 'Tyu', 'Tyv',
'Tyx', 'Tzb', 'Tzc', 'Tzd', 'Tze', 'Tzl', 'Tzo', 'Tzp', 'Tzz', 'U04',
'U05', 'U0e', 'U11', 'U12', 'U13', 'U14', 'U15', 'U19', 'U1n', 'U20',
'U2f', 'U2g', 'U2p', 'U2s', 'U32', 'U33', 'U36', 'U37', 'U3p', 'U3s',
'U4s', 'U51', 'U55', 'U5p', 'U73', 'Ua1', 'Ua2', 'Ua3', 'Ua4', 'Ua5',
'Uaa', 'Uag', 'Uap', 'Uar', 'Ub1', 'Ubc', 'Ube', 'Ubf', 'Uc3', 'Uc4',
'Uc5', 'Ucd', 'Ucl', 'Ucm', 'Ucn', 'Ucp', 'Ud1', 'Ud2', 'Ud4', 'Ud5',
'Uda', 'Udh', 'Udp', 'Udt', 'Udx', 'Ufg', 'Ufm', 'Ufp', 'Ufr', 'Uga',
'Uhd', 'Ui2', 'Ui3', 'Uib', 'Uin', 'Uip', 'Uiq', 'Ukp', 'Um3', 'Uma',
'Umf', 'Umg', 'Un3', 'Un4', 'Un5', 'Un8', 'Una', 'Unb', 'Unc', 'Und',
'Unf', 'Ung', 'Unh', 'Unn', 'Up5', 'Up6', 'Upa', 'Upf', 'Upg', 'Upp',
'Upr', 'Upu', 'Ur2', 'Ura', 'Urb', 'Urc', 'Urd', 'Ure', 'Urf', 'Uri',
'Urn', 'Uro', 'Urp', 'Urs', 'Us1', 'Usq', 'Utp', 'Uvw', 'Uz9', 'V10',
'V12', 'V15', 'V20', 'V25', 'V2h', 'V55', 'Va', 'Vaa', 'Vac', 'Vad',
'Vaf', 'Vag', 'Vam', 'Vas', 'Vaw', 'Vb1', 'Vbz', 'Vca', 'Vd2', 'Vd3',
'Vd4', 'Vd5', 'Vda', 'Vdb', 'Vdm', 'Vdn', 'Vdx', 'Vg2', 'Vg3', 'Vg6',
'Vg7', 'Vg8', 'Vga', 'Vi', 'Vig', 'Vii', 'Vir', 'Vit', 'Viv', 'Vk3',
'Vme', 'Vnl', 'Vpr', 'Vr1', 'Vrs', 'Vrx', 'Vs1', 'Vs2', 'Vso', 'Vtq',
'Vx1', 'Vx3', 'Vxr', 'Vxx', 'W01', 'W02', 'W03', 'W11', 'W33', 'W35',
'W37', 'W42', 'W43', 'W54', 'W56', 'W59', 'W71', 'W72', 'W84', 'W8r',
'W91', 'Wac', 'Wai', 'Way', 'Wbt', 'Wbu', 'Wh6', 'Wia', 'Wr1', 'Wra',
'Wrb', 'Wrr', 'Wrs', 'Wsa', 'Wsk', 'Wst', 'Ww7', 'Wz1', 'Wz2', 'Wz3',
'Wz5', 'X04', 'X1p', 'X22', 'X23', 'X2f', 'X4a', 'X9a', 'Xad', 'Xae',
'Xal', 'Xan', 'Xao', 'Xap', 'Xat', 'Xbb', 'Xbp', 'Xcs', 'Xct', 'Xdl',
'Xdn', 'Xdp', 'Xe4', 'Xed', 'Xga', 'Xgl', 'Xif', 'Xih', 'Xil', 'Xim',
'Xin', 'Xk2', 'Xl3', 'Xld', 'Xlf', 'Xlm', 'Xls', 'Xma', 'Xmb', 'Xmc',
'Xmd', 'Xme', 'Xmf', 'Xmg', 'Xmh', 'Xmi', 'Xmj', 'Xmk', 'Xmm', 'Xn1',
'Xn2', 'Xn3', 'Xp1', 'Xpe', 'Xrg', 'Xtg', 'Xth', 'Xts', 'Xty', 'Xul',
'Xx3', 'Xx4', 'Xx5', 'Xx6', 'Xx7', 'Xxg', 'Xxz', 'Xy2', 'Xya', 'Xyd',
'Xyh', 'Xyl', 'Xyp', 'Xyq', 'Xys', 'Xyz', 'Y', 'Y11', 'Y12', 'Y13',
'Y14', 'Y15', 'Y27', 'Yam', 'Yan', 'Ybh', 'Yby', 'Yc2', 'Ydp', 'Ye1',
'Ye6', 'Yf1', 'Yll', 'Yly', 'Yma', 'Yml', 'Ymp', 'Ypa', 'Yr3', 'Yrg',
'Ysa', 'Ysh', 'Yvn', 'Yx1', 'Yyy', 'Yz9', 'Z34', 'Zad', 'Zaf', 'Zah',
'Zam', 'Zar', 'Zat', 'Zct', 'Zdp', 'Zdr', 'Zea', 'Zec', 'Zed', 'Zen',
'Zes', 'Zez', 'Zhh', 'Zhp', 'Zhz', 'Zip', 'Zk5', 'Zld', 'Zma', 'Zmg',
'Zmq', 'Zmr', 'Zpg', 'Zpq', 'Zpr', 'Zrg', 'Zst', 'Ztw', 'Zu3', 'Zya',
'Zyx', 'Zyz', 'Zz1', 'Zza', 'Zzz']

[5] Mols clean with PDB ONLY, besides [4]:
263     ['006', '03r', '0a5', '118', '153', '182', '194', '195',
'1ah', '1c2', '1fl', '205', '211', '219', '23d', '2kl', '2mc', '2nh',
'2pr', '2sa', '2st', '2xy', '346', '34p', '3ca', '3cy', '3ft', '3lg',
'3mm', '413', '44c', '4co', '4hg', '4mp', '4qb', '698', '699', '6ca',
'730', '803', '821', '864', '896', '8fg', '8mr', '991', '9ip', 'A19',
'A1t', 'A38', 'A3d', 'A43', 'A70', 'A85', 'A9a', 'Aa5', 'Abd', 'Acf',
'Ad6', 'Aeb', 'Ah1', 'Ai', 'Akp', 'Anj', 'Atc', 'Azd', 'Aze', 'Bah',
'Bbr', 'Bcr', 'Bik', 'Biz', 'Blt', 'Bmv', 'Btb', 'Bth', 'C03', 'C1b',
'C7p', 'Ca3', 'Ca5', 'Cbe', 'Cbn', 'Cc0', 'Cdb', 'Ceh', 'Ces', 'Cg',
'Cnd', 'Cok', 'Con', 'Ctc', 'Cup', 'Cw1', 'Cy6', 'Day', 'Ddd', 'Dh3',
'Dhq', 'Djr', 'Dmp', 'Doi', 'Dpx', 'Drr', 'Dxt', 'E64', 'Ea5', 'Eaa',
'Ead', 'Eb1', 'Ed4', 'Ein', 'Eli', 'Emc', 'Ent', 'Epa', 'F19', 'F2n',
'F42', 'Fab', 'Fen', 'Fk5', 'Fl1', 'Fns', 'Fpf', 'Frp', 'Fxm', 'Gan',
'Ger', 'Gpm', 'Gtb', 'H8h', 'Hbc', 'Heh', 'Hen', 'Hey', 'Htc', 'Hxa',
'Hxl', 'Hyf', 'Iad', 'Iav', 'Ilf', 'Ilh', 'Imz', 'Inr', 'Ios', 'Jno',
'Kar', 'Kir', 'L0c', 'L0e', 'L2g', 'Lax', 'Lg8', 'Ln1', 'Lqq', 'Lut',
'Lyc', 'M6t', 'Mae', 'Mau', 'Mci', 'Mez', 'Mgm', 'Mi2', 'Mkk', 'Mkr',
'Mm1', 'Mm6', 'Moa', 'Mp1', 'Mq9', 'Msb', 'Mui', 'Mut', 'Mvd', 'N1t',
'N3t', 'Na9', 'Ncg', 'Ncn', 'Nd4', 'Ndc', 'Neb', 'Nf', 'Ngv', 'Nix',
'Nmx', 'Nop', 'Nov', 'Nrg', 'Oad', 'Oi1', 'Ona', 'Osc', 'Otp', 'Ova',
```

```
'P0p', 'P13', 'P3f', 'Pad', 'Pbm', 'Pew', 'Ph1', 'Ph7', 'Pi', 'Ple',
'Pmd', 'Pmn', 'Pqq', 'Psn', 'Px1', 'Py3', 'Q82', 'Qn3', 'R78', 'Rea',
'Ret', 'Rom', 'S1s', 'Saz', 'Se4', 'Sii', 'Sk2', 'Smd', 'Sms', 'Sn3',
'Snd', 'Sng', 'Sp2', 'Spo', 'Spw', 'St6', 'T4s', 'Ta1', 'Tb9', 'Tbo',
'Tdp', 'Tg1', 'Thw', 'Thy', 'Til', 'Tm5', 'Tm6', 'Tne', 'Ton', 'Tsn',
'Tth', 'Udm', 'Ui1', 'Un7', 'Un9', 'Uq1', 'Uq2', 'Uq5', 'Uq7', 'Vd1',
'Vdz', 'Xcl', 'Xtl', 'Xug', 'Zid']

[6] Mols clean with IDEAL ONLY, besides [4]:
1238     ['001', '009', '00a', '012', '01a', '031', '061', '084',
'098', '099', '0as', '0jz', '0ze', '102', '10u', '11f', '127', '128',
'12d', '12h', '12u', '130', '139', '14w', '152', '166', '173', '193',
'19u', '1ac', '1ar', '1bu', '1by', '1cx', '1jz', '1ma', '1nt', '1pn',
'1pp', '1tp', '20a', '20e', '20s', '220', '221', '241', '250', '255',
'270', '279', '285', '288', '292', '297', '2at', '2au', '2be', '2bt',
'2bu', '2df', '2dm', '2ea', '2ed', '2fg', '2fp', '2gl', '2gt', '2ha',
'2hr', '2hs', '2ip', '2mb', '2mu', '2my', '2nt', '2ot', '2pd', '2rl',
'2s3', '2tn', '300', '315', '336', '339', '33p', '367', '38m', '39z',
'3af', '3am', '3an', '3b3', '3bc', '3bi', '3bn', '3bt', '3cm', '3da',
'3fq', '3mp', '3nh', '3od', '3pa', '3ph', '3pi', '3tp', '3ty', '418',
'42b', '433', '442', '44d', '459', '468', '46d', '4ac', '4bg', '4cm',
'4f3', '4fr', '4ig', '4md', '4nd', '4oc', '4pc', '4pd', '4pe', '4pn',
'4pt', '4r8', '4ta', '4tc', '529', '550', '585', '5aa', '5ad', '5as',
'5bu', '5cb', '5cp', '5fc', '5fd', '5fr', '5fu', '5ha', '5he', '5ic',
'5ob', '5op', '5pa', '5pc', '615', '641', '642', '64t', '688', '6ia',
'6ma', '6mc', '6mt', '6oc', '6og', '6pl', '715', '741', '744', '745',
'760', '79z', '7ad', '7ca', '7da', '7nh', '806', '81a', '826', '846',
'872', '887', '888', '8ad', '8gp', '8mg', '8pc', '936', '965', '979',
'9in', '9ta', 'A14', 'A16', 'A17', 'A26', 'A2g', 'A2l', 'A2m', 'A3a',
'A4c', 'A5m', 'A66', 'A8b', 'Aag', 'Aai', 'Aal', 'Ab2', 'Ab7', 'Abb',
'Abl', 'Acw', 'Acz', 'Ad2', 'Adc', 'Add', 'Ads', 'Adu', 'Aep', 'Af',
'Af1', 'Af4', 'Ag2', 'Ag6', 'Agg', 'Agt', 'Aht', 'Aip', 'Ajh', 'Ajm',
'Aka', 'Akc', 'Akl', 'Akv', 'Aky', 'Al3', 'Alg', 'Alh', 'Alt', 'Am1',
'Amb', 'Amy', 'An1', 'An9', 'Ao1', 'Ap2', 'Apn', 'Apx', 'Aq4', 'Arc',
'Arh', 'Arr', 'Arx', 'Ary', 'As0', 'As1', 'As4', 'Asf', 'Atp', 'Au4',
'Avn', 'Ax3', 'Axx', 'Ay1', 'Azq', 'Azt', 'Azx', 'B08', 'B2s', 'B3n',
'B3p', 'B4p', 'B66', 'B9a', 'Ba1', 'Ba2', 'Bag', 'Ban', 'Bao', 'Bap',
'Bbb', 'Bbz', 'Bc', 'Bc1', 'Bca', 'Bcq', 'Bcv', 'Bcx', 'Bcz', 'Bd2',
'Bdu', 'Be1', 'Ben', 'Beq', 'Bfa', 'Bft', 'Bfu', 'Bg3', 'Bg4', 'Bg5',
'Bgx', 'Bhi', 'Bhy', 'Bi2', 'Bi8', 'Bjm', 'Bl1', 'Blz', 'Bm9', 'Bms',
'Bn1', 'Bn6', 'Bnh', 'Bni', 'Bnl', 'Boc', 'Boe', 'Bpa', 'Bpd', 'Bpj',
'Bpo', 'Brz', 'Bsc', 'Bse', 'Bsp', 'Bt1', 'Btp', 'Bua', 'Buj', 'Buk',
'Bw2', 'Bx3', 'Bza', 'Bzm', 'C15', 'C1a', 'C1d', 'C1n', 'C1x', 'C24',
'C28', 'C2d', 'C2l', 'C31', 'C35', 'C3x', 'C40', 'C4m', 'C4x', 'C5m',
'C5p', 'C5x', 'C61', 'C66', 'C80', 'Cal', 'Cax', 'Cb1', 'Cb2', 'Cba',
'Cc3', 'Ccb', 'Cci', 'Ccy', 'Cdh', 'Ce0', 'Ce9', 'Cem', 'Cf2', 'Cfx',
'Cfy', 'Cgf', 'Cgq', 'Chk', 'Chm', 'Cho', 'Cig', 'Cil', 'Cio', 'Ciu',
'Cki', 'Ckp', 'Cli', 'Clu', 'Clv', 'Cma', 'Cmb', 'Cmm', 'Cmr', 'Cmy',
'Cn2', 'Cna', 'Cnn', 'Cno', 'Cnt', 'Co4', 'Co6', 'Coa', 'Cog', 'Coo',
'Coq', 'Cp0', 'Cpf', 'Cpm', 'Cpn', 'Cpp', 'Cpv', 'Cpx', 'Cpz', 'Cqa',
'Cre', 'Crj', 'Crn', 'Crv', 'Crx', 'Cs5', 'Csc', 'Csh', 'Csy', 'Ctg',
'Ctr', 'Cua', 'Cxe', 'Cy7', 'Cyb', 'Cye', 'Cyj', 'Cza', 'Czb', 'Czi',
'Czn', 'Czp', 'D10', 'D18', 'D19', 'D20', 'D24', 'D2c', 'D35', 'D3f',
'D3p', 'D4n', 'D4p', 'D5g', 'D5m', 'D7g', 'Da2', 'Daf', 'Dag', 'Daj',
'Dap', 'Db1', 'Dbg', 'Dbu', 'Dch', 'Ddn', 'Ddx', 'Ded', 'Dey', 'Dfk',
'Dg2', 'Dga', 'Dgh', 'Dgs', 'Dgx', 'Dhc', 'Dhg', 'Dhj', 'Dhm', 'Di9',
'Dic', 'Did', 'Dih', 'Dkk', 'Dkt', 'Dmo', 'Dmu', 'Dn1', 'Dnt', 'Do8',
'Dop', 'Dp5', 'Dpb', 'Dpd', 'Dpf', 'Dpm', 'Dpy', 'Dr2', 'Dr4', 'Dr7',
'Dre', 'Drp', 'Drz', 'Dsb', 'Dsi', 'Dtc', 'Dtg', 'Duo', 'Dwz', 'Dxa',
'Dxb', 'E', 'E09', 'E1x', 'E6c', 'Eag', 'Ebg', 'Ed1', 'Ed2', 'Ede',
```

```
'Eeb', 'Efz', 'Eg1', 'Egc', 'Eh5', 'Eit', 'Ejt', 'Emf', 'Emp', 'Enb',
'Eob', 'Eod', 'Eom', 'Eoz', 'Epb', 'Epd', 'Epg', 'Epy', 'Ery', 'Esd',
'Esy', 'Etb', 'Eti', 'Ezr', 'F18', 'F24', 'F25', 'F2g', 'F2p', 'F3p',
'Fa2', 'Fcd', 'Fcg', 'Fcl', 'Fdf', 'Fex', 'Fg1', 'Fgp', 'Fhl', 'Fkp',
'Flg', 'Flh', 'Flq', 'Fmc', 'Fmd', 'Fmf', 'Fog', 'Fok', 'Foo', 'Fou',
'Fox', 'Fpb', 'Fpg', 'Frg', 'Frh', 'Frl', 'Fse', 'Fta', 'Fux', 'Fxi',
'G20', 'G21', 'G28', 'G2f', 'G2h', 'G34', 'G6a', 'G80', 'Ga0', 'Gac',
'Gb', 'Gbc', 'Gc4', 'Gc7', 'Gcd', 'Gck', 'Gda', 'Gdm', 'Gdn', 'Gds',
'Ge2', 'Gel', 'Get', 'Gfg', 'Gg4', 'Ggb', 'Ggp', 'Ggt', 'Ghr', 'Gil',
'Gk5', 'Gk6', 'Gl0', 'Gle', 'Glq', 'Gm1', 'Gmn', 'Gmu', 'Gna', 'Gp6',
'Gp9', 'Gpg', 'Gs2', 'Gs3', 'Gst', 'Gsw', 'Gtd', 'Gth', 'Gu1', 'Gu2',
'Gu8', 'Gu9', 'Gul', 'Gvd', 'Gvf', 'Gvs', 'Gw5', 'Gwb', 'H01', 'H1n',
'H2a', 'H4z', 'Had', 'Hc7', 'Hcg', 'Hcr', 'Hcx', 'Hdp', 'Heu', 'Hfp',
'Hfv', 'Hg9', 'Hgp', 'Hgu', 'Hgx', 'Hin', 'Hiq', 'Hit', 'Hj2', 'Hkv',
'Hm5', 'Hmb', 'Hmy', 'Ho2', 'Hob', 'Hol', 'Hoq', 'Hpq', 'Hqp', 'Hrg',
'Hri', 'Hrm', 'Hsa', 'Ht1', 'Ht2', 'Hts', 'Hud', 'Hup', 'Hxc', 'Hxp',
'Hxs', 'Hyg', 'Hzh', 'I20', 'I22', 'I52', 'I5c', 'Iam', 'Ibb', 'Ibs',
'Icu', 'Idt', 'Iet', 'Ifm', 'Igc', 'Ign', 'Ih1', 'Ih2', 'Ih6', 'Ihh',
'Ihi', 'Ihs', 'Iic', 'Ik2', 'Imb', 'Imk', 'Imm', 'In4', 'In5', 'Inn',
'Iom', 'Ipn', 'Ipu', 'Irf', 'Iss', 'Ist', 'Itl', 'Iu', 'Iyz', 'J12',
'J15', 'Jef', 'Js4', 'Js5', 'Jst', 'K01', 'K1r', 'K64', 'Kam', 'Ke4',
'Keu', 'Ki2', 'Kln', 'Kmp', 'Ks4', 'Kt3', 'Kt5', 'Ktp', 'L00', 'L0i',
'L11', 'L1p', 'L34', 'L37', 'L3g', 'L47', 'Lam', 'Lap', 'Las', 'Lc',
'Lcs', 'Ldp', 'Led', 'Leo', 'Let', 'Lg0', 'Lg2', 'Lgf', 'Lhu', 'Li0',
'Li1', 'Lk3', 'Lk5', 'Lk7', 'Llf', 'Lna', 'Lnb', 'Lng', 'Lnk', 'Lno',
'Lo1', 'Lpe', 'Lpr', 'Lps', 'Lso', 'Lux', 'Lvs', 'M09', 'M1c', 'M2p',
'M33', 'M54', 'M5m', 'M5s', 'Ma8', 'Maf', 'Mat', 'Mav', 'May', 'Mcr',
'Mdc', 'Mdp', 'Meb', 'Mei', 'Mel', 'Mfb', 'Mfx', 'Mg8', 'Mgu', 'Mia',
'Mip', 'Mit', 'Mji', 'Mlc', 'Mln', 'Mmc', 'Mmn', 'Mmo', 'Mmt', 'Mnu',
'Mod', 'Mon', 'Moq', 'Mot', 'Mp2', 'Mpa', 'Mpc', 'Mpg', 'Mpt', 'Mpu',
'Mq0', 'Mqd', 'Mrg', 'Msd', 'Msh', 'Msp', 'Mtk', 'Mtm', 'Mtr', 'Mub',
'Mul', 'Muu', 'Muv', 'Mvb', 'Mx1', 'Mxd', 'My1', 'Myc', 'Mye', 'N1h',
'N25', 'N2p', 'N4t', 'N5b', 'N6g', 'N8t', 'Na1', 'Na3', 'Nap', 'Naq',
'Nau', 'Nba', 'Nbu', 'Nch', 'Ncw', 'Nde', 'Ne6', 'Ner', 'Nf2', 'Nfp',
'Nfx', 'Ngp', 'Nhq', 'Nhs', 'Nin', 'Nip', 'Nir', 'Nlg', 'Nlx', 'Nma',
'Nms', 'Nmt', 'Nn2', 'Nn4', 'Noc', 'Nom', 'Noy', 'Np', 'Npc', 'Ns1',
'Nt2', 'Ntn', 'Ntu', 'Nvp', 'Nxd', 'Nyc', 'O2c', 'Oac', 'Oar', 'Obs',
'Oca', 'Ode', 'Oin', 'Oir', 'Ono', 'Opd', 'Opi', 'Ox5', 'Oxc', 'Oxi',
'Oxn', 'P0e', 'P17', 'P1c', 'P1h', 'P1o', 'P2t', 'P34', 'P38', 'P3g',
'P3p', 'P40', 'P6c', 'Pae', 'Pai', 'Pat', 'Paz', 'Pb1', 'Pbi', 'Pbt',
'Pc8', 'Pda', 'Pdi', 'Pdm', 'Pdu', 'Pdx', 'Pe1', 'Ped', 'Peo', 'Pet',
'Pev', 'Pez', 'Pfg', 'Pfz', 'Pg7', 'Pgk', 'Pgw', 'Ph3', 'Phj', 'Php',
'Phw', 'Pin', 'Pio', 'Piq', 'Pla', 'Plc', 'Plg', 'Pll', 'Plx', 'Pm2',
'Pm8', 'Pmt', 'Pmz', 'Pn1', 'Pne', 'Pnt', 'Po5', 'Pod', 'Pog', 'Poq',
'Pot', 'Pp3', 'Ppd', 'Ppg', 'Pph', 'Ppj', 'Pps', 'Ppu', 'Ppx', 'Pq1',
'Pqe', 'Pr5', 'Prb', 'Pre', 'Prt', 'Prz', 'Ps6', 'Psb', 'Psf', 'Psg',
'Psr', 'Pss', 'Ptc', 'Ptx', 'Pu', 'Pu5', 'Pvb', 'Pvc', 'Px4', 'Pxb',
'Pxt', 'Py4', 'Py5', 'Py6', 'Py8', 'Pyb', 'Pyp', 'Pzz', 'Qeh', 'Qin',
'Qlg', 'Qlp', 'Qpu', 'Qua', 'Que', 'R02', 'R12', 'R5p', 'R6g', 'R88',
'Raa', 'Rag', 'Ran', 'Rb2', 'Rb3', 'Rbe', 'Rby', 'Rc7', 'Rc8', 'Rdi',
'Ren', 'Rgs', 'Rh1', 'Rig', 'Ril', 'Ris', 'Rj6', 'Rmc', 'Rmp', 'Rng',
'Rno', 'Ro0', 'Rof', 'Rox', 'Rpp', 'Rpx', 'Rr1', 'Rrr', 'Rs3', 'Rso',
'Rup', 'Rx3', 'Rxc', 'Ryu', 'S02', 'S06', 'S13', 'S2m', 'S4c', 'S55',
'S91', 'Sad', 'Sb3', 'Sb6', 'Sbh', 'Sbx', 'Sc5', 'Sca', 'Sco', 'Sct',
'Scu', 'Scz', 'Sdt', 'Se0', 'Sfd', 'Sfm', 'Sft', 'Sgr', 'Sgx', 'Shd',
'Sie', 'Sil', 'Sio', 'Slf', 'Sma', 'Smp', 'Smt', 'Sn8', 'Sn9', 'Sna',
'Sno', 'So1', 'Soy', 'Spc', 'Spk', 'Spl', 'Spt', 'Sq', 'Sqd', 'Srl',
'Srs', 'Sry', 'Sso', 'Ssp', 'Ssu', 'St2', 'Ste', 'Sth', 'Stp', 'Su2',
'Sua', 'Sub', 'Suj', 'Sum', 'Suw', 'Svv', 'Svw', 'T2a', 'T2p', 'T2s',
'T2t', 'T36', 'T66', 'T6p', 'Ta4', 'Taa', 'Tam', 'Tao', 'Tat', 'Tax',
```

```
'Taz', 'Tb1', 'Tba', 'Tbc', 'Tbh', 'Tbt', 'Tbu', 'Tc1', 'Tch', 'Tcp',
'Tcr', 'Tea', 'Tee', 'Tem', 'Ter', 'Tfe', 'Tfo', 'Tft', 'Tgc', 'Tgf',
'Th1', 'Thg', 'Thn', 'Thu', 'Tl1', 'Tl2', 'Tl4', 'Tlb', 'Tlp', 'Tmc',
'Tmh', 'Tnv', 'Toe', 'Top', 'Toy', 'Tp7', 'Tpa', 'Tpc', 'Tpg', 'Tpl',
'Tpn', 'Tqd', 'Tqt', 'Trt', 'Ts2', 'Ts4', 'Tse', 'Ttd', 'Ttm', 'Tvn',
'Twn', 'Tx4', 'Txd', 'Txl', 'Txp', 'Tyc', 'Tyt', 'Tyz', 'Tza', 'Tzt',
'U01', 'U03', 'U16', 'U17', 'U1s', 'U21', 'U2l', 'U31', 'U34', 'U3h',
'U49', 'U8u', 'Ubb', 'Udc', 'Uiz', 'Un1', 'Un2', 'Un6', 'Uni', 'Up1',
'Upl', 'Upm', 'Uq', 'Uq8', 'Ur3', 'Urg', 'V5x', 'Var', 'Vaz', 'Vdl',
'Vll', 'Vol', 'Vrg', 'Vrv', 'Vs3', 'Vs4', 'Vsc', 'Vx', 'Vx6', 'Vzz',
'W05', 'W2x', 'Wrg', 'Wy2', 'Wz4', 'X7o', 'X9q', 'Xcy', 'Xdr', 'Xr2',
'Xs2', 'Xxx', 'Xyf', 'Y19', 'Y3', 'Yf3', 'Yf4', 'Z', 'Zap', 'Zba',
'Zcy', 'Zdu', 'Zeb', 'Zio', 'Zit', 'Zmo', 'Zmp', 'Zra', 'Zth', 'Zu5']
PDB Total: 6035 of 17900 (33.72%)
IDEAL Total: 7010 of 17900 (39.16%)
Total: 13045 of 17900 (72.88%)
----------------------------------------------------------------------
----------
----------------------------------------------------------------------
----------
& ACPYPE has a routine called 'guessCharge', derived from ANTECHAMBER,
based on gasteiger method, to try to guess the net charge of the
molecule.

*** For results [7], [8], [9], only guessCharge failed, but running
semi-QM with charge = 0 finished fine:

[7] Mols only guessCharge failed for both PDB and Ideal:
0     []

[8] Mols only guessCharge failed with PDB ONLY, besides [7]:
2     ['Cya', 'Cyo']

[9] Mols only guessCharge failed with IDEAL ONLY, besides [7]:
0     []
PDB Total: 2 of 17900 (0.01%)
IDEAL Total: 0 of 17900 (0.00%)
Total: 2 of 17900 (0.01%)
----------------------------------------------------------------------
----------
----------------------------------------------------------------------
----------
& ANTECHAMBER check, pair of atoms closer than expected.

*** For results [10], [11], [12], atoms in close contact:

[10] Mols have atoms in close contact for both PDB and Ideal:
48     ['13t', '1nh', '4hd', '7x3', 'Acd', 'Any', 'Bep', 'Bi6', 'Bii',
'Bij', 'Biv', 'Brx', 'C01', 'C44', 'Cai', 'Cm8', 'Coj', 'Cx1', 'D3e',
'Dmc', 'Dol', 'Ern', 'Eud', 'F22', 'Fa9', 'Far', 'Fd1', 'Frv', 'Fua',
'Iqa', 'Kab', 'Kgq', 'Lan', 'Lnl', 'Mm3', 'N2t', 'N5t', 'Nhn', 'Qfi',
'Sa3', 'Spn', 'Spr', 'Tn4', 'Uq6', 'Vg0', 'Vg4', 'Vg5', 'Vlb']

[11] Mols have atoms in close contact with PDB ONLY, besides [10]:
824     ['001', '009', '00a', '012', '01a', '061', '0as', '0jz',
'0ze', '10u', '11f', '127', '128', '12d', '12u', '130', '139', '152',
'166', '173', '19u', '1ac', '1by', '1jz', '1ma', '1nt', '1pn', '1tp',
'20e', '20s', '221', '241', '250', '255', '279', '285', '288', '292',
'297', '2aa', '2be', '2ed', '2fp', '2ha', '2ip', '2mu', '2my', '2pd',
'2rl', '2s3', '2tn', '300', '315', '336', '33p', '38m', '39z', '3am',
```

```
'3an', '3b3', '3bc', '3bi', '3bn', '3bt', '3mp', '3nh', '3od', '3pa',
'3pi', '433', '44d', '459', '4bg', '4cm', '4fr', '4ig', '4md', '4pn',
'4r8', '4tc', '529', '550', '585', '5as', '5cb', '5cp', '5fd', '5fr',
'5ha', '5he', '5ob', '5pa', '615', '642', '688', '6oc', '6pl', '715',
'741', '745', '760', '79z', '7ca', '7nh', '806', '81a', '846', '872',
'887', '888', '8gp', '8pc', '936', '965', '9in', 'A14', 'A16', 'A17',
'A26', 'A4c', 'A8b', 'Aag', 'Aai', 'Ab2', 'Abb', 'Acw', 'Acz', 'Adc',
'Add', 'Adj', 'Af', 'Af1', 'Af4', 'Ag2', 'Ag6', 'Agg', 'Aht', 'Ajh',
'Ajm', 'Aka', 'Akc', 'Akv', 'Aky', 'Al3', 'Alg', 'Alh', 'Alt', 'Am1',
'Amb', 'Amy', 'An1', 'Ao1', 'Ap0', 'Ap2', 'Apn', 'Aq4', 'Arc', 'Arh',
'Arr', 'Arx', 'As1', 'As4', 'Asf', 'Atp', 'Au4', 'Ax3', 'Axt', 'Axx',
'Azq', 'Azx', 'B08', 'B2s', 'B3p', 'B66', 'Ba1', 'Ba2', 'Bag', 'Bao',
'Bbz', 'Bcq', 'Bcv', 'Bcz', 'Bd2', 'Ben', 'Bft', 'Bfu', 'Bhi', 'Bhy',
'Bi2', 'Bjm', 'Bl1', 'Blz', 'Bm9', 'Bms', 'Bn1', 'Bn6', 'Bnh', 'Bni',
'Bnl', 'Bpd', 'Bpo', 'Brz', 'Bt1', 'Btp', 'Buj', 'Buk', 'Bx3', 'Bzm',
'C24', 'C28', 'C31', 'C35', 'C3x', 'C40', 'C4m', 'C4x', 'C5m', 'C5p',
'C5x', 'C61', 'C80', 'Cal', 'Cax', 'Cb1', 'Cba', 'Cbb', 'Cc3', 'Ccb',
'Ce0', 'Cem', 'Cgf', 'Chk', 'Cio', 'Cki', 'Cli', 'Clv', 'Cma', 'Cmb',
'Cmm', 'Cn2', 'Cnn', 'Cno', 'Co4', 'Coa', 'Cog', 'Coq', 'Cpf', 'Cpm',
'Cpp', 'Cpx', 'Cqa', 'Cre', 'Crj', 'Crn', 'Crv', 'Cs5', 'Csh', 'Csy',
'Ctr', 'Cy7', 'Cyb', 'Cye', 'Cza', 'Czb', 'Czi', 'Czn', 'D10', 'D18',
'D19', 'D20', 'D24', 'D2c', 'D35', 'D3f', 'D4n', 'D7g', 'Da2', 'Daf',
'Daj', 'Dap', 'Db1', 'Dbs', 'Ddn', 'Ded', 'Dey', 'Dfk', 'Dg2', 'Dhg',
'Dhj', 'Dhm', 'Di9', 'Did', 'Dkk', 'Dkt', 'Dlq', 'Dn1', 'Dnt', 'Do8',
'Dot', 'Dp5', 'Dpb', 'Dpd', 'Dpf', 'Dpm', 'Dr2', 'Dr4', 'Dr7', 'Dre',
'Dtc', 'Dwz', 'Dxa', 'Dxb', 'E', 'E09', 'E6c', 'Ebg', 'Ed1', 'Ed2',
'Ede', 'Efz', 'Eg1', 'Eh5', 'Ejt', 'Emf', 'Enb', 'Eob', 'Eod', 'Eoz',
'Epb', 'Epd', 'Epg', 'Epy', 'Ery', 'Esy', 'Etb', 'Eti', 'Etr', 'Ezr',
'F18', 'F24', 'F25', 'F2g', 'F2p', 'F3p', 'Faa', 'Fcg', 'Fdf', 'Fkp',
'Fmd', 'Fog', 'Fok', 'Foo', 'Fpb', 'Fpg', 'Fre', 'Frg', 'Frh', 'Frl',
'Fse', 'Fta', 'Fxi', 'G20', 'G21', 'G28', 'G2f', 'G2h', 'G34', 'G6a',
'G80', 'Gac', 'Gbc', 'Gc7', 'Gck', 'Gdm', 'Gds', 'Gel', 'Get', 'Gfg',
'Gg4', 'Ggb', 'Ggp', 'Ggt', 'Ghr', 'Gil', 'Gk5', 'Gk6', 'Gl0', 'Gle',
'Gm1', 'Gna', 'Gp6', 'Gp9', 'Gpg', 'Gs2', 'Gs3', 'Gst', 'Gsw', 'Gtd',
'Gth', 'Gvd', 'Gvf', 'Gvs', 'Gw5', 'Gwb', 'H01', 'Hc7', 'Hcg', 'Hcr',
'Hcx', 'Hfp', 'Hfv', 'Hgt', 'Hgu', 'Hj2', 'Hkv', 'Hm5', 'Hmb', 'Hmy',
'Ho2', 'Hqp', 'Hrg', 'Hri', 'Hrm', 'Hsa', 'Ht1', 'Ht2', 'Hud', 'Hup',
'Hxp', 'Hxs', 'Hyg', 'Hzh', 'I20', 'I22', 'I52', 'Ibs', 'Icu', 'Iet',
'Ifm', 'Ign', 'Ih1', 'Ih2', 'Ih6', 'Ihh', 'Iic', 'Ik2', 'Imb', 'Imm',
'In4', 'In5', 'Inn', 'Ipn', 'Ipu', 'Irf', 'Iss', 'Itl', 'Iyz', 'J15',
'Js4', 'Js5', 'K01', 'K64', 'Kam', 'Ke4', 'Keu', 'Kln', 'Kmp', 'Ks4',
'Ktp', 'L00', 'L0i', 'L11', 'L1p', 'L34', 'L37', 'L3g', 'L47', 'Las',
'Lcs', 'Ldp', 'Leo', 'Lg0', 'Lg2', 'Lgf', 'Li0', 'Lk3', 'Lk5', 'Lk7',
'Llf', 'Lna', 'Lnb', 'Lno', 'Lo1', 'Lpr', 'Lux', 'M1c', 'M2p', 'M33',
'M54', 'M5s', 'Ma8', 'Mbv', 'Mc9', 'Mdc', 'Meb', 'Mei', 'Mel', 'Mfx',
'Mgu', 'Mia', 'Mit', 'Miy', 'Mji', 'Mlc', 'Mmc', 'Mmn', 'Mmt', 'Mod',
'Mon', 'Moq', 'Mot', 'Mp2', 'Mpu', 'Mq0', 'Mq8', 'Mqd', 'Msd', 'Msf',
'Msh', 'Msp', 'Mtk', 'Mtm', 'Mub', 'Mul', 'Muu', 'Muv', 'Mvb', 'Mx1',
'Mxd', 'My1', 'Myc', 'N1h', 'N25', 'N2p', 'N4t', 'N5b', 'N8t', 'Na3',
'Nap', 'Nau', 'Nch', 'Ncw', 'Ne6', 'Nfp', 'Nfx', 'Ngp', 'Nir', 'Nlg',
'Nlx', 'Nma', 'Nn2', 'Nn4', 'Noc', 'Nod', 'Nom', 'Np', 'Npc', 'Ns1',
'Nvp', 'Nxd', 'Oac', 'Oar', 'Ode', 'Oir', 'Ono', 'Opi', 'Opt', 'Oxc',
'P1c', 'P1h', 'P1o', 'P34', 'P38', 'P3g', 'P3p', 'P40', 'P6c', 'P6l',
'Pae', 'Pai', 'Paz', 'Pb1', 'Pbi', 'Pc8', 'Pc9', 'Pck', 'Pda', 'Pdi',
'Pdm', 'Pdu', 'Peh', 'Peo', 'Pet', 'Pfz', 'Pgk', 'Pgw', 'Ph3', 'Ph9',
'Phj', 'Phw', 'Pin', 'Piq', 'Pla', 'Plc', 'Plg', 'Pll', 'Pm2', 'Pm8',
'Pmg', 'Pmt', 'Pmz', 'Pn1', 'Pne', 'Pnt', 'Po5', 'Pod', 'Poq', 'Pp3',
'Ppd', 'Ppg', 'Ppj', 'Ppu', 'Pqe', 'Prb', 'Prz', 'Ps2', 'Ps6', 'Psb',
'Psc', 'Psg', 'Pss', 'Ptn', 'Pu', 'Pu5', 'Px4', 'Pxb', 'Pxt', 'Py4',
'Py5', 'Py6', 'Py8', 'Pzz', 'Qin', 'Qlp', 'Qpu', 'Que', 'R02', 'R12',
```

```
'R5p', 'R6g', 'R88', 'Rag', 'Ran', 'Rbe', 'Rby', 'Rc8', 'Rdi', 'Ren',
'Rfp', 'Rgs', 'Rh1', 'Rif', 'Rig', 'Ris', 'Rj6', 'Rmc', 'Rno', 'Ro0',
'Rof', 'Rox', 'Rpp', 'Rpt', 'Rr1', 'Rs3', 'Rso', 'Rtl', 'Rup', 'Rx3',
'Rxc', 'Ryu', 'S02', 'S55', 'S91', 'Sad', 'Sb3', 'Sb6', 'Sbx', 'Sco',
'Sct', 'Scu', 'Scz', 'Sdt', 'Se0', 'Sfd', 'Sfm', 'Sft', 'Shd', 'Sie',
'Sio', 'Slf', 'Sma', 'Sn8', 'Sn9', 'Sno', 'Spc', 'Spf', 'Spk', 'Spl',
'Srl', 'Srs', 'Sry', 'Sse', 'Sso', 'St2', 'Ste', 'Sth', 'Su2', 'Sub',
'Sum', 'Svr', 'T2a', 'T2p', 'T6p', 'T87', 'Ta3', 'Tac', 'Tao', 'Tb1',
'Tbh', 'Tbu', 'Tch', 'Tcr', 'Tem', 'Ter', 'Tgc', 'Tgf', 'Thg', 'Thn',
'Thu', 'Tl1', 'Tl2', 'Tl4', 'Tlp', 'Tmc', 'Tmh', 'Tnc', 'Tnv', 'Top',
'Toy', 'Tp7', 'Tpa', 'Tpg', 'Tpl', 'Tqd', 'Tqt', 'Trt', 'Ts2', 'Ts4',
'Ttx', 'Tvn', 'Twn', 'Tx4', 'Txd', 'Txl', 'Txp', 'Tyc', 'Tza', 'Tzt',
'U01', 'U03', 'U16', 'U17', 'U1s', 'U21', 'U31', 'U49', 'Udc', 'Uir',
'Un1', 'Un6', 'Uni', 'Up1', 'Uq8', 'Urg', 'V5x', 'Var', 'Vaz', 'Vol',
'Vrg', 'Vrv', 'Vs3', 'Vs4', 'Vx6', 'Vzz', 'W05', 'W2x', 'Wrg', 'Wz4',
'X9q', 'Xdr', 'Xxx', 'Y19', 'Y3', 'Yf3', 'Yf4', 'Zap', 'Zba', 'Zeb',
'Zio', 'Zit', 'Zmo', 'Zmp', 'Zra', 'Zu5']

[12] Mols have atoms in close contact with IDEAL ONLY, besides [10]:
122     ['153', '1ah', '211', '219', '2nh', '346', '34p', '3mm',
'4mp', '698', '699', '821', '864', '896', '8mr', '991', '9ip', 'A1t',
'A3d', 'A70', 'A85', 'A9a', 'Abd', 'Aeb', 'Ah1', 'Anj', 'Bdc', 'Biz',
'Bmo', 'Bmv', 'Btb', 'C03', 'Cbn', 'Cef', 'Cty', 'Cw1', 'Cy6', 'Cyu',
'D91', 'Day', 'Ddd', 'Dgg', 'Djr', 'Dmp', 'Dpx', 'Drr', 'Dxt', 'Ed4',
'Ein', 'Epa', 'F2n', 'F42', 'Fk5', 'Fpf', 'Frp', 'Gan', 'Gpm', 'H8h',
'Hbc', 'Heh', 'Hxa', 'Hyf', 'Iav', 'Imy', 'Imz', 'Jno', 'Kar', 'L2g',
'L2p', 'L3p', 'L4p', 'Lax', 'Ln1', 'Mfd', 'Mgm', 'Mkk', 'Mkr', 'Moa',
'Msb', 'Mui', 'Mut', 'Na9', 'Ncg', 'Nov', 'Oi1', 'Osc', 'Ova', 'Ple',
'Pmd', 'Psn', 'Q2y', 'Q82', 'Qn3', 'Qum', 'R78', 'Rom', 'Ru6', 'Saz',
'Sii', 'Smd', 'Sms', 'Sn3', 'Sp2', 'Spo', 'Ta1', 'Tb9', 'Tbo', 'Tg1',
'Tm5', 'Tm6', 'Ton', 'Tsn', 'Ucb', 'Udm', 'Uq1', 'Uq2', 'Uq5', 'Uq7',
'Vd1', 'Vdz', 'Xcl', 'Xtl']
PDB Total: 872 of 17900 (4.87%)
IDEAL Total: 170 of 17900 (0.95%)
Total: 1042 of 17900 (5.82%)
----------------------------------------------------------------------
----------
----------------------------------------------------------------------
----------
& At least one bond whose values doesn't seem to be realistic.

*** For results [13], [14], [15], irregular bonds:

[13] Mols have irregular bonds for both PDB and Ideal:
0     []

[14] Mols have irregular bonds with PDB ONLY, besides [13]:
15     ['193', '6mc', '6mt', 'A2l', 'Ab7', 'B4p', 'Clu', 'Cus', 'D5g',
'Dbu', 'Iam', 'Mcr', 'P17', 'Taz', 'Tc1']

[15] Mols have irregular bonds with IDEAL ONLY, besides [13]:
4     ['3cy', '3lg', 'A19', 'Lnc']
PDB Total: 15 of 17900 (0.08%)
IDEAL Total: 4 of 17900 (0.02%)
Total: 19 of 17900 (0.11%)
----------------------------------------------------------------------
----------
----------------------------------------------------------------------
----------
```

```
*** For results [16], [17], [18], irregular bonds and atoms in close
contact:

[16] Mols have irregular bonds and atoms in close contact for both PDB
and Ideal:
0     []

[17] Mols have irregular bonds and atoms in close contact with PDB
ONLY, besides [16]:
20     ['1ar', '2gl', '5fc', '9ta', 'Bcx', 'Be1', 'Bfa', 'Bse', 'C1a',
'Cci', 'Ckp', 'Cpv', 'Czp', 'Gc4', 'Jst', 'Lch', 'Naq', 'Nhs', 'Pdx',
'Tee']

[18] Mols have irregular bonds and atoms in close contact with IDEAL
ONLY, besides [16]:
53     ['194', '195', '1c2', '1fl', '205', '2pr', '3ca', '4hg', '4qb',
'730', '8fg', 'A38', 'A43', 'Aa5', 'Acf', 'Azd', 'Bik', 'Bth', 'Cbb',
'Cg', 'Dbs', 'Dh3', 'Doi', 'Ent', 'F19', 'Faa', 'Fab', 'Fns', 'Gnd',
'Htc', 'Iad', 'Ilf', 'Ilh', 'Ios', 'Mi2', 'Ncn', 'Ndc', 'Neb', 'Nix',
'Nop', 'P0p', 'Ph9', 'Pmn', 'S1s', 'Sk2', 'Snd', 'T87', 'Thy', 'Ui1',
'Uir', 'Un7', 'Un9', 'Zid']
PDB Total: 20 of 17900 (0.11%)
IDEAL Total: 53 of 17900 (0.30%)
Total: 73 of 17900 (0.41%)
----------------------------------------------------------------------
----------
----------------------------------------------------------------------
----------
& Basically, ANTECHAMBER didn't guess some parameters. Topology here
is useless.

*** For results [19], [20], [21], couldn't determine all parameters:

[19] Mols have missing parameters for both PDB and Ideal:
3     ['4hm', 'P2c', 'Tox']

[20] Mols have missing parameters with PDB ONLY, besides [19]:
0     []

[21] Mols have missing parameters with IDEAL ONLY, besides [19]:
0     []
PDB Total: 3 of 17900 (0.02%)
IDEAL Total: 3 of 17900 (0.02%)
Total: 6 of 17900 (0.03%)
----------------------------------------------------------------------
----------
----------------------------------------------------------------------
----------

*** For results [22], [23], [24], missing parameters, irregular bonds,
maybe wrong atomtype and atoms in close contact:

[22] Mols have missing parameters, irregular bonds, maybe wrong
atomtype and atoms in close contact for both PDB and Ideal:
0     []

[23] Mols have missing parameters, irregular bonds, maybe wrong
atomtype and atoms in close contact with PDB ONLY, besides [22]:
0     []
```

```
[24] Mols have missing parameters, irregular bonds, maybe wrong
atomtype and atoms in close contact with IDEAL ONLY, besides [22]:
1     ['Tyo']
PDB Total: 0 of 17900 (0.00%)
IDEAL Total: 1 of 17900 (0.01%)
Total: 1 of 17900 (0.01%)
----------------------------------------------------------------------
----------
----------------------------------------------------------------------
----------
& ACPYPE failed in the very first stages, even before guessing net
charge.

*** For results [25], [26], [27], no 'tmp', ACPYPE did nothing at all:

[25] Mols have no 'tmp' for both PDB and Ideal:
140     ['11r', '188', '1pb', '1pc', '202', '25a', '2fh', '34b',
'39b', '39e', 'Aas', 'Ace', 'Afc', 'Ag1', 'Alb', 'Anz', 'Azi', 'B15',
'B17', 'B22', 'B30', 'B3c', 'B51', 'Bda', 'Be7', 'Bfc', 'Bnr', 'C1o',
'C2c', 'C2o', 'C36', 'C9p', 'Cb5', 'Cby', 'Cfn', 'Cfo', 'Ch2', 'Ch3',
'Clp', 'Clz', 'Cm1', 'Cm2', 'Cmo', 'Cn1', 'Cnb', 'Cnc', 'Cnf', 'Cob',
'Coy', 'Cul', 'Cuo', 'Dml', 'Dw1', 'Dw2', 'Dwc', 'E52', 'Eab', 'Emt',
'Epo', 'Eth', 'Eto', 'F3s', 'Fc3', 'Fco', 'Fea', 'Fes', 'Fmi', 'Fs2',
'G0b', 'Gbp', 'Gcr', 'Gip', 'Gm2', 'Gnb', 'Gso', 'Hco', 'Hdd', 'Hdm',
'Hf3', 'Hg2', 'Hgb', 'Hgi', 'Hph', 'Ht5', 'Ias', 'Ime', 'Kys', 'Lco',
'Lcp', 'Lpc', 'Lpf', 'Mce', 'Mtu', 'Nco', 'Nfc', 'Nfe', 'Nfo', 'Nfr',
'Nh', 'Nlh', 'Nlk', 'Ofo', 'Ohe', 'Ops', 'Oxa', 'P1p', 'Pc0', 'Pej',
'Pgm', 'Pii', 'Po3', 'Por', 'Pp7', 'Pp8', 'Ps5', 'R4a', 'R5a', 'R5b',
'R6a', 'R7u', 'R9a', 'Rep', 'Rtc', 'Sbz', 'Sf3', 'Sf4', 'Tbr', 'Tdl',
'Tkl', 'Tml', 'Ttr', 'Umq', 'Vea', 'Wcc', 'Xcc', 'Yok', 'Yol', 'Yom',
'Zab', 'Zrc']

[26] Mols have no 'tmp' with PDB ONLY, besides [25]:
62     ['1fh', '543', '6he', '749', '7he', 'Ac9', 'Asx', 'Bpt', 'Cch',
'Cfc', 'Cfm', 'Clf', 'Cln', 'Cpo', 'Cub', 'Cum', 'Cun', 'Ddh', 'Def',
'Dhe', 'Fc6', 'Fci', 'Fdc', 'Fdd', 'Fde', 'Fec', 'Fem', 'Fll', 'Fne',
'Fso', 'Fsx', 'Glx', 'Has', 'Hb1', 'Hc0', 'Hc1', 'Hcn', 'He6', 'Hea',
'Heb', 'Hec', 'Hem', 'Heo', 'Hev', 'Hf5', 'Hfm', 'Hif', 'Hkl', 'Hme',
'Imf', 'Iri', 'Jm1', 'Me3', 'Mtd', 'Nfs', 'Oc7', 'Oc8', 'Pfc', 'Phf',
'Ru7', 'Srm', 'Ver']

[27] Mols have no 'tmp' with IDEAL ONLY, besides [25]:
0     []
PDB Total: 202 of 17900 (1.13%)
IDEAL Total: 140 of 17900 (0.78%)
Total: 342 of 17900 (1.91%)
----------------------------------------------------------------------
----------
----------------------------------------------------------------------
----------
& Initial structures clearly wrong.

*** For results [28], [29], [30], atoms with same coordinates:

[28] Mols have duplicated coordinates for both PDB and Ideal:
12     ['2do', 'Apk', 'C2s', 'Dbz', 'Dxd', 'Dxn', 'G2s', 'Lta', 'Nzh',
'Pha', 'Phl', 'Scs']

[29] Mols have duplicated coordinates with PDB ONLY, besides [28]:
18     ['2bt', '5ic', 'A5m', 'Aep', 'Cad', 'Cb2', 'Dmo', 'Dti', 'Eys',
```

```
'Gte', 'Hit', 'Pat', 'Php', 'Plx', 'Pps', 'Tam', 'Tea', 'Xr2']

[30] Mols have duplicated coordinates with IDEAL ONLY, besides [28]:
65     ['03r', '175', '2aa', '2mc', '2sa', '3aa', '413', '4co', '5hu',
'6ca', 'Abt', 'Adj', 'Ai', 'Akp', 'Ap0', 'Av2', 'Bin', 'Brm', 'Bt5',
'C1b', 'C34', 'Cc0', 'Cuc', 'Cya', 'Cyo', 'Dot', 'Ea5', 'Ead', 'Fae',
'Fl1', 'Fre', 'Gtb', 'Ket', 'Lch', 'M6t', 'Mde', 'Meo', 'Mo1', 'Mo2',
'N1t', 'N3t', 'Nc', 'Ni1', 'Ni2', 'Nmx', 'Oad', 'Oc1', 'Ocn', 'Of1',
'Of2', 'Of3', 'Ome', 'Ona', 'P13', 'Pmg', 'Ppe', 'Pr3', 'Pyi', 'Sfo',
'Tdp', 'Thw', 'Tz5', 'Zn3', 'Zno', 'Zpp']
PDB Total: 30 of 17900 (0.17%)
IDEAL Total: 77 of 17900 (0.43%)
Total: 107 of 17900 (0.60%)
----------------------------------------------------------------------
----------
----------------------------------------------------------------------
----------
& The GAFF atomtype defined by Antechamber may not be correct.
& E.g. for '00g', WARNING: atom type of   C14 (cc) and   C15 (cc) may
be wrong

*** For results [31], [32], [33], maybe wrong atomtype:

[31] Mols with maybe wrong atomtype for both PDB and Ideal:
433     ['00g', '0co', '12c', '13a', '154', '163', '180', '1aa',
'1ce', '1ci', '1cp', '1mg', '1n1', '1sa', '222', '224', '24t', '278',
'290', '2c5', '2d7', '2dd', '2eg', '2eq', '2gp', '2mz', '2o7', '2ph',
'2th', '310', '312', '326', '327', '333', '337', '33a', '342', '357',
'35g', '3c3', '3fc', '3mn', '3th', '422', '43p', '448', '462', '4b3',
'4bc', '4bh', '4hi', '4mz', '4py', '4pz', '524', '527', '52p', '552',
'55f', '570', '5am', '5bn', '5cg', '5de', '5ds', '5gp', '5hi', '5nc',
'5sc', '5za', '618', '627', '628', '62a', '645', '654', '656', '663',
'680', '696', '6sc', '703', '720', '735', '762', '76a', '780', '783',
'796', '7de', '7dg', '7mg', '801', '802', '87y', '880', '894', '895',
'8cs', '8dg', '8hi', '902', '907', '93a', '950', '972', '973', '9dg',
'9di', '9hi', '9hx', 'A04', 'A18', 'A4p', 'A53', 'Aaz', 'Ac2', 'Adk',
'Afz', 'Ag0', 'Ail', 'Aim', 'Air', 'Ais', 'Al2', 'Al5', 'Al7', 'Al9',
'Am7', 'Amq', 'Amt', 'Amz', 'Ang', 'Anr', 'Ard', 'Att', 'Avp', 'Ax1',
'Axa', 'Ayg', 'Aza', 'Azm', 'Bat', 'Bdi', 'Bgf', 'Bhs', 'Bi4', 'Bmi',
'Bmu', 'Bpg', 'Brd', 'Brg', 'Btd', 'Bto', 'Btz', 'Bvg', 'Bzu', 'C0e',
'C1e', 'C21', 'C2f', 'C3o', 'C60', 'C62', 'C85', 'C94', 'Cag', 'Ccu',
'Cda', 'Cdd', 'Ced', 'Cf5', 'Cg2', 'Ch6', 'Chq', 'Cid', 'Ck3', 'Ckk',
'Cl3', 'Cls', 'Cqr', 'Cr2', 'Cr9', 'Cro', 'Crt', 'Ct7', 'Ct9', 'D05',
'D16', 'D1b', 'D1g', 'D25', 'D3', 'D3g', 'D42', 'D94', 'Dbq', 'Dbt',
'Ddg', 'Dfg', 'Dgi', 'Dgp', 'Dki', 'Dr8', 'Drl', 'Dt2', 'Dt4', 'Dt5',
'Dx4', 'Dx8', 'Dzo', 'Ecn', 'Eda', 'Enp', 'Ets', 'F10', 'F1g', 'F3f',
'Fih', 'Flu', 'Fmb', 'Foa', 'Fr3', 'Fr6', 'Fr8', 'Frb', 'Frk', 'Frs',
'Frx', 'Ftb', 'Fti', 'Fug', 'Fxa', 'G15', 'G1r', 'G31', 'G33', 'G48',
'G49', 'G4m', 'G4p', 'G5p', 'Ga2', 'Gao', 'Gav', 'Gdc', 'Gdd', 'Gdp',
'Gdx', 'Gff', 'Gfh', 'Gh3', 'Gk3', 'Gkd', 'Gnp', 'Gp5', 'Gpc', 'Gpn',
'Gs', 'Gsp', 'Gsr', 'Gsx', 'Gtz', 'Gun', 'Gvg', 'Gw4', 'Gys', 'H02',
'H33', 'Ha1', 'Ha3', 'Hdt', 'Hpa', 'Hpx', 'Hpz', 'Hql', 'Hr1', 'Hr2',
'Hrb', 'Hsm', 'Hub', 'I02', 'I19', 'I96', 'Iaa', 'Idc', 'Ide', 'Idp',
'Ifp', 'Iia', 'Imp', 'Imu', 'In6', 'In8', 'Inm', 'Inq', 'Irp', 'Isf',
'Jpr', 'K03', 'Kag', 'Kh1', 'Kiq', 'Ksl', 'Ktn', 'L0d', 'L10', 'L15',
'L5g', 'Lit', 'Lkg', 'Lom', 'Lug', 'Ly4', 'Lya', 'Lz9', 'Lzc', 'Lzd',
'M0n', 'M1g', 'M2g', 'M3c', 'M7g', 'Mdo', 'Mg1', 'Mib', 'Mim', 'Mmp',
'Moc', 'Mof', 'Mog', 'Mp9', 'Mpy', 'Msr', 'Mti', 'Muf', 'N1c', 'Ncf',
'Nn3', 'Np3', 'Nyg', 'Nzo', 'O62', 'Occ', 'Ocr', 'Olp', 'Onm', 'Opa',
'P', 'P1d', 'P1g', 'P29', 'P4o', 'P55', 'Pbg', 'Pbq', 'Pf3', 'Pfm',
```

```
'Pgd', 'Pgp', 'Phx', 'Pia', 'Pim', 'Pli', 'Ptb', 'Ptg', 'Pts', 'Pub',
'Pzn', 'Qei', 'Quo', 'R1t', 'Rdc', 'Riv', 'Rlp', 'Roi', 'Rpa', 'Rtp',
'Run', 'S14', 'Sae', 'Sb2', 'Sc3', 'Scb', 'Sce', 'Scf', 'Sd8', 'Sln',
'Snh', 'Sni', 'Sp3', 'Spa', 'Ssl', 'Sss', 'Stx', 'Su1', 'T15', 'T98',
'Tad', 'Tdt', 'Tfk', 'Tia', 'Tmi', 'Tpd', 'Tpf', 'Tsx', 'Ttc', 'Ttg',
'Tzy', 'U89', 'Uba', 'Uc1', 'Uc2', 'Ukc', 'Up3', 'Vc3', 'Vdy', 'Via',
'Vx2', 'W29', 'Wan', 'Wy4', 'Xaa', 'Xgu', 'Xlc', 'Xv6', 'Ye7', 'Yg',
'Yyg', 'Zaa', 'Zd6', 'Zgu', 'Zol']

[32] Mols with maybe wrong atomtype with PDB ONLY, besides [31]:
19     ['1a2', '3sp', '410', '486', '598', 'Coh', 'Ena', 'Heq', 'Hni',
'Ih4', 'Md1', 'Mtc', 'Ore', 'Pc3', 'Pid', 'Pni', 'Pp9', 'Rho', 'Spx']

[33] Mols with maybe wrong atomtype with IDEAL ONLY, besides [31]:
110     ['13z', '1pd', '1pr', '22b', '2bd', '2ma', '2py', '317',
'32t', '354', '3ms', '3pd', '517', '5af', '5bp', '5z5', '607', '681',
'6mi', '6nh', '737', '7gu', '847', '984', '995', 'Aax', 'Apj', 'B49',
'B96', 'Bgm', 'Bh1', 'Bi3', 'Bla', 'Bph', 'Brh', 'Ca4', 'Cep', 'Cgp',
'Ch7', 'Cr7', 'Crg', 'Cru', 'Crw', 'Cyc', 'D13', 'D34', 'Dbv', 'Deu',
'Dmy', 'Feg', 'Fp1', 'Frj', 'Fry', 'Fu2', 'G25', 'G21', 'G46', 'G98',
'Gk4', 'Gne', 'Gvc', 'H4m', 'Hha', 'Hmd', 'Hst', 'Iey', 'Igu', 'Imt',
'Inu', 'Kef', 'Kj2', 'Kwt', 'L79', 'Lcf', 'Lg', 'Lyb', 'M98', 'M99',
'Mc', 'Mfc', 'Mxl', 'N2g', 'Niu', 'Npr', 'Ns5', 'Omg', 'Pcg', 'Pcz',
'Peb', 'Pgn', 'Ppw', 'Pvn', 'Pz1', 'Rbt', 'Rem', 'Rg1', 'S6g', 'Sb5',
'Sp0', 'Std', 'Su9', 'T30', 'Tel', 'Tgp', 'Tx5', 'U02', 'U66', 'Xc2',
'Xxp', 'Xyg']
PDB Total: 452 of 17900 (2.53%)
IDEAL Total: 543 of 17900 (3.03%)
Total: 995 of 17900 (5.56%)
----------------------------------------------------------------------
----------
----------------------------------------------------------------------
----------

*** For results [34], [35], [36], maybe wrong atomtype and atoms in
close contact:

[34] Mols with maybe wrong atomtype and atoms in close contact for
both PDB and Ideal:
4     ['2az', 'Jnf', 'P04', 'Tyk']

[35] Mols with maybe wrong atomtype and atoms in close contact with
PDB ONLY, besides [34]:
70     ['13z', '1pd', '1pr', '2ma', '2py', '317', '3ms', '3on', '517',
'5af', '5bp', '5z5', '681', '6nh', '984', 'Aax', 'Apj', 'B49', 'B96',
'Bh1', 'Bi3', 'Bla', 'Bpb', 'Bph', 'Brh', 'Ca4', 'Cyc', 'D13', 'D34',
'Dbv', 'Dmy', 'Feg', 'Fp1', 'Frj', 'Fry', 'G98', 'Gk4', 'Gvc', 'Hha',
'Hmd', 'Hst', 'Inu', 'Kef', 'Kj2', 'M98', 'M99', 'Mc', 'Niu', 'Npr',
'Ns5', 'Omg', 'Oxr', 'Pcg', 'Peb', 'Poh', 'Pz1', 'Rbt', 'Rem', 'Rg1',
'Sb5', 'Sp0', 'Std', 'Su9', 'T30', 'Tel', 'Tx5', 'U02', 'U66', 'Xc2',
'Xxp']

[36] Mols with maybe wrong atomtype and atoms in close contact with
IDEAL ONLY, besides [34]:
11     ['1a2', '3sp', '410', '486', 'Ih4', 'Mfn', 'Ore', 'Pni', 'Rgc',
'Spx', 'Ula']
PDB Total: 74 of 17900 (0.41%)
IDEAL Total: 15 of 17900 (0.08%)
Total: 89 of 17900 (0.50%)
----------------------------------------------------------------------
```

```
----------
--------------------------------------------------------------------------------
----------

*** For results [37], [38], [39], irregular bonds, maybe wrong
atomtype and atoms in close contact:

[37] Mols with irregular bonds, maybe wrong atomtype and atoms in
close contact for both PDB and Ideal:
0     []

[38] Mols with irregular bonds, maybe wrong atomtype and atoms in
close contact with PDB ONLY, besides [37]:
0     []

[39] Mols with irregular bonds, maybe wrong atomtype and atoms in
close contact with IDEAL ONLY, besides [37]:
1     ['598']
PDB Total: 0 of 17900 (0.00%)
IDEAL Total: 1 of 17900 (0.01%)
Total: 1 of 17900 (0.01%)
--------------------------------------------------------------------------------
----------
--------------------------------------------------------------------------------
----------

*** For results [40], [41], [42], guessCharge failed and missing
parameters:

[40] Mols have guessCharge failed and missing parameters for both PDB
and Ideal:
5     ['Af3', 'Amm', 'Ceq', 'Crl', 'Dp4']

[41] Mols have guessCharge failed and missing parameters with PDB
ONLY, besides [40]:
0     []

[42] Mols have guessCharge failed and missing parameters with IDEAL
ONLY, besides [40]:
1     ['Sif']
PDB Total: 5 of 17900 (0.03%)
IDEAL Total: 6 of 17900 (0.03%)
Total: 11 of 17900 (0.06%)
--------------------------------------------------------------------------------
----------
--------------------------------------------------------------------------------
----------

*** For results [43], [44], [45], guessCharge failed, irregular bonds
and maybe wrong atomtype:

[43] Mols have guessCharge failed, irregular bonds and maybe wrong
atomtype for both PDB and Ideal:
0     []

[44] Mols have guessCharge failed, irregular bonds and maybe wrong
atomtype with PDB ONLY, besides [43]:
1     ['Kwt']

[45] Mols have guessCharge failed, irregular bonds and maybe wrong
```

```
atomtype with IDEAL ONLY, besides [43]:
0     []
PDB Total: 1 of 17900 (0.01%)
IDEAL Total: 0 of 17900 (0.00%)
Total: 1 of 17900 (0.01%)
--------------------------------------------------------------------------------
--------------------------------------------------------------------------------

*** For results [46], [47], [48], guessCharge failed, missing
parameters and atoms in close contact:

[46] Mols have guessCharge failed, missing parameters and atoms in
close contact for both PDB and Ideal:
0     []

[47] Mols have guessCharge failed, missing parameters and atoms in
close contact with PDB ONLY, besides [46]:
1     ['Sif']

[48] Mols have guessCharge failed, missing parameters and atoms in
close contact with IDEAL ONLY, besides [46]:
0     []
PDB Total: 1 of 17900 (0.01%)
IDEAL Total: 0 of 17900 (0.00%)
Total: 1 of 17900 (0.01%)
--------------------------------------------------------------------------------
--------------------------------------------------------------------------------
& ACPYPE check, at least a pair of atoms closer than 0.5 Angs.

*** For results [49], [50], [51], atoms too close:

[49] Mols have atoms too close for both PDB and Ideal:
0     []

[50] Mols have atoms too close with PDB ONLY, besides [49]:
86     ['1cx', '1pp', '270', '2ea', '339', '3af', '3cm', '3tp', '442',
'468', '5ad', '641', '744', 'Agt', 'Apx', 'As0', 'Ay1', 'B13', 'B3n',
'Bap', 'Bc1', 'Bdu', 'Bi8', 'Boz', 'Bpa', 'Bpj', 'Bua', 'Bza', 'C1d',
'C2d', 'Ccy', 'Cdn', 'Cfy', 'Cp0', 'Cpn', 'Dag', 'Dih', 'Dmu', 'Dos',
'Dsi', 'Duo', 'Eeb', 'Fex', 'Ge2', 'Gha', 'H1n', 'Hxc', 'Iap', 'Ibb',
'Iom', 'Ket', 'Lnc', 'Lnk', 'Los', 'Lps', 'Lru', 'M09', 'Maf', 'Mde',
'Mfd', 'Nba', 'Nbu', 'Pez', 'Pph', 'Ppx', 'Pre', 'Rb2', 'Rc7', 'Rfa',
'Rfb', 'Rpx', 'Rua', 'S06', 'S13', 'Sc5', 'So1', 'Sq', 'Taa', 'Tax',
'Tbt', 'Tcp', 'Tpn', 'Tyt', 'U3h', 'Un2', 'Zex']

[51] Mols have atoms too close with IDEAL ONLY, besides [49]:
10     ['2hp', '974', 'Cnd', 'E64', 'Lg8', 'Nrg', 'P3f', 'Pad', 'Pi',
'St6']
PDB Total: 86 of 17900 (0.48%)
IDEAL Total: 10 of 17900 (0.06%)
Total: 96 of 17900 (0.54%)
--------------------------------------------------------------------------------
--------------------------------------------------------------------------------
& ACPYPE check, at least one expected bond atom with no bond within
```

```
3.0 Angs.

*** For results [52], [53], [54], atoms too alone:

[52] Mols have atoms too alone for both PDB and Ideal:
0     []

[53] Mols have atoms too alone with PDB ONLY, besides [52]:
63     ['2fg', '2hr', '2hs', '2nt', '3de', '3fq', '4sc', '5aa', '8mg',
'979', 'Akl', 'Avn', 'Azt', 'B1m', 'Bg4', 'Bg5', 'Bsc', 'C1n', 'Cf2',
'Ciu', 'Cmy', 'Cpz', 'Cr8', 'Cua', 'Cuz', 'Eit', 'Fcd', 'Fg1', 'Flh',
'Fmf', 'Fux', 'Gmu', 'Gnd', 'Gul', 'Hin', 'May', 'Mh2', 'Mh3', 'Mln',
'Mpg', 'Ner', 'Nmt', 'Noy', 'Pcl', 'Pot', 'Pq1', 'Psr', 'Ptx', 'Qua',
'Qum', 'Rrr', 'S4c', 'Sbh', 'Sfo', 'Sgr', 'Sgx', 'Svv', 'Svw', 'Tcq',
'Vdl', 'Vll', 'Xcy', 'Xyf']

[54] Mols have atoms too alone with IDEAL ONLY, besides [52]:
5     ['B69', 'B70', 'Ium', 'Ncp', 'Spf']
PDB Total: 63 of 17900 (0.35%)
IDEAL Total: 5 of 17900 (0.03%)
Total: 68 of 17900 (0.38%)
--------------------------------------------------------------------------
----------
--------------------------------------------------------------------------
----------
& Essentially, the net charge guessed may be not be correct

*** For results [55], [56], [57], tleap failed:

[55] Mols have tleap failed for both PDB and Ideal:
255     ['090', '120', '121', '122', '123', '124', '132', '191',
'1mz', '218', '25t', '275', '286', '2dp', '2no', '2pb', '2pc', '2tp',
'32u', '364', '39a', '3ag', '3ap', '3dp', '3ma', '3mc', '3mt', '3pc',
'4ap', '4br', '4cr', '4nm', '514', '5cy', '623', '6ct', '6pe', '711',
'742', '7mp', '8id', '8pa', 'A1p', 'Abi', 'Aga', 'Agh', 'Ai3', 'Ap7',
'Ap8', 'B71', 'Bab', 'Bam', 'Bay', 'Bdm', 'Bea', 'Ber', 'Bmh', 'Bmz',
'Bp2', 'Br3', 'Br5', 'Brq', 'C49', 'Ccq', 'Cdc', 'Ce2', 'Co3', 'Cpl',
'Cpr', 'Cps', 'Cr5', 'Crk', 'Ct6', 'Ct8', 'Cvi', 'Cyn', 'D2x', 'D7k',
'Da5', 'Da6', 'Dau', 'Dax', 'Dcu', 'Ddq', 'Det', 'Dgd', 'Dim', 'Dlp',
'Dmi', 'Dmx', 'Dnr', 'Dpw', 'Dsc', 'Dt3', 'Edc', 'Eem', 'El', 'Enc',
'Eov', 'Esi', 'Esp', 'Esx', 'Et', 'Fag', 'Fm2', 'Fmu', 'Frn', 'Fsn',
'Ftp', 'Fxv', 'G16', 'G1g', 'G2g', 'Gap', 'Gdv', 'Gi1', 'Gi2', 'Gi3',
'Gi4', 'Gim', 'Gl8', 'Gom', 'Gsz', 'Gta', 'Gu0', 'Gu5', 'Gu6', 'H1s',
'H86', 'Ham', 'Haz', 'Hp1', 'Hso', 'Htl', 'Htp', 'Hyd', 'I48', 'Imd',
'L0g', 'L1b', 'Lda', 'Ldm', 'Lg1', 'M1a', 'M5g', 'Ma0', 'Ma7', 'Mbt',
'Mc3', 'Mes', 'Mg7', 'Mgo', 'Mgq', 'Mgr', 'Mlp', 'Mpi', 'Mpl', 'Mt1',
'Mvl', 'Mzm', 'Nak', 'Ndm', 'Nds', 'Ngo', 'Nh2', 'Nho', 'Nhv', 'Ni9',
'Nk1', 'Nk2', 'Nmn', 'Nnr', 'No', 'No2', 'No3', 'Nox', 'Npe', 'Npl',
'Npp', 'Nsn', 'Nts', 'Nvi', 'Nxx', 'Nyp', 'O16', 'Odp', 'Oh', 'Oxt',
'P2i', 'P42', 'P4p', 'Pa8', 'Paa', 'Pc5', 'Pc6', 'Pex', 'Pfs', 'Pg8',
'Pgi', 'Pgs', 'Pis', 'Piu', 'Pl1', 'Pnj', 'Po2', 'Pp2', 'Pp4', 'Pp5',
'Pp6', 'Prh', 'Prm', 'Ps1', 'Pt2', 'Pur', 'Px2', 'Px6', 'Px8', 'Qkh',
'Raf', 'Rfc', 'Sam', 'Sbo', 'Scn', 'Sfc', 'Sh4', 'Soh', 'T5x', 'T6f',
'Tby', 'Tdk', 'Teo', 'Tfl', 'Tmq', 'Tmt', 'Tmz', 'Tpm', 'Tpp', 'Tps',
'Trr', 'Tya', 'U1k', 'Vib', 'Vio', 'Vnp', 'Vxa', 'Wwf', 'X', 'X41',
'X81', 'Xmp', 'Xp4', 'Xpa', 'Xxy', 'Yrr', 'Zk9']

[56] Mols have tleap failed with PDB ONLY, besides [55]:
23     ['1pt', '2hp', '2pt', '3aa', '974', 'A71', 'A72', 'A8n', 'Alk',
'Bin', 'Cpt', 'Dnd', 'Dpi', 'Fae', 'Fs1', 'Lpt', 'Pc4', 'Ppe', 'Pyi',
```

```
'Tgl', 'Tpt', 'Tz4', 'Tz5']

[57] Mols have tleap failed with IDEAL ONLY, besides [55]:
26     ['3de', '3pe', '4sc', 'A4l', 'Aad', 'B12', 'Ce8', 'Cr8', 'Dti',
'Ggd', 'Gha', 'Gm3', 'Gsg', 'Gte', 'Ilt', 'Pc1', 'Pcf', 'Pcl', 'Pie',
'Re1', 'Sek', 'Tcq', 'Tnp', 'Van', 'Z5a', 'Zfb']
PDB Total: 278 of 17900 (1.55%)
IDEAL Total: 281 of 17900 (1.57%)
Total: 559 of 17900 (3.12%)
----------------------------------------------------------------------
----------
----------------------------------------------------------------------
----------

*** For results [58], [59], [60], tleap failed, maybe wrong atomtype:

[58] Mols have tleap failed and maybe wrong atomtype for both PDB and
Ideal:
18     ['2ap', '323', '3p4', '523', 'Apt', 'Bmg', 'C12', 'Ch', 'G7m',
'I11', 'Mgp', 'Ngd', 'Pbz', 'Rhq', 'Ros', 'Sts', 'T5c', 'Xx2']

[59] Mols have tleap failed and maybe wrong atomtype with PDB ONLY,
besides [58]:
4     ['Bht', 'Bpp', 'Gtg', 'Sxn']

[60] Mols have tleap failed and maybe wrong atomtype with IDEAL ONLY,
besides [58]:
1     ['F43']
PDB Total: 22 of 17900 (0.12%)
IDEAL Total: 19 of 17900 (0.11%)
Total: 41 of 17900 (0.23%)
----------------------------------------------------------------------
----------
----------------------------------------------------------------------
----------
& Took more than 10h to finish... hence aborted.

*** For results [61], [62], [63], semi-QM timeout:

[61] Mols have semi-QM timeout for both PDB and Ideal:
50     ['0cr', '0e2', '1cr', '232', 'A78', 'Blb', 'Bmy', 'By6', 'Ccg',
'Cdl', 'Deq', 'Dit', 'E55', 'Fld', 'Gmm', 'Hbp', 'Hi6', 'Hlo', 'M4o',
'Mrs', 'Mrt', 'Mya', 'Nhm', 'Nsh', 'Nto', 'Ntp', 'Obi', 'Opc', 'P11',
'P3a', 'Pax', 'Pc7', 'Pcj', 'Pcw', 'Pek', 'Peu', 'Pmy', 'Psp', 'Pty',
'Pvd', 'Rsd', 'Sfa', 'Sn6', 'Sn7', 'Tp8', 'Txx', 'U10', 'Vax', 'Xpx',
'Z23']

[62] Mols have semi-QM timeout with PDB ONLY, besides [61]:
161     ['23s', '2at', '2bu', '2df', '2dm', '2gt', '2ot', '32s',
'3da', '3ty', '42b', '46d', '4ac', '4f3', '4pc', '4pd', '4pe', '4ta',
'5bu', '5pc', '64t', '6ma', '6og', '7da', 'A2g', 'A2m', 'A3a', 'A66',
'Aal', 'Ad2', 'Adu', 'B12', 'Bbb', 'Bg3', 'Bgx', 'Boe', 'C1x', 'C2l',
'C66', 'Ce8', 'Cgq', 'Cmr', 'Cnt', 'Crx', 'Csl', 'Cty', 'Cyj', 'D3p',
'D4p', 'Ddx', 'Dgh', 'Dgs', 'Dhc', 'Dpy', 'Drp', 'Drz', 'Dtg', 'E1x',
'Emp', 'Esd', 'Fa2', 'Fcl', 'Fgp', 'Fhl', 'Fox', 'Gcd', 'Gda', 'Gms',
'Gu1', 'Gu2', 'Gu8', 'Gu9', 'Had', 'Hdp', 'Heu', 'Hg9', 'Hiq', 'Hob',
'Hol', 'Hoq', 'I5c', 'Idt', 'Imk', 'Ist', 'Iu', 'K1r', 'Ki2', 'Lc',
'Led', 'Let', 'Lhu', 'Lng', 'Lso', 'M5m', 'Mat', 'Mav', 'Mfb', 'Mmo',
'Mnu', 'Mrg', 'Mtr', 'Mye', 'N6g', 'Na1', 'Nde', 'Nf2', 'Nhq', 'Nms',
'Ntn', 'O2c', 'Obs', 'Oca', 'Oin', 'Oxi', 'P2t', 'Pbt', 'Pev', 'Pg7',
```

```
'Pr5', 'Prt', 'Ptc', 'Pyb', 'Q2y', 'Raa', 'Rb3', 'Ril', 'Ru6', 'S2m',
'Sca', 'Sil', 'Smp', 'Soy', 'Ssu', 'Suj', 'Suw', 'Swi', 'T2s', 'T2t',
'T36', 'T66', 'Ta4', 'Tfe', 'Tfo', 'Tft', 'Th1', 'Tlb', 'Ttd', 'Ttm',
'Tyz', 'U2l', 'U34', 'U8u', 'Ubb', 'Ucb', 'Ums', 'Ur3', 'Van', 'Wy2',
'X7o', 'Z', 'Zdu']

[63] Mols have semi-QM timeout with IDEAL ONLY, besides [61]:
36     ['15p', 'B1m', 'Bah', 'Bv1', 'Bv2', 'Bv3', 'Bv4', 'Cdb', 'Cdn',
'Dpi', 'Dr6', 'Eli', 'Hxl', 'M0e', 'Mq8', 'Mq9', 'Mrr', 'Nf', 'Nt1',
'Opt', 'Otp', 'P6l', 'Pc9', 'Pck', 'Pee', 'Peh', 'Pew', 'Ph1', 'Pl9',
'Pld', 'Ps2', 'Psc', 'Py3', 'Rif', 'Tgl', 'Th3']
PDB Total: 211 of 17900 (1.18%)
IDEAL Total: 86 of 17900 (0.48%)
Total: 297 of 17900 (1.66%)
----------------------------------------------------------------------
----------
----------------------------------------------------------------------
----------

*** For results [64], [65], [66], semi-QM timeout and maybe wrong
atomtype:

[64] Mols have semi-QM timeout and maybe wrong atomtype for both PDB
and Ideal:
7     ['Blm', 'Pcu', 'Pho', 'Rga', 'Rh9', 'Sfp', 'Tot']

[65] Mols have semi-QM timeout and maybe wrong atomtype with PDB ONLY,
besides [64]:
18     ['2bd', '32t', '607', '7gu', '847', 'Ch7', 'Cr7', 'Cru', 'Crw',
'G25', 'G2l', 'Gne', 'Igu', 'Imt', 'Lg', 'Mfc', 'Pcz', 'Ppw']

[66] Mols have semi-QM timeout and maybe wrong atomtype with IDEAL
ONLY, besides [64]:
1     ['Sxn']
PDB Total: 25 of 17900 (0.14%)
IDEAL Total: 8 of 17900 (0.04%)
Total: 33 of 17900 (0.18%)
----------------------------------------------------------------------
----------
----------------------------------------------------------------------
----------

*** For results [67], [68], [69], guessCharge and tleap failed:

[67] Mols have guessCharge and tleap failed for both PDB and Ideal:
188     ['0a2', '0ay', '0bd', '105', '2bh', '2bl', '2mo', '368',
'3co', '3ni', '412', '427', '4cb', '4mo', '6mo', '6wo', 'A48', 'Ab5',
'Abh', 'Ad9', 'Adw', 'Ag', 'Ai2', 'Al', 'Amw', 'Apb', 'Apw', 'Ar',
'Ars', 'Asr', 'Au', 'Au3', 'Auc', 'B2a', 'B2f', 'B2i', 'B2v', 'Ba',
'Bdb', 'Be8', 'Bf2', 'Bjh', 'Bji', 'Bjp', 'Ble', 'Bly', 'Bno', 'Bo2',
'Bo3', 'Bo4', 'Bo9', 'Bor', 'Bpr', 'Br', 'Bub', 'Bzb', 'C08', 'Ca',
'Cb4', 'Cd', 'Ce', 'Cfq', 'Cl', 'Co', 'Cr', 'Cs', 'Ctb', 'Cu', 'Cu1',
'Cu3', 'Cvb', 'Daa', 'Daz', 'Di2', 'Di3', 'Di4', 'Di5', 'Doz', 'Dp7',
'Drb', 'Dru', 'Dtz', 'Dyb', 'Er3', 'Eu', 'Eu3', 'F', 'Fe', 'Fe2',
'G47', 'Ga', 'Gd', 'Gd3', 'Hdb', 'Hg', 'Ho', 'Ho3', 'In', 'Iod', 'Ir',
'Ir3', 'Itm', 'K', 'Kr', 'La', 'Li', 'Lrb', 'Lu', 'M1n', 'Map', 'Mf4',
'Mg', 'Mgf', 'Mm4', 'Mn', 'Mn3', 'Mo', 'Mos', 'Mow', 'Na', 'Nbf',
'Ndt', 'Ni', 'Nmq', 'Nob', 'Npb', 'O', 'Os', 'Os4', 'Pa0', 'Pb',
'Pba', 'Pbc', 'Pd', 'Pdv', 'Ppb', 'Pr', 'Pt', 'Pt4', 'Qtr', 'Rb',
'Rbu', 'Re', 'Reo', 'Rhm', 'Ru', 'S', 'S2c', 'Sb', 'Sbe', 'Sbp', 'Se',
```

```
'Sm', 'Sm3', 'Smo', 'Sr', 'Srb', 'Srd', 'T16', 'T29', 'T42', 'Tas',
'Tb', 'Tbd', 'Te', 'Tl', 'U1', 'Uvc', 'V', 'V36', 'V7o', 'Va3', 'Vg1',
'Vn4', 'Vo3', 'Vo4', 'W', 'Wo3', 'Wo4', 'Wo5', 'Wo6', 'Xe', 'Y1',
'Yb', 'Yb2', 'Yt3', 'Zh3', 'Zn']

[68] Mols have guessCharge and tleap failed with PDB ONLY, besides
[67]:
51     ['2db', 'Abt', 'Aio', 'Alf', 'Art', 'Ast', 'Ats', 'Av2', 'Avc',
'B69', 'B70', 'Baz', 'Bef', 'Bf4', 'Bva', 'Cac', 'Dae', 'Daq', 'Dvt',
'Fel', 'I42', 'Ica', 'Keg', 'Mnh', 'Mnq', 'Mo7', 'Mom', 'Moo', 'Mss',
'Myq', 'Nru', 'Oec', 'Omo', 'Onp', 'Pcd', 'Pnq', 'Pte', 'Req', 'Rhd',
'Rhx', 'Rta', 'Rtb', 'Sm4', 'T1a', 'Tsd', 'Tta', 'Unx', 'Va1', 'Wo2',
'Ybt', 'Zem']

[69] Mols have guessCharge and tleap failed with IDEAL ONLY, besides
[67]:
12     ['Boz', 'Cad', 'Iap', 'Jh0', 'Lru', 'Mh2', 'Rfa', 'Rfb', 'Rua',
'Tto', 'V35', 'V4o']
PDB Total: 239 of 17900 (1.34%)
IDEAL Total: 200 of 17900 (1.12%)
Total: 439 of 17900 (2.45%)
----------------------------------------------------------------------
----------
----------------------------------------------------------------------
----------

*** For results [70], [71], [72], guessCharge and tleap failed, maybe
wrong atomtype:

[70] Mols have guessCharge and tleap failed, maybe wrong atomtype for
both PDB and Ideal:
8     ['Bcl', 'Cxb', 'Etp', 'Mtq', 'Mtv', 'Sm2', 'T19', 'Tdb']

[71] Mols have guessCharge and tleap failed, maybe wrong atomtype with
PDB ONLY, besides [70]:
11     ['Bcb', 'Chl', 'Cl1', 'Cla', 'He5', 'Heg', 'Hes', 'Mnr', 'Ptt',
'R1c', 'Znh']

[72] Mols have guessCharge and tleap failed, maybe wrong atomtype with
IDEAL ONLY, besides [70]:
0     []
PDB Total: 19 of 17900 (0.11%)
IDEAL Total: 8 of 17900 (0.04%)
Total: 27 of 17900 (0.15%)
----------------------------------------------------------------------
----------
----------------------------------------------------------------------
----------

*** For results [73], [74], [75], atoms with same coordinates and
maybe wrong atomtype:

[73] Mols have atoms with same coordinates and maybe wrong atomtype
for both PDB and Ideal:
0     []

[74] Mols have atoms with same coordinates and maybe wrong atomtype
with PDB ONLY, besides [73]:
4     ['Bgm', 'Deu', 'Pgn', 'Rgc']
```

```
[75] Mols have atoms with same coordinates and maybe wrong atomtype
with IDEAL ONLY, besides [73]:
5     ['Bht', 'Ena', 'Gtg', 'Ptt', 'Rho']
PDB Total: 4 of 17900 (0.02%)
IDEAL Total: 5 of 17900 (0.03%)
Total: 9 of 17900 (0.05%)
----------------------------------------------------------------------
----------
----------------------------------------------------------------------
----------

*** For results [76], [77], [78], atoms too close and maybe wrong
atomtype:

[76] Mols have atoms too close and maybe wrong atomtype for both PDB
and Ideal:
0     []

[77] Mols have atoms too close and maybe wrong atomtype with PDB ONLY,
besides [76]:
6     ['354', '6mi', '995', 'F43', 'G46', 'L79']

[78] Mols have atoms too close and maybe wrong atomtype with IDEAL
ONLY, besides [76]:
1     ['Afg']
PDB Total: 6 of 17900 (0.03%)
IDEAL Total: 1 of 17900 (0.01%)
Total: 7 of 17900 (0.04%)
----------------------------------------------------------------------
----------
----------------------------------------------------------------------
----------

*** For results [79], [80], [81], atoms too alone and maybe wrong
atomtype:

[79] Mols have atoms too alone and maybe wrong atomtype for both PDB
and Ideal:
0     []

[80] Mols have atoms too alone and maybe wrong atomtype with PDB ONLY,
besides [79]:
4     ['3pd', 'Fu2', 'Lcf', 'Tyo']

[81] Mols have atoms too alone and maybe wrong atomtype with IDEAL
ONLY, besides [79]:
0     []
PDB Total: 4 of 17900 (0.02%)
IDEAL Total: 0 of 17900 (0.00%)
Total: 4 of 17900 (0.02%)
----------------------------------------------------------------------
----------
----------------------------------------------------------------------
----------

*** For results [82], [83], [84], atoms with same coordinates and too
close:

[82] Mols have atoms with same coordinates and too close for both PDB
and Ideal:
```

```
0     []

[83] Mols have atoms with same coordinates and too close with PDB
ONLY, besides [82]:
3     ['175', 'Nt2', 'Pr3']

[84] Mols have atoms with same coordinates and too close with IDEAL
ONLY, besides [82]:
25     ['1cu', '2of', '44c', 'A8n', 'Cec', 'Co5', 'Con', 'Dnd', 'Fel',
'Lol', 'Mm1', 'Mo3', 'Mo4', 'Mo5', 'Mo6', 'Mw1', 'Ni3', 'Nik', 'Nru',
'Ocl', 'Ocm', 'Oco', 'Rhd', 'Tz4', 'Zo3']
PDB Total: 3 of 17900 (0.02%)
IDEAL Total: 25 of 17900 (0.14%)
Total: 28 of 17900 (0.16%)
----------------------------------------------------------------------
----------
----------------------------------------------------------------------
----------

*** For results [85], [86], [87], atoms with same coordinates and too
alone:

[85] Mols have atoms with same coordinates and too alone for both PDB
and Ideal:
1     ['Mm2']

[86] Mols have atoms with same coordinates and too alone with PDB
ONLY, besides [85]:
212     ['14w', '1cu', '220', '2au', '2of', '367', '3pe', '3ph',
'418', '4nd', '4oc', '4pt', '5fu', '5hu', '5op', '6ia', '7ad', '826',
'8ad', 'A4l', 'Aad', 'Abl', 'Ads', 'Aip', 'An9', 'Ary', 'B9a', 'Ban',
'Bc', 'Bca', 'Bdc', 'Beq', 'Bmo', 'Boc', 'Brm', 'Bsp', 'Bt5', 'Bv1',
'Bv2', 'Bv3', 'Bv4', 'Bw2', 'C15', 'C34', 'Cd1', 'Cd3', 'Cd5', 'Cdh',
'Ce9', 'Cec', 'Cef', 'Cfx', 'Cho', 'Cil', 'Cna', 'Co5', 'Co6', 'Coo',
'Csc', 'Ctg', 'Cuc', 'Cxe', 'Cyu', 'D5m', 'D91', 'Dbg', 'Dch', 'Dga',
'Dgg', 'Dgx', 'Dic', 'Dr6', 'Dsb', 'Eag', 'Egc', 'Flg', 'Flq', 'Fmc',
'Ga0', 'Gb', 'Gdn', 'Gm3', 'Gmn', 'Gsg', 'H2a', 'H4z', 'Hgp', 'Hgx',
'Hpq', 'Igc', 'Ihi', 'Ihs', 'Imy', 'J12', 'Jef', 'Jh0', 'Ko4', 'Kt3',
'Kt5', 'L2p', 'L3p', 'L4p', 'Lam', 'Lap', 'Li1', 'Lol', 'Lpe', 'Lvs',
'M0e', 'Mdp', 'Mg8', 'Mip', 'Mm5', 'Mn5', 'Mn6', 'Mo1', 'Mo2', 'Mo3',
'Mo4', 'Mo5', 'Mo6', 'Mpa', 'Mpc', 'Mpt', 'Mrr', 'Mw1', 'Mw2', 'Mw3',
'Na2', 'Na5', 'Na6', 'Nao', 'Naw', 'Nc', 'Ncp', 'Ni1', 'Ni2', 'Ni3',
'Nik', 'Nip', 'Nt1', 'Ntu', 'Nyc', 'O4m', 'Oc1', 'Oc2', 'Oc3', 'Oc4',
'Oc5', 'Oc6', 'Ocl', 'Ocm', 'Ocn', 'Oco', 'Of1', 'Of2', 'Of3', 'Ome',
'Opd', 'Ox5', 'Oxn', 'P0e', 'Pc1', 'Pcf', 'Ped', 'Pee', 'Pfg', 'Pie',
'Pio', 'Pl9', 'Pld', 'Pog', 'Psf', 'Pvb', 'Pvc', 'Pyp', 'Qeh', 'Qlg',
'Re1', 'Rmp', 'Rng', 'Sek', 'Smt', 'Sna', 'Spt', 'Sqd', 'Ssp', 'Stp',
'Sua', 'Tba', 'Tbc', 'Th3', 'Tpc', 'Tse', 'Tto', 'Uiz', 'Upl', 'Upm',
'Uq', 'V35', 'V4o', 'Vsc', 'Vx', 'Xs2', 'Z5a', 'Zcy', 'Zfb', 'Zn3',
'Zno', 'Zo3', 'Zpp', 'Zth']

[87] Mols have atoms with same coordinates and too alone with IDEAL
ONLY, besides [85]:
5     ['Ca3', 'Ca5', 'Cok', 'Pte', 'Svr']
PDB Total: 213 of 17900 (1.19%)
IDEAL Total: 6 of 17900 (0.03%)
Total: 219 of 17900 (1.22%)
----------------------------------------------------------------------
----------
----------------------------------------------------------------------
```

*** For results [88], [89], [90], atoms with same coordinates, too alone and maybe wrong atomtype:

[88] Mols have atoms with same coordinates, too alone and maybe wrong atomtype for both PDB and Ideal:
0   []

[89] Mols have atoms with same coordinates, too alone and maybe wrong atomtype with PDB ONLY, besides [88]:
16   ['22b', '737', 'Cep', 'Cgp', 'Crg', 'H4m', 'Iey', 'Lyb', 'Mfn', 'Mxl', 'N2g', 'Pvn', 'S6g', 'Tgp', 'Ula', 'Xyg']

[90] Mols have atoms with same coordinates, too alone and maybe wrong atomtype with IDEAL ONLY, besides [88]:
0   []
PDB Total: 16 of 17900 (0.09%)
IDEAL Total: 0 of 17900 (0.00%)
Total: 16 of 17900 (0.09%)

*** For results [91], [92], [93], atoms with same coordinates, too close and too alone:

[91] Mols have atoms with same coordinates, too close and too alone for both PDB and Ideal:
0   []

[92] Mols have atoms with same coordinates, too close and too alone with PDB ONLY, besides [91]:
2   ['15p', 'Ggd']

[93] Mols have atoms with same coordinates, too close and too alone with IDEAL ONLY, besides [91]:
1   ['Ko4']
PDB Total: 2 of 17900 (0.01%)
IDEAL Total: 1 of 17900 (0.01%)
Total: 3 of 17900 (0.02%)
----
318 + 13045 + 2 + 1042 + 19 + 73 + 6 + 1 + 342 + 107 + 995 + 89 + 1 + 11 + 1 + 1 + 96 + 68 + 559 + 41 + 297 + 33 + 439 + 27 + 9 + 7 + 4 + 28 + 219 + 16 + 3 = 17899

& values above are for checking... the total number of grouped jobs should be 17900. The diff is due to the 'Ium_pdb' case referred in the beginning, which was not grouped.

& Below: e.g. "2pb_-2_-1" means mol '2pb' has net charge '-2' in mol2 (from Ccp) but ACPYPE guessed charge '-1' and used it for calculations.

>>> Mols with MOL2 charge different from ACPYPE guessed charge <<<

78 ['2pb_-2_-1', '2pc_0_-1', '2pt_3_2', '323_0_-1', '3ag_-3_-2', '3dp_0_1', '3me_0_-1', '3pc_0_1', '3pe_0_1', 'A71_0_4', 'A72_0_4',

```
'A8n_2_1', 'Ahz_-3_-1', 'Bdm_-1_-2', 'Bi7_0_1', 'Bmg_0_-1', 'Bmh_0_-1', 'Bp2_-3_-2', 'Ccq_0_1', 'Cdc_0_1', 'Cgq_3_1', 'Co3_-2_-1',
'Con_3_4', 'Cpl_0_1', 'Cps_0_1', 'Cpt_0_2', 'Da5_2_1', 'Da6_2_1',
'Dax_2_1', 'Dcu_2_1', 'Ddq_0_1', 'Det_0_1', 'Dlp_0_1', 'Dmx_0_1',
'Dpw_0_1', 'F43_0_1', 'Fld_4_2', 'Ftp_-1_-2', 'Gap_0_1', 'Gsg_0_1',
'H1s_-4_-1', 'H3s_-3_-1', 'Ham_0_1', 'Haz_0_1', 'Hmg_-5_-3',
'Hni_0_1', 'Ilt_2_1', 'L0g_2_1', 'Lda_0_1', 'Ldm_0_1', 'Mc3_0_1',
'Mes_0_1', 'Mm1_0_4', 'Mm6_0_1', 'Mp1_0_1', 'Nak_0_-1', 'Nds_0_1',
'Nox_0_1', 'Nsn_0_-1', 'P42_0_1', 'Pc1_0_1', 'Pc5_0_1', 'Pc6_0_1',
'Pcf_0_1', 'Pfs_0_1', 'Pni_0_1', 'Prm_2_1', 'Prt_-6_-4', 'Ptn_2_3',
'Sam_0_-1', 'Tcn_-2_1', 'Tcq_0_-1', 'Tdt_-4_-2', 'Teo_-2_-1',
'Tot_4_2', 'U1k_0_1', 'Xr2_4_2', 'Zk9_2_1']


& Below: jobs that would have failed henceforth since SQM developers
changed the code to abort calculations if in any step a 'No
convergence in SCF' appears.
& For the calculations done here this patch was not applied.

>>>Mol Jobs whose sqm.out has 'No convergence in SCF'<<<

779 ['01k_ideal', '01k_pdb', '10a_ideal', '118_pdb', '12p_ideal',
'141_ideal', '15m_pdb', '15p_ideal', '16p_ideal', '193_pdb',
'198_ideal', '1by_pdb', '1un_ideal', '205_ideal', '232_ideal',
'232_pdb', '23s_pdb', '2at_pdb', '2ba_pdb', '2bc_pdb', '2bd_pdb',
'2bu_pdb', '2df_pdb', '2dm_pdb', '2dp_ideal', '2dp_pdb', '2gt_pdb',
'2nc_ideal', '2nc_pdb', '2ot_pdb', '2pe_ideal', '2sp_ideal',
'312_ideal', '312_pdb', '326_pdb', '32s_pdb', '32t_pdb', '34d_ideal',
'3an_ideal', '3bi_ideal', '3cp_pdb', '3da_pdb', '3ep_ideal',
'3lg_ideal', '3ph_ideal', '3ty_pdb', '42b_pdb', '46d_pdb', '471_pdb',
'4ac_pdb', '4f3_pdb', '4hg_ideal', '4pc_pdb', '4pd_pdb', '4pe_pdb',
'4pt_ideal', '4su_ideal', '4su_pdb', '4ta_pdb', '4tc_ideal',
'4tc_pdb', '515_pdb', '587_ideal', '5am_ideal', '5bu_ideal',
'5bu_pdb', '5fc_pdb', '5fu_ideal', '5iu_ideal', '5pc_pdb',
'607_ideal', '607_pdb', '64t_pdb', '693_ideal', '6ma_pdb', '6mc_pdb',
'6mt_pdb', '6og_pdb', '6pl_ideal', '6ul_ideal', '6ul_pdb', '70u_pdb',
'730_ideal', '750_pdb', '757_ideal', '773_ideal', '784_ideal',
'7da_pdb', '7gu_pdb', '7ni_ideal', '7ph_ideal', '847_ideal',
'847_pdb', '852_ideal', '858_pdb', '8pp_pdb', '93a_ideal', '93a_pdb',
'941_pdb', 'A04_ideal', 'A1r_ideal', 'A2g_pdb', 'A2m_pdb', 'A2p_pdb',
'A2r_ideal', 'A2t_ideal', 'A3a_pdb', 'A3p_ideal', 'A4c_pdb',
'A4p_ideal', 'A4p_pdb', 'A66_pdb', 'A6p_pdb', 'A76_ideal',
'A78_ideal', 'A78_pdb', 'A79_pdb', 'A85_pdb', 'A9a_ideal', 'A9a_pdb',
'Aa7_ideal', 'Aa7_pdb', 'Aal_pdb', 'Aao_ideal', 'Ab7_pdb', 'Abf_pdb',
'Abg_pdb', 'Acp_ideal', 'Ad2_pdb', 'Adm_ideal', 'Adm_pdb',
'Adp_ideal', 'Adp_pdb', 'Adu_pdb', 'Aee_ideal', 'Af3_ideal',
'Agh_ideal', 'Agh_pdb', 'Ahz_ideal', 'Ahz_pdb', 'Ait_pdb', 'Al5_pdb',
'Al9_pdb', 'Am6_pdb', 'Amk_ideal', 'An2_pdb', 'An9_ideal',
'Any_ideal', 'Ap5_ideal', 'Ap5_pdb', 'Ap8_ideal', 'Ap8_pdb',
'Apc_pdb', 'Apn_ideal', 'Apn_pdb', 'Aq4_pdb', 'Ard_ideal',
'At4_ideal', 'At4_pdb', 'Ax2_ideal', 'Axq_pdb', 'Axt_pdb', 'Azr_pdb',
'B3p_ideal', 'B4p_pdb', 'B7n_pdb', 'Ba3_ideal', 'Bah_ideal',
'Bah_pdb', 'Bak_ideal', 'Bak_pdb', 'Bay_ideal', 'Bay_pdb', 'Bbb_pdb',
'Bcx_pdb', 'Be3_ideal', 'Be6_ideal', 'Bed_ideal', 'Bee_pdb',
'Bei_ideal', 'Bei_pdb', 'Bej_ideal', 'Bg3_pdb', 'Bgf_ideal',
'Bgx_pdb', 'Bh1_ideal', 'Bh1_pdb', 'Bhc_pdb', 'Bhm_ideal', 'Bhm_pdb',
'Biz_pdb', 'Bl0_pdb', 'Blg_pdb', 'Bmp_pdb', 'Bmq_pdb', 'Bn1_pdb',
'Bnz_ideal', 'Bnz_pdb', 'Boe_pdb', 'Bpb_pdb', 'Bph_pdb', 'Brh_pdb',
'Brx_ideal', 'Bse_pdb', 'Bsj_ideal', 'Bt3_pdb', 'Bth_ideal',
'Bv2_ideal', 'Bv4_ideal', 'By6_ideal', 'C15_ideal', 'C1a_ideal',
```

```
'C1a_pdb', 'C1x_pdb', 'C2e_ideal', 'C2l_pdb', 'C2n_ideal', 'C2r_pdb',
'C35_ideal', 'C40_ideal', 'C60_ideal', 'C66_pdb', 'C94_ideal',
'Ca3_pdb', 'Ca5_pdb', 'Cag_pdb', 'Cai_pdb', 'Cb1_ideal', 'Cb1_pdb',
'Cba_ideal', 'Cba_pdb', 'Cbc_pdb', 'Cco_ideal', 'Cdb_ideal',
'Cdl_ideal', 'Ce6_pdb', 'Ceh_pdb', 'Cfa_ideal', 'Cgq_ideal',
'Cgq_pdb', 'Ch7_pdb', 'Chx_ideal', 'Chx_pdb', 'Cis_ideal', 'Cis_pdb',
'Cll_pdb', 'Cmr_pdb', 'Cni_ideal', 'Cns_ideal', 'Cnt_pdb',
'Co8_ideal', 'Co8_pdb', 'Cof_ideal', 'Cof_pdb', 'Con_pdb',
'Cot_ideal', 'Cp3_pdb', 'Cp4_ideal', 'Cpd_ideal', 'Cr7_pdb',
'Crt_pdb', 'Cru_pdb', 'Crw_pdb', 'Crx_pdb', 'Cs7_pdb', 'Csl_pdb',
'Ct3_ideal', 'Cty_pdb', 'Cya_pdb', 'Cyj_pdb', 'Cyo_pdb', 'D1r_ideal',
'D2v_pdb', 'D3p_pdb', 'D4p_pdb', 'D94_pdb', 'Dad_ideal', 'Dat_pdb',
'Dbl_ideal', 'Dbu_pdb', 'Dcc_pdb', 'Ddt_pdb', 'Ddx_pdb', 'Ddy_ideal',
'Dec_ideal', 'Deq_ideal', 'Deq_pdb', 'Dgd_ideal', 'Dgh_pdb',
'Dgi_ideal', 'Dgs_pdb', 'Dhc_pdb', 'Dih_ideal', 'Dit_ideal',
'Dit_pdb', 'Dk1_ideal', 'Dk1_pdb', 'Dme_ideal', 'Do3_pdb', 'Doi_pdb',
'Dot_pdb', 'Dpg_ideal', 'Dpy_pdb', 'Dr6_ideal', 'Dr8_ideal',
'Dr8_pdb', 'Dr9_ideal', 'Drp_pdb', 'Drr_ideal', 'Drz_pdb', 'Dss_pdb',
'Dsu_pdb', 'Dtg_pdb', 'Dx9_ideal', 'E10_pdb', 'E1x_pdb', 'E55_ideal',
'E55_pdb', 'E7b_pdb', 'Ebs_ideal', 'Ebw_ideal', 'Ede_ideal',
'Ede_pdb', 'Eh5_ideal', 'Eli_ideal', 'Emp_pdb', 'Enp_ideal',
'Eob_pdb', 'Eov_pdb', 'Eph_ideal', 'Eph_pdb', 'Esd_pdb', 'Ets_ideal',
'F1g_ideal', 'F1p_ideal', 'F3b_ideal', 'F3b_pdb', 'F42_pdb',
'Fa2_pdb', 'Faa_ideal', 'Faa_pdb', 'Fab_ideal', 'Fam_pdb', 'Fcl_pdb',
'Fd4_pdb', 'Fdm_pdb', 'Fgp_pdb', 'Fhl_pdb', 'Fhu_ideal', 'Fka_ideal',
'Fld_ideal', 'Fld_pdb', 'Fnh_pdb', 'Fns_ideal', 'Fns_pdb', 'Fox_pdb',
'Fri_ideal', 'Frn_ideal', 'Frn_pdb', 'Fro_pdb', 'Fti_pdb', 'Fwd_pdb',
'Fyn_pdb', 'G25_pdb', 'G2l_pdb', 'G5p_pdb', 'G6p_pdb', 'G6q_ideal',
'G80_pdb', 'Gc4_pdb', 'Gcd_pdb', 'Gcg_ideal', 'Gda_pdb', 'Gds_ideal',
'Gdx_ideal', 'Ggd_ideal', 'Ggt_ideal', 'Gke_ideal', 'Gmm_ideal',
'Gms_pdb', 'Gne_pdb', 'Gp2_pdb', 'Gp5_pdb', 'Gpn_ideal', 'Gtt_ideal',
'Gu1_pdb', 'Gu2_pdb', 'Gu4_ideal', 'Gu4_pdb', 'Gu8_pdb', 'Gu9_pdb',
'H3s_ideal', 'H3s_pdb', 'H4p_ideal', 'H5p_pdb', 'Ha1_ideal',
'Had_pdb', 'Hbp_ideal', 'Hbp_pdb', 'Hbu_pdb', 'Hdc_ideal', 'Hdp_pdb',
'Heh_ideal', 'Heu_pdb', 'Hfb_ideal', 'Hfb_pdb', 'Hft_ideal',
'Hg9_pdb', 'Hgp_ideal', 'Hi6_ideal', 'Hi6_pdb', 'Hiq_pdb',
'Hlo_ideal', 'Hlo_pdb', 'Hmg_ideal', 'Hmg_pdb', 'Hob_pdb', 'Hol_pdb',
'Hoq_pdb', 'Hqp_pdb', 'Hs6_pdb', 'Hsx_pdb', 'Htc_pdb', 'Hxl_ideal',
'Hyb_pdb', 'I22_pdb', 'I5c_pdb', 'Ia_pdb', 'Iam_pdb', 'Icr_pdb',
'Idb_ideal', 'Idt_pdb', 'Igu_pdb', 'Ilf_ideal', 'Imk_pdb', 'Imo_pdb',
'Imt_pdb', 'Inm_pdb', 'Iog_pdb', 'Ipb_pdb', 'Ipf_pdb', 'Ipn_pdb',
'Ist_pdb', 'Itl_ideal', 'Iu_pdb', 'Ium_pdb', 'Iwd_ideal', 'Jh0_ideal',
'Jos_ideal', 'Js5_pdb', 'K1r_pdb', 'Ki2_pdb', 'L04_ideal', 'L0i_pdb',
'L1p_ideal', 'L1p_pdb', 'L3g_pdb', 'L3p_ideal', 'L4p_ideal', 'Lc_pdb',
'Lch_pdb', 'Led_pdb', 'Let_pdb', 'Lg6_ideal', 'Lg_pdb', 'Lha_pdb',
'Lhu_ideal', 'Lhu_pdb', 'Lil_pdb', 'Lio_ideal', 'Lj1_pdb', 'Lmg_pdb',
'Lng_pdb', 'Lop_ideal', 'Lso_pdb', 'Lsp_ideal', 'Lsp_pdb', 'Lut_pdb',
'Lux_pdb', 'M0e_ideal', 'M0n_ideal', 'M54_pdb', 'M5m_pdb', 'M6d_pdb',
'M7g_ideal', 'M98_ideal', 'M99_ideal', 'Mar_pdb', 'Mat_pdb',
'Mau_pdb', 'Mav_pdb', 'Mcr_pdb', 'Mfb_pdb', 'Mfc_pdb', 'Mgu_pdb',
'Mji_pdb', 'Mm0_pdb', 'Mm6_pdb', 'Mmo_pdb', 'Mnu_pdb', 'Mox_pdb',
'Mpn_pdb', 'Mq7_ideal', 'Mq8_pdb', 'Mq9_pdb', 'Mrg_pdb', 'Ms3_ideal',
'Mtr_pdb', 'Mts_pdb', 'Mtz_ideal', 'My9_ideal', 'Mye_pdb',
'Mys_ideal', 'Myy_ideal', 'N3c_ideal', 'N6g_pdb', 'Na1_pdb',
'Nbp_ideal', 'Ncn_ideal', 'Ncq_pdb', 'Nde_pdb', 'Ndn_pdb',
'Net_ideal', 'Nex_ideal', 'Nf2_pdb', 'Nf_ideal', 'Nf_pdb',
'Ng6_ideal', 'Ng6_pdb', 'Ngk_ideal', 'Ngu_ideal', 'Nh3_ideal',
'Nii_ideal', 'Nlt_pdb', 'Nlx_ideal', 'Nms_pdb', 'Nst_ideal',
'Nta_ideal', 'Ntd_pdb', 'Ntn_pdb', 'Nup_pdb', 'O2c_pdb', 'Obi_ideal',
'Obi_pdb', 'Obs_pdb', 'Oca_pdb', 'Oin_pdb', 'Omp_ideal', 'Omp_pdb',
```

'Opt_pdb', 'Otp_ideal', 'Otp_pdb', 'Oxg_ideal', 'Oxg_pdb', 'Oxi_pdb',
'P14_ideal', 'P2t_pdb', 'P33_ideal', 'P3a_ideal', 'P3a_pdb',
'P3s_pdb', 'P6g_ideal', 'Pax_ideal', 'Pax_pdb', 'Pbm_pdb', 'Pbt_pdb',
'Pck_ideal', 'Pcu_ideal', 'Pcu_pdb', 'Pcz_pdb', 'Pdi_ideal',
'Pdj_ideal', 'Pe3_ideal', 'Pe8_ideal', 'Peh_pdb', 'Peu_ideal',
'Peu_pdb', 'Pfg_ideal', 'Pg7_pdb', 'Pgk_ideal', 'Pgk_pdb',
'Pgn_ideal', 'Pgp_pdb', 'Pgv_pdb', 'Pgw_ideal', 'Phg_ideal',
'Phg_pdb', 'Phy_pdb', 'Pi0_pdb', 'Pi3_pdb', 'Pic_ideal', 'Pin_ideal',
'Pin_pdb', 'Plc_pdb', 'Plx_ideal', 'Pma_pdb', 'Pmc_ideal',
'Pmm_ideal', 'Pmm_pdb', 'Pnp_ideal', 'Pnt_pdb', 'Po4_ideal',
'Poh_pdb', 'Pon_pdb', 'Pop_pdb', 'Ppw_pdb', 'Pqe_pdb', 'Pr5_pdb',
'Prt_pdb', 'Ps2_ideal', 'Ps2_pdb', 'Psc_pdb', 'Psl_ideal', 'Psl_pdb',
'Psp_ideal', 'Psp_pdb', 'Ptc_pdb', 'Ptn_pdb', 'Pua_ideal', 'Pua_pdb',
'Pxt_pdb', 'Py3_ideal', 'Pyb_pdb', 'Pyn_pdb', 'Q2y_pdb', 'Qkh_pdb',
'R96_pdb', 'Raa_pdb', 'Rad_pdb', 'Ral_ideal', 'Rb3_pdb', 'Rf5_ideal',
'Rgc_ideal', 'Rh9_ideal', 'Rif_pdb', 'Ril_pdb', 'Rj1_ideal',
'Rox_ideal', 'Rp5_ideal', 'Rpa_pdb', 'Rr6_ideal', 'Rrc_ideal',
'Rsd_ideal', 'Rsd_pdb', 'Rx3_pdb', 'S2m_pdb', 'Scd_ideal',
'Sco_ideal', 'Se4_pdb', 'Sei_pdb', 'Sfp_ideal', 'Sfp_pdb',
'Sft_ideal', 'Sft_pdb', 'Sg3_pdb', 'Shi_ideal', 'Sht_ideal',
'Sil_pdb', 'Smp_pdb', 'Sn6_ideal', 'Sn6_pdb', 'Sn7_ideal', 'Sn7_pdb',
'So3_ideal', 'Soy_pdb', 'Sp2_pdb', 'Spl_pdb', 'Spx_ideal',
'Sqd_ideal', 'Squ_ideal', 'Ssu_pdb', 'Stc_ideal', 'Std_pdb',
'Sud_ideal', 'Suj_pdb', 'Suw_pdb', 'Sxn_pdb', 'Syb_ideal', 'Syb_pdb',
'T10_ideal', 'T2s_pdb', 'T2t_pdb', 'T36_pdb', 'T49_pdb', 'T5a_ideal',
'T5a_pdb', 'T66_pdb', 'Ta4_pdb', 'Tao_pdb', 'Tb0_ideal', 'Tb0_pdb',
'Tc1_pdb', 'Tce_ideal', 'Tcn_ideal', 'Tcn_pdb', 'Tcz_ideal',
'Tcz_pdb', 'Tdt_ideal', 'Tee_pdb', 'Tel_pdb', 'Tfe_pdb', 'Tfo_pdb',
'Tft_ideal', 'Tft_pdb', 'Tgl_pdb', 'Tgt_pdb', 'Th1_pdb', 'Th3_ideal',
'Thj_ideal', 'Thj_pdb', 'Ths_pdb', 'Thx_ideal', 'Tlb_pdb',
'Tma_ideal', 'Tma_pdb', 'Tot_ideal', 'Tot_pdb', 'Tp8_ideal',
'Tp8_pdb', 'Tp9_ideal', 'Tp9_pdb', 'Tsu_ideal', 'Tt2_pdb', 'Ttd_pdb',
'Ttm_pdb', 'Twt_ideal', 'Tyo_ideal', 'Tyz_pdb', 'U02_ideal',
'U2f_ideal', 'U2f_pdb', 'U2l_pdb', 'U2p_ideal', 'U2p_pdb', 'U34_pdb',
'U37_ideal', 'U3h_ideal', 'U5p_ideal', 'U8u_pdb', 'Ub1_ideal',
'Ubb_pdb', 'Ucb_pdb', 'Ucl_ideal', 'Ud5_pdb', 'Uda_ideal', 'Uda_pdb',
'Udm_pdb', 'Udx_ideal', 'Uga_ideal', 'Uir_ideal', 'Ula_ideal',
'Ums_pdb', 'Up1_pdb', 'Up5_pdb', 'Up6_ideal', 'Upp_ideal', 'Uq6_pdb',
'Uq7_ideal', 'Uq7_pdb', 'Uq8_ideal', 'Uq_ideal', 'Ur3_pdb', 'Vag_pdb',
'Van_ideal', 'Vg6_pdb', 'Wy2_pdb', 'Wz4_pdb', 'X7o_pdb', 'Xat_pdb',
'Xmd_ideal', 'Xmj_pdb', 'Xpe_ideal', 'Xpe_pdb', 'Z23_ideal',
'Z23_pdb', 'Z_pdb', 'Zdu_pdb', 'Zmo_ideal', 'Zth_ideal']

& So, from 13045 jobs that were clean, those below (388 in total) would have failed according to the new modified SQM (but not used here).

>>>Mol Jobs whose sqm.out has 'No convergence in SCF' but finished OK<<<

388 ['01k_ideal', '01k_pdb', '10a_ideal', '118_pdb', '12p_ideal',
'141_ideal', '15m_pdb', '16p_ideal', '198_ideal', '1un_ideal',
'2ba_pdb', '2bc_pdb', '2nc_ideal', '2nc_pdb', '2pe_ideal',
'2sp_ideal', '34d_ideal', '3an_ideal', '3bi_ideal', '3cp_pdb',
'3ep_ideal', '3ph_ideal', '471_pdb', '4pt_ideal', '4su_ideal',
'4su_pdb', '4tc_ideal', '515_pdb', '587_ideal', '5bu_ideal',
'5fu_ideal', '5iu_ideal', '693_ideal', '6pl_ideal', '6ul_ideal',
'6ul_pdb', '70u_pdb', '750_pdb', '757_ideal', '773_ideal',
'784_ideal', '7ni_ideal', '7ph_ideal', '852_ideal', '858_pdb',
'8pp_pdb', '941_pdb', 'A1r_ideal', 'A2p_pdb', 'A2r_ideal',

```
'A2t_ideal', 'A3p_ideal', 'A6p_pdb', 'A76_ideal', 'A79_pdb',
'A85_pdb', 'A9a_pdb', 'Aa7_ideal', 'Aa7_pdb', 'Aao_ideal', 'Abf_pdb',
'Abg_pdb', 'Acp_ideal', 'Adm_ideal', 'Adm_pdb', 'Adp_ideal',
'Adp_pdb', 'Aee_ideal', 'Ahz_ideal', 'Ahz_pdb', 'Ait_pdb', 'Am6_pdb',
'Amk_ideal', 'An2_pdb', 'An9_ideal', 'Ap5_ideal', 'Ap5_pdb',
'Apc_pdb', 'Apn_ideal', 'At4_ideal', 'At4_pdb', 'Ax2_ideal',
'Axq_pdb', 'Azr_pdb', 'B3p_ideal', 'B7n_pdb', 'Ba3_ideal', 'Bah_pdb',
'Bak_ideal', 'Bak_pdb', 'Be3_ideal', 'Be6_ideal', 'Bed_ideal',
'Bee_pdb', 'Bei_ideal', 'Bei_pdb', 'Bej_ideal', 'Bhc_pdb',
'Bhm_ideal', 'Bhm_pdb', 'Biz_pdb', 'Bl0_pdb', 'Blg_pdb', 'Bmp_pdb',
'Bmq_pdb', 'Bnz_ideal', 'Bnz_pdb', 'Bsj_ideal', 'Bt3_pdb',
'C15_ideal', 'C1a_ideal', 'C2e_ideal', 'C2n_ideal', 'C2r_pdb',
'C35_ideal', 'C40_ideal', 'Ca3_pdb', 'Ca5_pdb', 'Cb1_ideal',
'Cba_ideal', 'Cbc_pdb', 'Cco_ideal', 'Ce6_pdb', 'Ceh_pdb',
'Cfa_ideal', 'Cgq_ideal', 'Chx_ideal', 'Chx_pdb', 'Cis_ideal',
'Cis_pdb', 'Cll_pdb', 'Cni_ideal', 'Cns_ideal', 'Co8_ideal',
'Co8_pdb', 'Cof_ideal', 'Cof_pdb', 'Con_pdb', 'Cot_ideal', 'Cp3_pdb',
'Cp4_ideal', 'Cpd_ideal', 'Cs7_pdb', 'Ct3_ideal', 'D1r_ideal',
'D2v_pdb', 'Dad_ideal', 'Dat_pdb', 'Dbl_ideal', 'Dcc_pdb', 'Ddt_pdb',
'Ddy_ideal', 'Dec_ideal', 'Dih_ideal', 'Dk1_ideal', 'Dk1_pdb',
'Dme_ideal', 'Do3_pdb', 'Doi_pdb', 'Dpg_ideal', 'Dr9_ideal',
'Dss_pdb', 'Dsu_pdb', 'Dx9_ideal', 'E10_pdb', 'E7b_pdb', 'Ebs_ideal',
'Ebw_ideal', 'Ede_ideal', 'Eh5_ideal', 'Eph_ideal', 'Eph_pdb',
'F1p_ideal', 'F3b_ideal', 'F3b_pdb', 'F42_pdb', 'Fam_pdb', 'Fd4_pdb',
'Fdm_pdb', 'Fhu_ideal', 'Fka_ideal', 'Fnh_pdb', 'Fns_pdb',
'Fri_ideal', 'Fro_pdb', 'Fwd_pdb', 'Fyn_pdb', 'G6p_pdb', 'G6q_ideal',
'Gcg_ideal', 'Gds_ideal', 'Ggt_ideal', 'Gke_ideal', 'Gp2_pdb',
'Gtt_ideal', 'Gu4_ideal', 'Gu4_pdb', 'H3s_ideal', 'H3s_pdb',
'H4p_ideal', 'H5p_pdb', 'Hbu_pdb', 'Hdc_ideal', 'Hfb_ideal',
'Hfb_pdb', 'Hft_ideal', 'Hgp_ideal', 'Hmg_ideal', 'Hmg_pdb',
'Hs6_pdb', 'Hsx_pdb', 'Htc_pdb', 'Hyb_pdb', 'Ia_pdb', 'Icr_pdb',
'Idb_ideal', 'Imo_pdb', 'Iog_pdb', 'Ipb_pdb', 'Ipf_pdb', 'Itl_ideal',
'Iwd_ideal', 'Jos_ideal', 'L04_ideal', 'L1p_ideal', 'Lg6_ideal',
'Lha_pdb', 'Lhu_ideal', 'Lil_pdb', 'Lio_ideal', 'Lj1_pdb', 'Lmg_pdb',
'Lop_ideal', 'Lsp_ideal', 'Lsp_pdb', 'Lut_pdb', 'M6d_pdb', 'Mar_pdb',
'Mau_pdb', 'Mm0_pdb', 'Mm6_pdb', 'Mox_pdb', 'Mpn_pdb', 'Mq7_ideal',
'Mq9_pdb', 'Ms3_ideal', 'Mts_pdb', 'Mtz_ideal', 'My9_ideal',
'Mys_ideal', 'Myy_ideal', 'N3c_ideal', 'Nbp_ideal', 'Ncq_pdb',
'Ndn_pdb', 'Net_ideal', 'Nex_ideal', 'Nf_pdb', 'Ng6_ideal', 'Ng6_pdb',
'Ngk_ideal', 'Ngu_ideal', 'Nh3_ideal', 'Nii_ideal', 'Nlt_pdb',
'Nlx_ideal', 'Nst_ideal', 'Nta_ideal', 'Ntd_pdb', 'Nup_pdb',
'Omp_ideal', 'Omp_pdb', 'Otp_pdb', 'Oxg_ideal', 'Oxg_pdb',
'P14_ideal', 'P33_ideal', 'P3s_pdb', 'P6g_ideal', 'Pbm_pdb',
'Pdi_ideal', 'Pdj_ideal', 'Pe3_ideal', 'Pe8_ideal', 'Pfg_ideal',
'Pgk_ideal', 'Pgv_pdb', 'Pgw_ideal', 'Phg_ideal', 'Phg_pdb',
'Phy_pdb', 'Pi0_pdb', 'Pi3_pdb', 'Pic_ideal', 'Pin_ideal',
'Plx_ideal', 'Pma_pdb', 'Pmc_ideal', 'Pmm_ideal', 'Pmm_pdb',
'Pnp_ideal', 'Po4_ideal', 'Pon_pdb', 'Pop_pdb', 'Psl_ideal',
'Psl_pdb', 'Pua_ideal', 'Pua_pdb', 'Pyn_pdb', 'R96_pdb', 'Rad_pdb',
'Ral_ideal', 'Rf5_ideal', 'Rj1_ideal', 'Rox_ideal', 'Rp5_ideal',
'Rr6_ideal', 'Rrc_ideal', 'Scd_ideal', 'Sco_ideal', 'Se4_pdb',
'Sei_pdb', 'Sft_ideal', 'Sg3_pdb', 'Shi_ideal', 'Sht_ideal',
'So3_ideal', 'Sp2_pdb', 'Sqd_ideal', 'Squ_ideal', 'Stc_ideal',
'Sud_ideal', 'Syb_ideal', 'Syb_pdb', 'T10_ideal', 'T49_pdb',
'T5a_ideal', 'T5a_pdb', 'Tb0_ideal', 'Tb0_pdb', 'Tce_ideal',
'Tcn_ideal', 'Tcn_pdb', 'Tcz_ideal', 'Tcz_pdb', 'Tft_ideal',
'Tgt_pdb', 'Thj_ideal', 'Thj_pdb', 'Ths_pdb', 'Thx_ideal',
'Tma_ideal', 'Tma_pdb', 'Tp9_ideal', 'Tp9_pdb', 'Tsu_ideal',
'Tt2_pdb', 'Twt_ideal', 'U2f_ideal', 'U2f_pdb', 'U2p_ideal',
'U2p_pdb', 'U37_ideal', 'U3h_ideal', 'U5p_ideal', 'Ub1_ideal',
```

```
'Ucl_ideal', 'Ud5_pdb', 'Uda_ideal', 'Uda_pdb', 'Udm_pdb',
'Udx_ideal', 'Uga_ideal', 'Up5_pdb', 'Up6_ideal', 'Upp_ideal',
'Uq7_pdb', 'Uq8_ideal', 'Uq_ideal', 'Vag_pdb', 'Vg6_pdb', 'Xat_pdb',
'Xmd_ideal', 'Xmj_pdb', 'Xpe_ideal', 'Xpe_pdb', 'Zmo_ideal',
'Zth_ideal']

>>> Time Job Execution Summary <<<

Number of clean jobs: 13045
Longest job: Mol='Rad_pdb', time= 9h 59m 46s
Fatest job: Mol='Mmc_ideal', time= 0s
Average time of execution per clean job: 14m 35s

Total number of jobs: 17582
Global average time of execution per job: 26m 17s
```
