# Supplementary figures and images for: ACPYPE - AnteChamber PYthon Parser interfacE
Source: BMC Res Notes. 2012 Jul 23;5:367. doi: 10.1186/1756-0500-5-367 (PMC3461484; doi:10.1186/1756-0500-5-367)

1iku

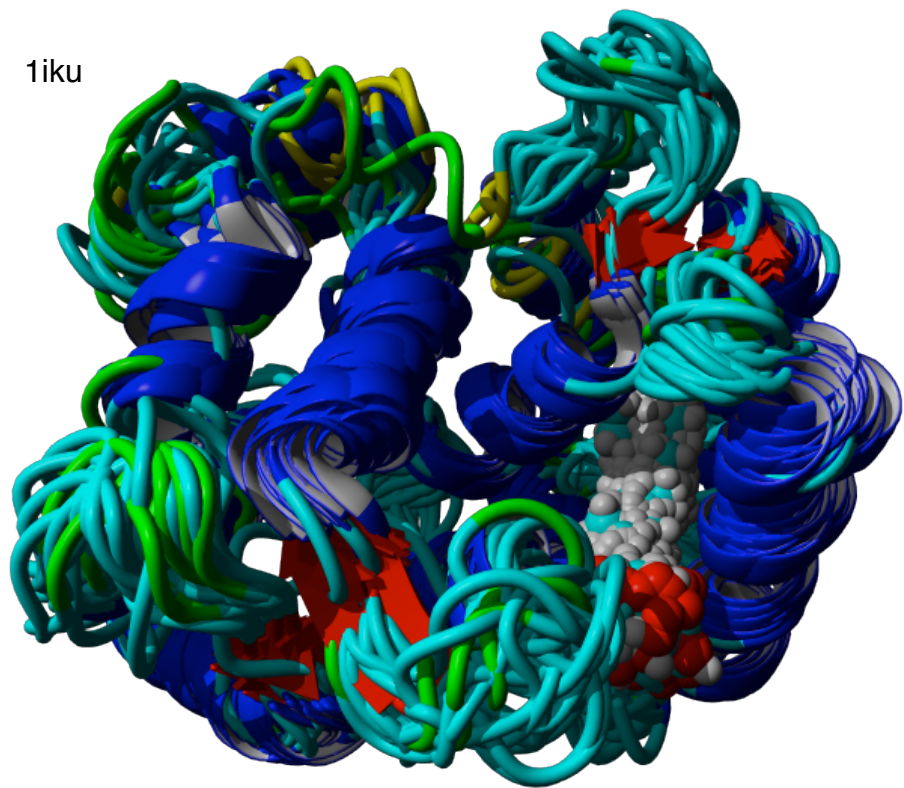

2k0g

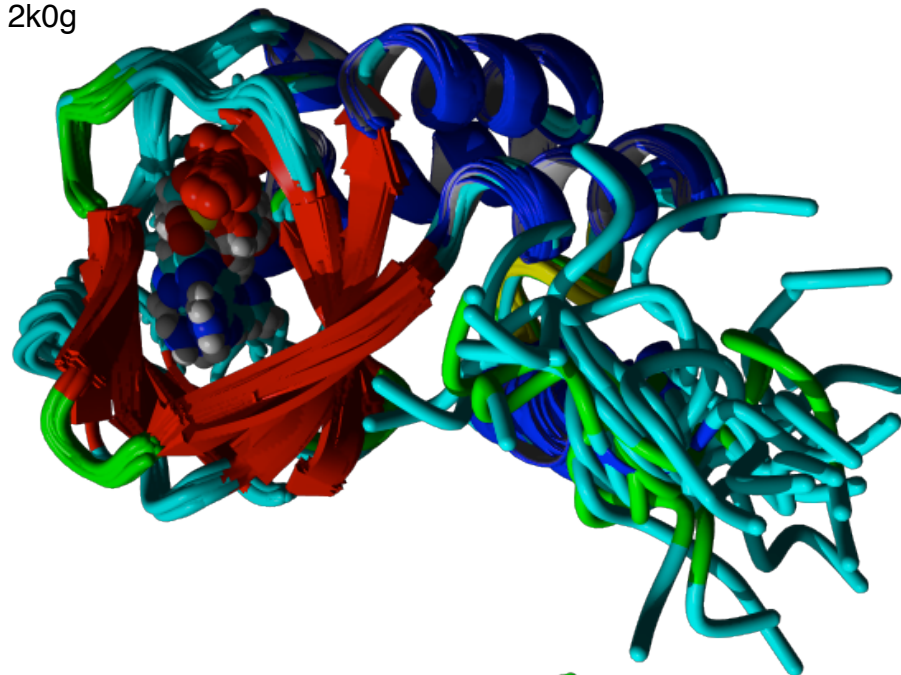

2jn3

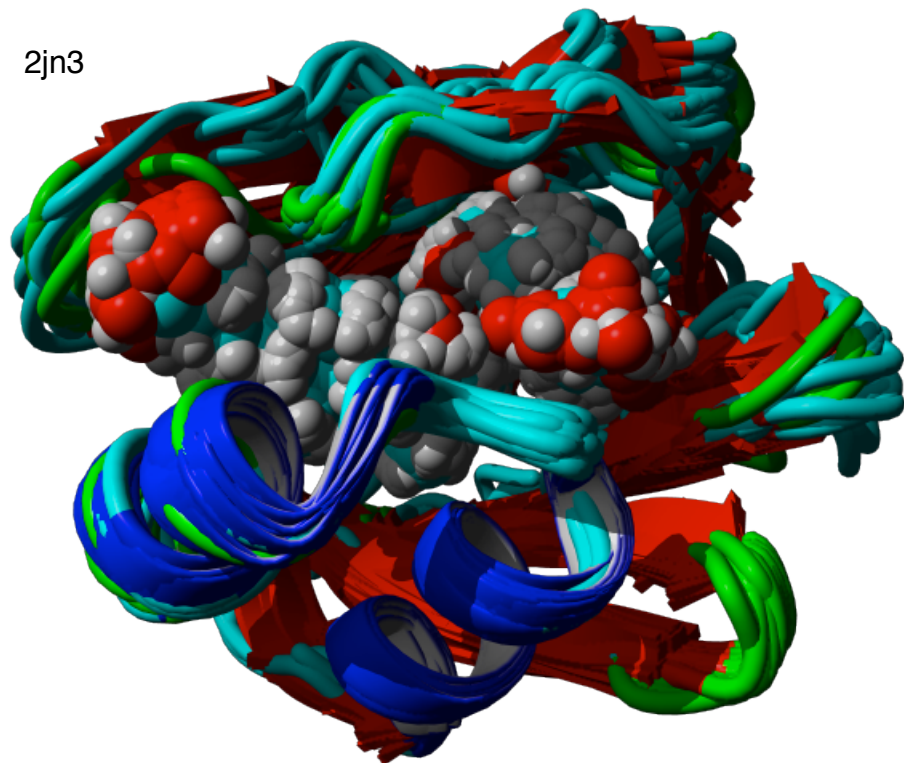

1jkn

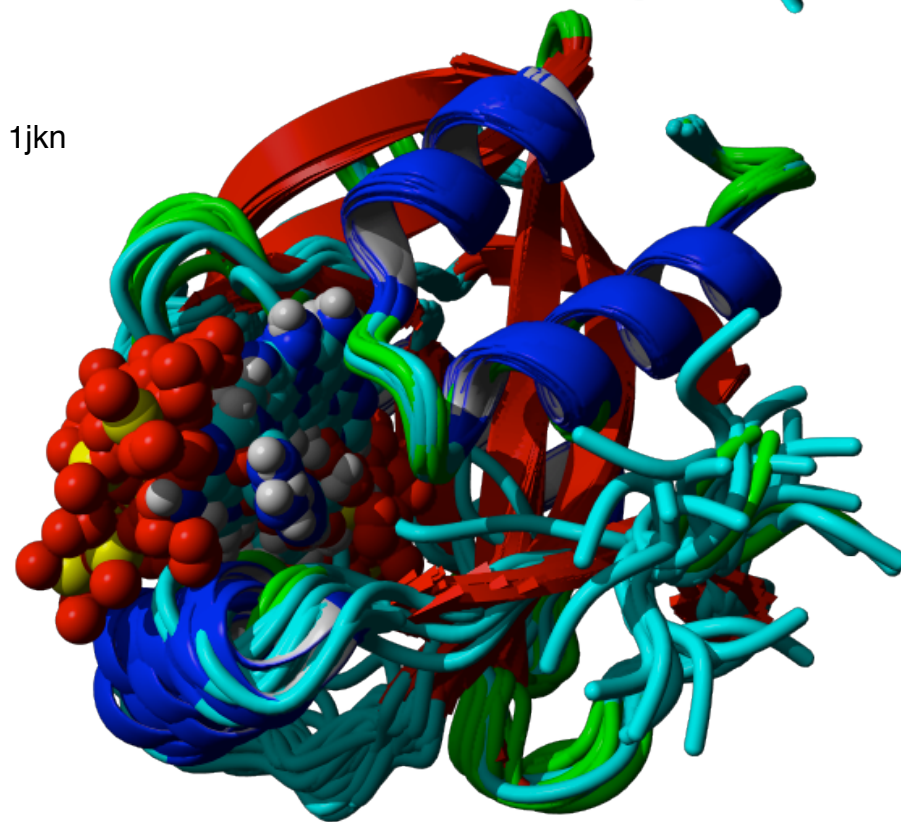

Supplement: Additional file 4 — Figures for recalculated entries [PDB:1IKU], [PDB:1JKN], [PDB:2JN3] and [PDB:2K0G]. [file 1756-0500-5-367-S4.pdf]
